# Structurally Simple Phenanthridine Analogues Based on Nitidine and Their Antitumor Activities

**DOI:** 10.3390/molecules24030437

**Published:** 2019-01-26

**Authors:** Shu-Qin Qin, Lian-Chun Li, Jing-Ru Song, Hai-Yun Li, Dian-Peng Li

**Affiliations:** 1 Guangxi Key Laboratory of Functional Phytochemicals Research and Utilization, Guangxi Institute of Botany, Chinese Academy of Sciences, Guilin 541006, Guangxi, China; shuqinshu@163.com (S.-Q.Q.); 15577498821@163.com (L.-C.L.); 2 College of Chemistry and Bioengineering, Guilin University of Technology, Guilin 541006, Guangxi, China; lihaiyun@glut.edu.cn

**Keywords:** nitidine, phenanthridine, phenanthridinone, antitumor activity

## Abstract

A series of novel structurally simple analogues based on nitidine was designed and synthesized in search of potent anticancer agents. The antitumor activity against human cancer cell lines (HepG2, A549, NCI-H460, and CNE1) was performed by 3-(4,5-dimethylthiazol-2-yl)-2,5-diphenyl tetrazolium bromide (MTT) assay in vitro. The results showed that some of them had good anticancer activities, especially derivatives with a [(dimethylamino)ethyl]amino side chain in the C-6 position. Planar conjugated compounds **15a**, **15b**, and **15c**, with IC_50_ values of 1.20 μM, 1.87 μM, and 1.19 μM against CNE1 cells, respectively, were more active than nitidine chloride. Compound **15b** and compound **15c** with IC_50_ values of 1.19 μM and 1.37 μM against HepG2 cells and A549 cells demonstrated superior activities to nitidine. Besides, compound **5e** which had a phenanthridinone core displayed extraordinary cytotoxicity against all test cells, particularly against CNE1 cells with the IC_50_ value of 1.13 μM.

## 1. Introduction

Nowadays, cancer is a major public health issue in most of countries with high mortality rates [1,2,3]. Therefore, it is necessary and urgent to develop novel antitumor drugs with higher activity but lower toxicity. Natural products and their related derivatives have been proved to be important candidates in the discovery and development of bioactive drugs owing to the lower mammalian toxicity [4]. Quaternary benzo[*c*]phenanthridines are alkaloids with extensive bioactivities [5,6,7,8,9]. Among them, natural occurring nitidine, fagaronine, chelerythrine, and sanguinarine exhibit good antitumor activity. The synthesis of their derivatives is of great interest because of the lower activity in vivo owing to the instability of the iminium salt moiety in the structure [10,11,12]. As a synthetic derivative of benzo[*c*]phenanthridine, NK 314 displays high antitumor activity not only in vitro but also in vivo and can be regarded as a promising anticancer drug which inspired chemist to develop more structurally modified or structurally simple alternatives [13,14,15,16].

Nitidine, as a best-known member of benzo[*c*]phenanthridine alkaloids, has received much attention because of its broad range of biological activities, including anti-inflammatory, anti-malarial, antibacterial, anti-HIV, and especially antitumor [17,18,19,20,21,22,23,24,25,26,27,28,29]. Despite the excellent activity against the cancer cells, little progress has been made, attributed to the unsatisfactory activity in vivo and complicated total synthetic route. As shown in the structure, phenanthridine is the common but significant N-heterocyclic core existing in benzo[*c*]phenanthridine and some drugs [30,31,32]. Thus, structurally simple phenanthridine alternatives may display similar antitumor activity. Considering the necessity of 8,9-dimethoxy groups and steric interaction on the skeletons for the biological effects from the QSAR model based on benzo[*c*]phenanthridine [33], a class of analogues based on nitidine were designed and synthesized. The antitumor activity was evaluated against human cell lines of HepG2, A549, NCI-H460, and CNE1 in vitro.

## 2. Results and Discussion

### 2.1. Synthesis

**Scheme 1:**
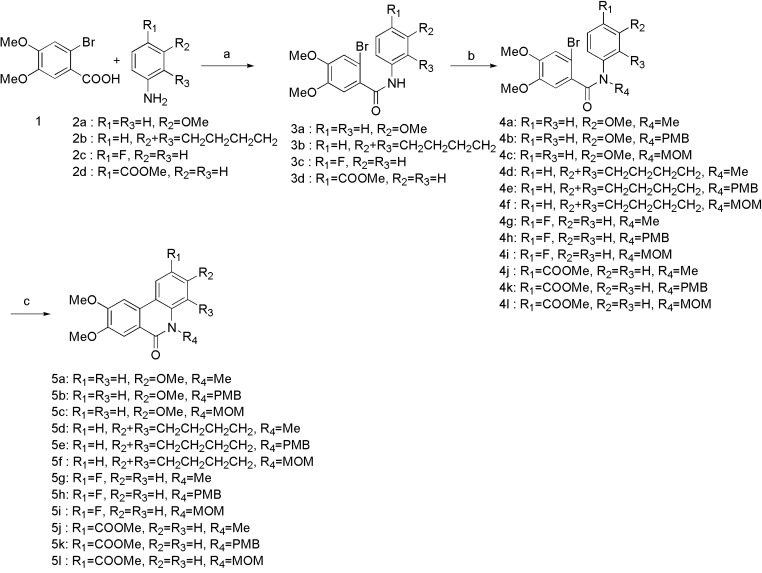

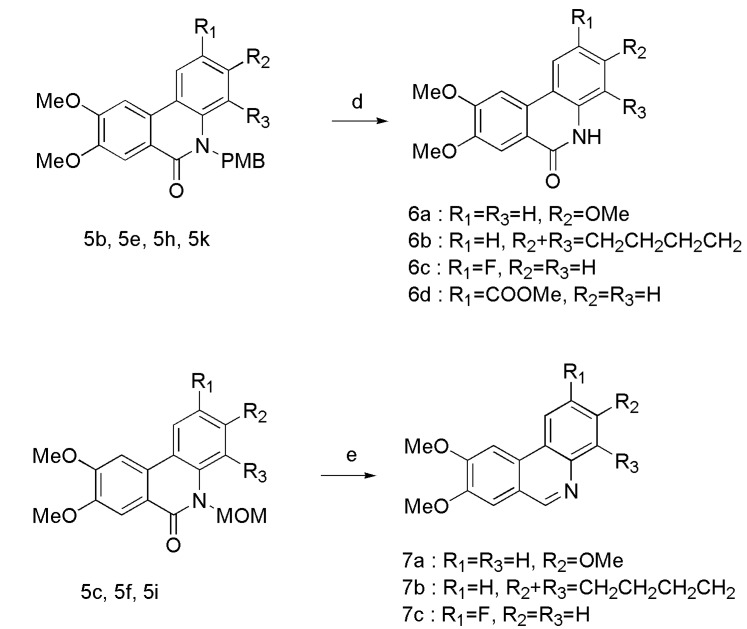
Reagents and conditions: (**a**) SOCl_2_, ^i^PrNEt, DCM, reflux, 78–91%; (**b**) NaH, DMF, PMBCl or MOMCl, rt, 92–98%; (**c**) Pd(oAc), P(*o*-tol)_3_, K_2_CO_3_, DMF, 155 °C, 78–90%; (**d**) TFA, 75 °C, 54–78%; (**e**) LiAlH_4_, dry THF, 0 °C-rt, 45–55%.

**Scheme 2:**
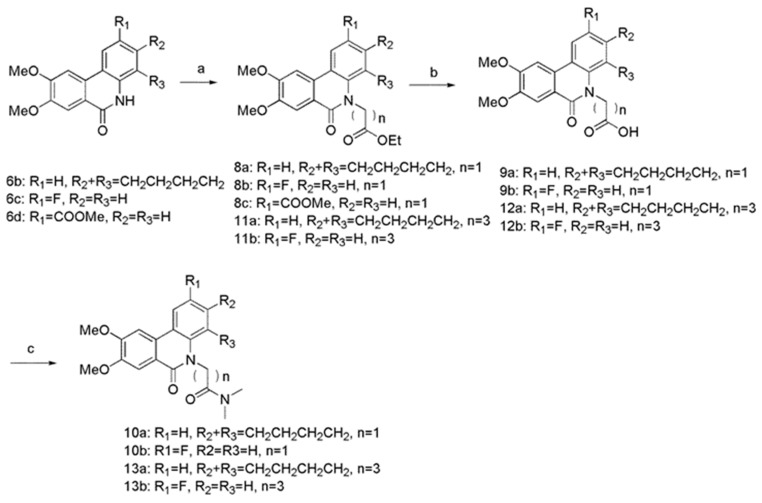
Reagents and conditions: (**a**) ethyl bromoacetate or ethyl 4-bromobutyrate, Cs_2_CO_3_, TBAI, DMF, 90 °C, 76–95%; (**b**) MeOH, H_2_O, 10N NaOH, rt, 93–98%; (**c**) triethylamine, THF, CH_2_Cl_2_, isobutyl chloroformate, dimethylamine, 0 °C-rt, 82–85%.

**Scheme 3:**
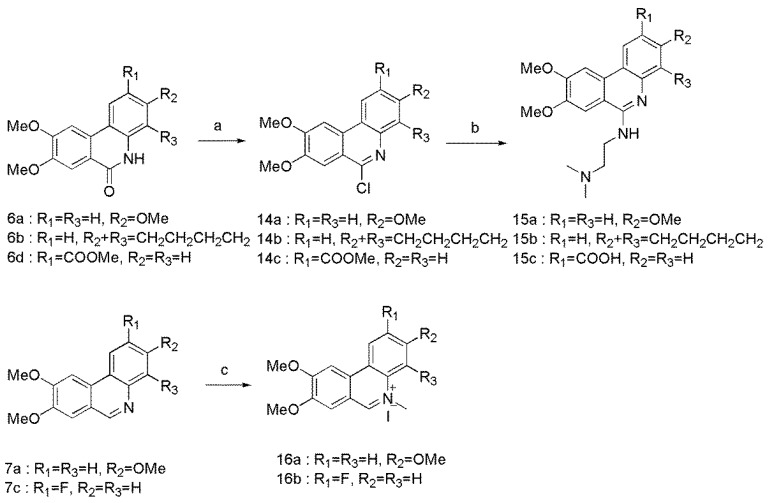
Reagents and conditions: (**a**) POCl_3_, 105 °C, 83–85%; (**b**) Me_2_N(CH_2_)_2_NH_2_, reflux, 59–78%. (**c**) MeI, toluene, 80 °C, 75–84%.

The analogues were synthesized according to the procedures in Scheme 1, Scheme 2 and Scheme 3. Firstly, the 2-bromo-4,5-dimethoxybenzoic acid was converted to acid chloride in situ by refluxing in SOCl_2_. After evaporation of SOCl_2_ under reduced pressure, the acid chloride was directly reacted with various commercially available anilines (**2a**–**d**) in dry DCM. Different groups were introduced to obtain corresponding N-protected compounds **4a**–**l**. Intramolecular Heck coupling in the presence of Pd(oAc)_2_/P(*o*-tol)_3_/K_2_CO_3_/DMF gave the phenanthridinone derivatives **5a**–**l** in good yield. N-PMB protected benzamides **5b**, **5e**, **5h**, and **5k** were treated with TFA to afford the phenanthridinone derivatives 6a-6d, while N-MOM protected benzamides **5c**, **5f**, and **5i** were treated with LiAlH_4_ to provide planar phenanthridine derivatives **7a**–**c**.

The route to the desired N-5 substituted derivatives was illustrated in Scheme 2. We prepared N-5 carboxyl substituted derivatives **9a**, **9b**, **12a**, and **12b** by initially forming compounds **8a**, **8b**, **11a**, and **11b**, followed by hydrolysis of the ester moiety. Treatment of carboxyl derivatives with dimethylamine provided **10a**, **10b**, **13a**, and **13b**.

The general synthetic methodology for the synthesis of C-6 substituted derivatives was outlined in Scheme 3. **6a**, **6b**, and **6d** were treated with POCl_3_ to afford the C-6 chlorinated analogues of **14a**–**c**, followed by substitution reaction with *N*,*N*-dimethylethylenediamine to provide derivatives **15a**–**c**. Compounds **7a** and **7c** underwent N-methylation with iodomethane to form phenanthridinium salts **16a** and **16b**.

### 2.2. Evaluation of Antitumor Activity

The antitumor activities of the derivatives were evaluated against the human cancer cell lines including HepG2, A549, NCI-H460, and CNE1 in vitro by using the 3-(4,5-dimethylthiazol-2-yl)-2,5-diphenyltetrazolium bromide (MTT) assay. Nitidine chloride was also presented as the comparative criterion. The results were summarized in Table 1. Derivatives which did not demonstrate obvious activity were not presented in the table.

It was found that most of target compounds showed moderate to high antitumor activity, with the IC_50_ value range from 1.13 μM to 48.79 μM. Compounds **5e**, **15a**, **15b**, **15c**, and **16a** exhibited good inhibition against all of the test cell line.

For HepG2 cells, compounds **5e**, **15a**, **15b**, and **15c** displayed high activity with the IC_50_ values of 9.07 μM, 1.68 μM, 1.19 μM, and 2.15 μM, respectively. Among them, compound **15b** had the best inhibition and was superior to nitidine chloride. Similar results for A549 cell and NCI-H460 cell were gave, and compound **15c** had the highest activity with IC_50_ of 1.37 μM against A549 cell and 5.24 μM against NCI-H460 cell, which were better than or close to nitidine chloride.

For CNE1 cells, N-5 substituted phenanthridinone derivatives **5a**, **5b**, **5c**, and **5e** showed moderate to high activity than phenanthridinones **6a**–**d**, revealing that the introduction of substituent in the N-5 position could significantly improve the antitumor activity. In particular, compound **5e** exhibited the highest cytotoxicity against CNE1 cell with the IC_50_ value of 1.13 μM and was 1.6-fold more active than nitidine chloride (IC_50_: 1.85 μM). However, derivatives including **8**–**13** with a long chain in N-5 position did not demonstrate obvious activity. In addition, all of the planar derivatives (except **7a** and **14a**–**c**) exhibited high cytotoxicity with the IC_50_ values range from 1.19 μM to 15.17 μM.

It should be noted that planar conjugated structure, especially with substituent in the C-6 position, enhanced the antitumor activity. The moiety of ammonium salt was less important than the benzo[*c*]phenanthridine alkaloids. Moreover, short chain in the N-5 position of the phenanthridinone derivatives improved the antitumor activity, while the presence of long alkyl chain may decrease the activity.

## 3. Materials and Methods

### 3.1. General Information

All commercial reagents and solvents were used as received without further purification unless otherwise indicated. Synthetic compounds **3a**–**d** and **4a**–**l** were directly used for reaction without further purification and characterization. Melting points were recorded on an SGW X-4 microscope melting point apparatus (Shanghai Tech Instrument Co., Ltd., Shanghai, China). Infrared spectra (IR) were performed on NICOLET iS10 sepectrometer (Shimazu Co., Ltd., Kyoto, Japan). NMR spectra were recorded on a Bruker Avance 500MHz spectrometer (Bruker Co., Ltd., Zurich, Switzerland) at room temperature with tetramethylsilane (TMS) as an internal standard and CDCl_3_ or DMSO-d_6_ as solvents. Mass spectra (MS) were obtained by LCMS-IT-TOF spectrometer (Shimadzu Co., Ltd., Kyoto, Japan) or TSQ Quantum Ultra (Thermo Scientific Co., Ltd., Madison, WI, USA). Elemental analysis for C, H, O, and N were carried out with Elementar VarioMICRO Cube analyzer (Elementar, Frankfurt, Germany).

### 3.2. General Procedure for the Synthesis of Compounds ***5a**–**l***

2-bromo-4,5-dimethoxy-*N*-(3-methoxyphenyl)-*N*-methylbenzamide (**4a**) (2.00 g, 5.26 mmol), Pd(oAc)_2_ (0.118 g, 0.526 mmol), P(*o*-tol)_3_ (0.32 g, 1.052 mmol), K_2_CO_3_ (2.90 g, 21.0 mmol), and dry DMF (20 mL) were mixed under nitrogen atmosphere, and the mixture was stirred at 100 °C overnight. After cooled to room temperature, the reaction mixture was extracted with CH_2_Cl_2_. The organic phase was washed with H_2_O and brine, and then dried over anhydrous Na_2_SO_4_. After being concentrated under reduced pressure, the residue was purified on silica gel column chromatography (PE/EA, *v*/*v* = 1:1) to obtain compound **5a**. Same method was used to provide compounds **5b**–**l** from **4b**–**l**, respectively. Derivatives **5b**, **5c**, **5e**, **5f**, **5h**, **5i**, **5k**, and **5l** were used without purification and further characterization.

*3,8,9-trimethoxy-5-methyl-5H-phenanthridin-6-one* (**5a**) White solid, yield 87%, m.p. 191.0–193.0 °C. FTIR (KBr, cm^−1^) 3441, 3132, 1643, 1608, 1510, 1463, 1402, 1313, 1262, 1230, 1146, 1032, 872, 821, 775, 726. ^1^H-NMR (CDCl_3_) δ 8.06 (d, *J* = 8.6 Hz, 1H), 7.89 (s, 1H), 7.48 (s, 1H), 6.91–6.87 (m, 2H), 4.08 (s, 3H), 4.02 (s, 3H), 3.94 (s, 3H), 3.78 (s, 3H). ^13^C-NMR (CDCl_3_) δ 160.3, 153.5, 149.2, 139.2, 128.8, 128.4, 124.1, 113.0, 109.3, 109.0, 105.5, 102.2, 100.4, 56.3, 56.2, 55.7, 30.1. ESI-MS *m*/*z*: 338.34 ([M + K]^+^). Anal. Calcd for C_17_H_17_NO_4_: C 68.21; H 5.72; N 4.68; O 21.68. Found: C 67.92; H 5.58; N 4.55; O 21.40 (See Supplementary Materials).

*8,9-dimethoxy-5-methyl-2,3,4,5-tetrahydro-1H-benzo[c]phenanthridin-6-one* (**5d**) White solid, yield 90%, m.p. 167.0–169.0 °C. FTIR (KBr, cm^−1^) 3441, 3132, 2931, 1640, 1604, 1516, 1493, 1456, 1419, 1374, 1312, 1271, 1234, 1212, 1153, 1028, 907, 768. ^1^H-NMR (CDCl_3_) δ 7.85–7.87 (m, 2H), 7.51 (s, 1H), 7.06 (d, *J* = 8.2 Hz, 1H), 4.06 (s, 3H), 4.02 (s, 3H), 3.74 (s, 3H), 3.60 (s, 1H), 3.33 (s, 1H), 2.95 (t, *J* = 6.3 Hz, 4H), 1.86 (m, 2H), 1.68 (m, 2H). ^13^C-NMR (CDCl_3_) δ 153.4, 149.5, 139.7, 139.2, 129.1, 127.7, 124.7, 119.6, 118.6, 108.8, 102.7, 56.3, 56.2, 39.7, 30.3, 29.6, 23.4, 22.3. ESI-MS: *m*/*z*: 324.16 ([M + H]^+^). Anal. Calcd for C_20_H_21_NO_3_: C 74.28; H 6.55; N 4.33; O 14.84. Found: C 74.03; H 6.68; N 4.24; O 14.92.

*2-fluoro-8,9-dimethoxy-5-methyl-5H-phenanthridin-6-one* (**5g**) White solid, yield 87%, m.p. 215.0–216.0 °C. FTIR (KBr, cm^−1^) 3433, 3134, 1640, 1594, 1515, 1465, 1403, 1320, 1277, 1187, 1027, 856, 808, 780, 616. ^1^H-NMR (CDCl_3_) δ 7.94 (s, 1H), 7.82–7.79 (dd, *J* = 2.7, 6.9 Hz, 1H), 7.47 (s, 1H), 7.38–7.35 (dd, *J* = 4.4, 4.7 Hz, 1H), 7.25-7.21 (m, 1H), 4.09 (s, 3H), 4.04 (s, 3H), 3.81 (s, 3H). ^13^C-NMR (CDCl_3_) δ 160.8, 158.5 (d, *J* = 241.1 Hz), 153.3, 150.3, 134.0, 127.3, 120.5 (d, *J* = 7.7 Hz), 120.0, 116.5 (d, *J* = 9.0 Hz), 115.8 (d, *J* = 23.0 Hz), 109.2, 108.5 (d, *J* = 23.5 Hz), 102.7, 56.3, 56.2, 30.2. ESI-MS *m*/*z*: 288.12 ([M + H]^+^). Anal. Calcd for C_16_H_14_FNO_3_: C 66.89; H 4.91; N 4.88; O 16.71. Found: C 66.61; H 4.73; N 4.55; O 16.45.

*8,9-dimethoxy-5-methyl-6-oxo-5,6-dihydro-phenanthridine-2-carboxylic acid methyl ester* (**5j**) White solid, yield 84%, m.p. 217.6–218.6 °C. FTIR (KBr, cm^−1^) 3435, 3134, 1711, 1648, 1613, 1516, 1402, 1314, 1267, 1112, 1032. ^1^H-NMR (CDCl3) δ 8.84 (d, *J* = 1.9 Hz, 1H), 8.16-8.14 (dd, *J* = 1.9, 1.9 Hz, 1H), 7.93 (s, 1H), 7.67 (s, 1H), 7.44 (d, *J* = 8.9 Hz, 1H), 4.14 (s, 3H), 4.05 (s, 3H), 4.00 (s, 3H), 3.84 (s, 3H). ^13^C-NMR (CDCl_3_) δ 166.9, 161.4, 153.8, 150.5, 140.9, 129.6, 128.0, 124.8, 124.0, 119.8, 119.1, 115.1, 109.3, 102.9, 56.5, 56.4, 52.4, 30.4. ESI-MS *m*/*z*: 318.12 ([M + H]^+^). Anal. Calcd for C_18_H_17_NO_5_: C 66.05; H 5.23; N 4.28; O 24.44. Found: C 66.21; H 5.14; N 3.96; O 24.16.

### 3.3. General Procedure for the Synthesis of Compounds ***6a**–**d***

TFA (6 mL) was slowly added to the flask with **5b** (1.70 g, 4.193 mmol) under nitrogen atmosphere at 75 °C. The mixture was stirred overnight. After cooling, the reaction mixture was quenched with ethyl acetate and water, and then extracted with ethyl acetate. The organic phase was washed with H_2_O, aqueous NaHCO_3_ and brine, and dried over anhydrous Na_2_SO_4_. The crude product was purified on silica gel column chromatography (DCM/EA, *v*/*v* = 1:1) to obtain compound **6a**. The same method was used for **6b**–**d** from reactants **5e**, **5h**, and **5k**.

*3,8,9-trimethoxy-5H-phenanthridin-6-one* (**6a**) White solid, yield 69%, m.p. 267.4–269.1 °C. FTIR (KBr, cm^−1^) 3440, 3160, 1662, 1612, 1503, 1404, 1330, 1258, 1210, 1176, 1098, 1044, 877, 840, 802. ^1^H-NMR (CDCl_3_) δ 9.80 (br, 1H), 7.99 (d, *J* = 8.9 Hz, 1H), 7.51 (s, 1H), 6.89 (d, *J* = 8.9 Hz, 1H), 6.74 (s, 1H), 4.09 (s, 3H), 4.04 (s, 3H), 3.91 (s, 3H). ^13^C-NMR (DMSO-d_6_) δ 160.8, 159.7, 153.4, 148.7, 137.5, 129.5, 124.7, 117.9, 111.4, 109.9, 107.9, 103.6, 99.3, 56.1, 55.6, 55.3. ESI-MS *m*/*z*: 286.12 ([M + H]^+^). Anal. Calcd for C_16_H_15_NO_4_: C 67.36; H 5.30; N 4.91; O 22.43. Found: C 67.07; H 5.38; N 4.76; O 22.24.

*8,9-dimethoxy-2,3,4,5-tetrahydro-1H-benzo[c]phenanthridin-6-one* (**6b**) White solid, yield 76%, m.p. 253.0–255.0 °C. FTIR (KBr, cm^−1^) 3439, 3132, 1651, 1610, 1496, 1398, 1234, 1129, 1079, 836. ^1^H-NMR (CDCl_3_) δ 8.56 (br, 1H), 7.87-7.86 (m, 2H), 7.59 (s, 1H), 7.03 (d, *J* = 8.0 Hz, 1H), 4.09 (s, 3H), 4.04 (s, 3H), 2.88 (t, *J* = 6.1 Hz, 2H), 2.77 (t, *J* = 6.4 Hz, 2H), 2.01-1.96 (m, 1H), 1.87-1.83 (m, 2H). ^13^C-NMR (CDCl_3_) δ 166.7, 153.9, 149.7, 138.4, 1335, 132.1, 124.0, 121.9, 119.6, 116.0, 108.6, 103.0, 56.4, 56.3, 30.1, 23.6, 22.8, 22.5. ESI-MS *m*/*z*: 310.15 ([M + H]^+^). Anal. Calcd for C_19_H_19_NO_3_: C 73.77; H 6.19; N 4.53; O 15.52. Found: C 73.43; H 6.02; N 4.36; O 15.38.

*2-fluoro-8,9-dimethoxy-5H-phenanthridin-6-one* (**6c**) White solid; yield 54%, m.p. 192.1–193.0 °C. FTIR (KBr, cm^−1^) 3438, 3142, 1643, 1600, 1535, 1510, 1402, 1262, 1213, 1029, 824. ^1^H-NMR (CDCl_3_) δ 7.94 (br, 1H), 7.63–7.61 (m, 2H), 7.33 (s, 1H), 7.10–7.05 (m, 2H), 3.93 (s, 3H), 3.92 (s, 1H). ^13^C-NMR (CDCl_3_) δ 164.9, 160.0 (d, *J* = 243.7), 151.4, 148.8, 133.8, 129.0, 122.0, 122.0, 116.0, 116.0, 115.9, 113.4, 110.0, 56.5, 56.4. ESI-MS *m*/*z*: 274.35 ([M + H]^+^). Anal. Calcd for C_15_H_12_FNO_3_: C 65.93; H 4.43; N 5.13; O 17.57. Found: C 65.27; H 4.30; N 5.06; O 17.42.

*8,9-dimethoxy-6-oxo-5,6-dihydro-phenanthridine-2-carboxylic acid methyl ester* (**6d**) White solid, yield 78%, m.p. 296.5–299.0 °C. FTIR (KBr, cm^−1^) 3438, 3156, 1709, 1663, 1617, 1514, 1402, 1267, 1092, 1035. ^1^H-NMR (DMSO-d_6_) δ 11.93 (s, 1H), 8.84–8.82 (m, 1H), 8.00–7.97 (m, 1H), 7.84 (d, *J* = 3.0 Hz, 1H), 7.70 (s, 1H), 7.39 (d, *J* = 9.0 Hz, 1H), 4.06 (s, 3H), 3.91 (s, 3H), 3.90 (s, 3H). ^13^C-NMR (DMSO-d_6_) δ 166.1, 161.0, 153.9, 150.3, 140.0, 129.6, 128.8, 125.1, 124.0, 119.8, 117.8, 116.7, 108.3, 104.6, 56.7, 56.1, 52.6. ESI-MS *m*/*z*: 314.11 ([M + H]^+^). Anal. Calcd for C_17_H_15_NO_5_: C 65.17; H 4.83; N 4.47; O 25.53. Found: C 65.62; H 4.91; N 4.19; O 25.17.

### 3.4. General Procedure for the Synthesis of Compounds ***7a**–**c***

LiAlH_4_ (325 mg, 8.56 mmol) was added to the solution of **5c** (940 mg, 2.85 mmol) in dry THF (30 mL) under nitrogen atmosphere at 0 °C, and then the mixture was stirred for 4 h at room temperature. The progress of the reaction was monitored by TLC. The reaction mixture was cooled to room temperature, followed by extraction with DCM. The organic phase was washed with H_2_O and brine, dried over anhydrous Na_2_SO_4_, and then concentrated under reduced pressure. The crude product was purified on silica gel column chromatography (PE/EA, *v*/*v* = 1:2) to obtain compound **7a**. The same method was used for **7b** and **7c** from compounds **5f** and **5i**.

*3,8,9-trimethoxyphenanthridine* (**7a**) White solid, yield 55%, m.p. 163.0–165.0 °C. FTIR (KBr, cm^−1^) 3435, 3141, 1619, 1504, 1398, 1274, 1213, 1162, 1034, 840, 808. ^1^H-NMR (CDCl_3_) δ 9.13 (s, 1H), 8.36 (d, *J* = 8.0 Hz, 1H), 7.81 (s, 1H), 7.63 (s, 1H), 7.35 (s, 1H), 7.33–7.30 (m, 1H), 4.15 (s, 3H), 4.07 (s, 3H), 4.00 (s, 3H). ^13^C-NMR (CDCl_3_) δ 159.6, 153.3, 152.1, 149.5, 145.6, 128.8, 123.0, 121.0, 118.2, 118.1, 109.7, 107.9, 101.5, 56.3, 56.2, 55.7. ESI-MS *m*/*z*: 270.12 ([M + H]^+^). Anal. Calcd for C_16_H_15_NO_3_: C 71.36; H 5.61; N 5.20; O 17.82. Found: C 70.90; H 5.43; N 5.15; O 17.55.

*8,9-dimethoxy-1,2,3,4-tetrahydrobenzo[c]phenanthridine* (**7b**) White solid, yield 45%, m.p. 178.6–179.8 °C. FTIR (KBr, cm^−1^) 3437, 3140, 2930, 1612, 1502, 1405, 1269, 1201, 1157, 1027, 846. ^1^H-NMR (CDCl_3_) δ 9.14 (s, 1H), 8.18 (d, *J* = 8.7 Hz, 1H), 7.82 (s, 1H), 7.35 (d, *J* = 8.7 Hz, 1H), 7.31 (s, 1H), 4.12 (s, 3H), 4.06 (s, 3H), 3.40 (t, *J* = 5.3 Hz, 2H), 2.97 (t, *J* = 6.0 Hz, 2H), 1.99–1.96 (m, 2H), 1.93–1.90 (m, 2H). ^13^C-NMR (CDCl_3_) δ 152.9, 150.2, 149.7, 142.5, 136.9, 135.6, 128.8, 128.5, 121.5, 121.4, 118.8, 107.7, 101.8, 56.2, 56.2, 30.3, 25.8, 23.3, 23.1. ESI-MS *m*/*z*: 294.15 ([M + H]^+^). Anal. Calcd for C_19_H_19_NO_2_: C 77.79; H 6.53; N 4.77; O 10.91. Found: C 77.34; H 6.38; N 4.52; O 10.94.

*2-fluoro-8,9-dimethoxyphenanthridine* (**7c**) White solid, yield 47%, m.p. 178.0–179.0 °C. FTIR (KBr, cm^−1^) 3436, 3134, 1617, 1511, 1400, 1270, 1195, 1151, 1098, 1028, 848. ^1^H-NMR (CDCl_3_) δ 9.11 (s, 1H), 8.16–8.05 (m, 1H), 8.04–8.02 (m, 1H), 7.74 (s, 1H), 7.45–7.41 (m, 1H), 7.37 (s, 1H), 4.15 (s, 3H), 4.08 (s, 3H). ^13^C-NMR (CDCl_3_) δ 161.3 (d, *J* = 247.4), 153.3, 150.9 (d, *J* = 2.0), 150.7, 140.6, 132.2 (d, *J* = 9.0), 127.9 (d, *J* = 4.4), 125.3, 121.9, 117.0 (d, *J* = 24.4), 108.0, 106.6 (d, *J* = 23.2), 102.1, 56.4, 56.3, ESI-MS *m*/*z*: 258.11 ([M + H]^+^). Anal. Calcd for C_15_H_12_FNO_2_: C 70.03; H 4.70; N 5.44; O 12.44. Found: C 69.61; H 5.01; N 5.22; O 12.77.

### 3.5. General Procedure for the Synthesis of Compounds ***8a**–**c*** and ***11a**–**b***

To the solution of **6b** (216 mg, 0.70 mmol) in DMF (5 mL), Cs_2_CO_3_ (456.83 mg, 1.40 mmol), TBAI (38.84 mg, 0.105 mmol), and ethyl bromoacetate (526.83 mg, 3.15 mmol) were added, and the mixture was stirred for 1 h at 90 °C. After cooled to room temperature, the mixture was extracted with ethyl acetate. The organic phase was washed with H_2_O and brine, dried over anhydrous Na_2_SO_4_, and then concentrated under reduced pressure. The residue was purified on silica gel column chromatography (PE/EA, *v*/*v* = 4:1) to obtain compound **8a**. Same method was used for compounds **8b**, **8c** from **6c** and **6d**. Ethyl 4-bromobutyrate was used instead of ethyl bromoacetate to **11a**, **11b** from **6b** and **6c**.

*(8,9-dimethoxy-6-oxo-1,3,4,6-tetrahydro-2H-benzo[c]phenanthridin-5-yl)-acetic acid ethyl ester* (**8a**) White solid, yield 87.%, m.p. 152.3–153.7 °C. FTIR (KBr, cm^−1^) 3440, 3132, 2927, 1753, 1599, 1525, 1401, 1333, 1266, 1203, 1175, 1125, 1087, 1042, 860, 767. ^1^H-NMR (CDCl_3_) δ 8.05 (d, *J* = 8.2 Hz, 1H), 7.77 (s, 1H), 7.73 (s, 1H), 7.19 (d, *J* = 8.2 Hz, 1H), 5.10 (s, 2H), 4.25 (q, *J* = 7.1 Hz, 2H), 4.11 (s, 3H), 4.06 (s, 3H), 3.16 (t, *J* = 6.1 Hz, 2H), 2.92 (t, *J* = 5.9 Hz, 2H), 1.93–1.86 (m, 4H), 1.28 (t, *J* = 6.3 Hz, 3H). ^13^C-NMR (CDCl_3_) δ 169.7, 155.8, 152.8, 149.4, 140.5, 137.1, 134.1, 131.3, 126.0, 120.1, 118.6, 113.2, 104.8, 102.4, 63.1, 61.0, 56.2, 56.2, 30.2, 25.2, 23.4, 23.3, 14.4. ESI-MS *m*/*z*: 396.17 ([M + H]^+^). Anal. Calcd for C_23_H_25_NO_5_: C 69.86; H 6.37; N 3.54; O 20.23. Found: C 69.71; H 6.20; N 3.42; O 20.89.

*(2-fluoro-8,9-dimethoxy-6-oxo-6H-phenanthridin-5-yl)-acetic acid ethyl ester* (**8b**) White solid, yield 95%, m.p. 152.3–153.7 °C. FTIR (KBr, cm^−1^) 3439, 3128, 1744, 1659, 1600, 1509, 1399, 1258, 1208, 1024, 845. ^1^H-NMR (CDCl_3_) δ 7.28–7.25 (m, 1H), 6.90–6.87 (m, 2H), 6.84 (s, 1H), 6.65 (s, 1H), 4.55 (s, 2H), 4.25 (q, *J* = 7.2 Hz, 2H), 3.80 (s, 3H), 3.71 (s, 3H), 1.31 (t, *J* = 7.1 Hz, 3H). ^13^C-NMR (CDCl_3_) δ 169.1, 169.0, 161.6 (d, *J* = 240 Hz), 150.0, 148.0, 138.7, 138.6, 129.4, 129.4, 116.1, 116.0, 115.4, 112.0, 110.6, 61.7, 56.2, 56.2, 51.5, 29.8, 14.3. ESI-MS *m*/*z*: 360.09 ([M + H]^+^). Anal. Calcd for C_19_H_18_FNO_5_: C 63.50; H 5.05; N 3.90; O 22.26. Found: C 63.04; H 4.85; N 3.62; O 21.10.

*5-ethoxycarbonylmethyl-8,9-dimethoxy-6-oxo-5,6-dihydro-phenanthridine-2-carboxylic acid methyl ester* (**8c**) White solid, yield 87%, m.p. 211.9–214.0 °C. FTIR (KBr, cm^−1^) 3432, 3134, 1740, 1711, 1648, 1614, 1517, 1400, 1322, 1270, 1215, 1118, 1046, 1017, 835, 769. ^1^H-NMR (CDCl_3_) δ 8.85 (s, 1H), 8.10 (d, *J* = 8.9 Hz, 1H), 7.91 (s, 1H), 7.67 (s, 1H), 7.17 (d, *J* = 8.9 Hz, 1H), 5.21 (s, 2H), 4.25 (q, *J* = 7.2 Hz, 2H), 4.14 (s, 3H), 4.04 (s, 3H), 3.99 (s, 3H), 1.27 (t, *J* = 7.2 Hz, 4H). ^13^C-NMR (CDCl_3_) δ 168.3, 166.7, 154.2, 150.5, 140.1, 129.7, 128.4, 125.2, 124.4, 119.3, 119.2, 114.6, 109.4, 103.1, 62.0, 56.6, 56.4, 52.4, 44.7, 14.3. ESI-MS *m*/*z*: 400.13 ([M + H]^+^). Anal. Calcd for C_21_H_21_NO_7_: C 63.15; H 5.30; N 3.51; O 28.04. Found: C 63.27; H 5.41; N 3.49; O 27.86.

*4-(8,9-dimethoxy-6-oxo-1,3,4,6-tetrahydro-2H-benzo[c]phenanthridin-5-yl)-butyric acid ethyl ester* (**11a**) White solid, yield 76%, m.p. 104.8–106.8 °C. FTIR (KBr, cm^−1^) 3434, 3137, 2921, 1728, 1595, 1528, 1403, 1322, 1267, 1228, 1176, 1035, 859, 766. ^1^H-NMR (CDCl_3_) δ 8.05 (d, *J* = 8.4 Hz, 1H), 7.78 (s, 1H), 7.64 (s, 1H), 7.18 (d, *J* = 8.4 Hz, 1H), 4.70 (t, *J* = 6.2 Hz, 2H), 4.14–4.10 (m, 5H), 4.06 (s, 3H), 3.25 (t, *J* = 6.1 Hz, 2H), 2.93 (t, *J* = 6.1 Hz, 2H), 2.58 (t, *J* = 7.4 Hz, 2H), 2.33–2.27 (m, 2H), 1.95–1.87 (m, 4H), 1.22 (t, *J* = 7.6 Hz, 3H). ^13^C-NMR (CDCl_3_) δ 173.6, 156.9, 152.6, 149.3, 141.1, 137.0, 134.1, 131.0, 125.6, 119.7, 118.5, 113.8, 104.7, 102.4, 65.0, 60.5, 56.2, 56.2, 31.7, 30.3, 25.4, 24.8, 23.5, 23.3, 14.3. ESI-MS *m*/*z*: 424.20 ([M + H]^+^). Anal. Calcd for C_25_H_29_NO_5_: C 70.90; H 6.90; N 3.31; O 18.89. Found: C 70.82; H 6.95; N 3.38; O 18.77.

*4-(2-fluoro-8,9-dimethoxy-6-oxo-6H-phenanthridin-5-yl)-butyric acid ethyl ester* (**11b**) Yellow oil, yield 79.%. FTIR (KBr, cm^−1^) 3437, 3120, 2934, 1729, 1643, 1598, 1508, 1444, 1405, 1323, 1259, 1210, 1184, 1092, 1023, 848, 787. ^1^H-NMR (CDCl_3_) δ 7.15–7.12 (m, 2H), 6.91–6.87 (m, 1H), 6.81 (s, 1H), 6.54 (s, 1H), 4.12 (q, *J* = 7.1 Hz, 2H), 3.94 (t, *J* = 6.7 Hz, 1H), 3.78 (s, 3H), 3.71 (s, 3H), 2.45 (t, *J* = 6.8 Hz, 1H), 2.00–1.94 (m, 2H), 1.24 (t, *J* = 7.1 Hz, 3H). ^13^C-NMR (CDCl_3_) δ 173.1, 168.7, 161.4 (d, *J* = 248.0), 149.7, 148.0, 138.0, 138.0, 129.5, 129.4, 116.2, 116.0, 115.3, 111.7, 110.5, 60.6, 56.2, 56.2, 489, 31.7, 23.1, 14.3. ESI-MS *m*/*z*: 388.12 ([M + H]^+^). Anal. Calcd for C_21_H_22_FNO_5_: C 65.11; H 5.72; N 3.62; O 20.65. Found: C 64.94; H 5.63; N 3.65; O 20.32.

### 3.6. General Procedure for the Synthesis of Compounds ***9a**–**b*** and ***12a**–**b***

To the solution of 8a (107 mg, 0.27 mmol) in MeOH (27 mL) was added the solution of 10N NaOH (18 mL). The reaction mixture was refluxed for 16 h at room temperature. And then 2 M HCl was added to adjust pH~2. The aqueous layer was extracted with ethyl acetate. The combined organic phase was washed with H_2_O and brine, dried over anhydrous Na_2_SO_4_, and then concentrated under reduced pressure. The residue was purified on silica gel column chromatography (DCM/MeOH, *v*/*v* = 15:1) to obtain compound **9a**. The same method was used for **9b**, **12a**, and **12b** from reactants **8b**, **11a**, and **11b**.

*(8,9-dimethoxy-6-oxo-1,3,4,6-tetrahydro-2H-benzo[c]phenanthridin-5-yl)-acetic acid* (**9a**) White solid, yield 93%, m.p. 181.3–182.3 °C. FTIR (KBr, cm^−1^) 3130, 2932, 1724, 1599, 1402, 1330, 1261, 1211, 1173, 1127, 1036. ^1^H-NMR (CDCl_3_) δ 8.09 (d, *J* = 8.4 Hz, 1H), 7.80 (s, 1H), 7.68 (s, 1H), 7.27 (d, *J* = 8.4, 1H), 5.18 (s, 2H), 4.13 (s, 3H), 4.06 (s, 3H), 3.17 (t, *J* = 6.0 Hz, 2H), 2.94 (t, *J* = 6.0 Hz, 2H), 1.95–1.86 (m, 4H). ^13^C-NMR (CDCl_3_) δ 157.1, 153.4, 149.7, 137.8, 133.6, 131.7, 126.7, 120.3, 118.7, 112.9, 104.5, 102.4, 65.4, 56.3, 56.3, 30.3, 29.9, 25.2, 23.2, 23.1. ESI-MS *m*/*z*: 368.14 ([M + H]^+^). Anal. Calcd for C_21_H_21_NO_5_: C 68.65; H 5.76; N 3.81; O 21.77. Found: C 68.50; H 5.34; N 3.73; O 21.52.

*(2-fluoro-8,9-dimethoxy-6-oxo-6H-phenanthridin-5-yl)-acetic acid* (**9b**) White solid, yield 96%, m.p. 107.0–108.8 °C. FTIR (KBr, cm^−1^) 3439, 3132, 1638, 1509, 1400, 1258, 1212, 1014, 844. ^1^H-NMR (CDCl_3_) δ 7.25 (m, 1H), 6.90–6.88 (m, 2H), 6.84 (s, 1H), 6.65 (s, 1H), 4.61 (s, 2H), 3.80 (s, 3H), 3.72 (s, 3H). ^13^C-NMR (CDCl_3_) δ 169.7, 162.5, 160.5, 150.0, 148.0, 138.6, 138.6, 129.3, 129.2, 116.1, 115.9, 115.2, 112.1, 110.4, 56.3, 56.1, 29.8. ESI-MS *m*/*z*: 332.12 ([M + H]^+^). Anal. Calcd for C_17_H_14_FNO_5_: C 61.63; H 4.26; N 4.23; O 24.15. Found: C 61.42; H 4.55; N 4.05; O 23.80.

*4-(8,9-dimethoxy-6-oxo-1,3,4,6-tetrahydro-2H-benzo[c]phenanthridin-5-yl)-butyric acid* (**12a**) White solid, yield 95%, m.p. 209.0–210.0 °C. FTIR (KBr, cm^−1^) 3443, 3130, 2931, 1703, 1594, 1503, 1401, 1325, 1270, 1208, 1040, 1001, 864, 774. ^1^H-NMR (DMSO-d_6_) δ 8.33 (d, *J* = 8.4 Hz, 1H), 8.01 (s, 1H), 7.58 (s, 1H), 7.19 (d, *J* = 8.4 Hz, 1H), 4.59 (t, *J* = 7.1 Hz, 2H), 4.03 (s, 3H), 3.93 (s, 3H), 3.16 (t, *J* = 7.7 Hz, 2H), 2.88 (t, *J* = 4.8 Hz, 2H), 2.48 (t, *J* = 6.7 Hz, 2H), 2.16–2.11 (m, 2H), 1.87-1.81 (m, 4H). ^13^C-NMR (DMSO-d_6_) δ 174.7, 156.8, 153.2, 149.6, 140.7, 136.8, 133.2, 130.8, 125.8, 119.9, 119.7, 113.1, 104.5, 103.5, 65.3, 56.4, 56.1, 31.3, 29.9, 25.2, 24.6, 23.3, 23.2. ESI-MS *m*/*z*: 396.18 ([M + H]^+^). Anal. Calcd for C_23_H_25_NO_5_: C 69.86; H 6.37; N 3.54; O 20.23. Found: C 69.88; H 6.46; N 3.29; O 20.51.

*4-(2-fluoro-8,9-dimethoxy-6-oxo-6H-phenanthridin-5-yl)-butyric acid* (**12b**) White solid, yield 98. %, m.p. 47.5–49.6 °C. FTIR (KBr, cm^−1^) 3440, 3132, 1644, 1509, 1400, 1257, 1213, 1024, 841. ^1^H-NMR (CDCl_3_) δ 7.14 (dd, *J* = 8.7, 4.8 Hz, 2H), 6.91 (t, *J* = 8.4 Hz, 1H), 6.82 (s, 1H), 6.53 (s, 1H), 3.97 (d, *J* = 6.3 Hz, 2H), 3.79 (s, 3H), 3.70 (s, 3H), 2.54 (t, *J* = 7.3 Hz, 2H), 2.00–1.95 (m, 2H). ^13^C-NMR (CDCl_3_) δ 176.7, 169.2, 161.5 (d, *J* = 251.0 Hz), 149.9, 148.1, 137.8, 130.2, 129.4 (d, *J* = 8.7 Hz), 116.2 (d, *J* = 22 Hz), 115.3, 111.6, 110.6, 100.1, 56.3, 56.2, 48.8, 31.3, 23.0. ESI-MS *m*/*z*: 360.13 ([M + H]^+^). Anal. Calcd for C_19_H_18_FNO_5_: C 63.50; H 5.05; N 3.90; O 22.26. Found: C 63.24; H 4.92; N 3.68; O 22.01.

### 3.7. General Procedure for the Synthesis of Compounds ***10a**–**b*** and ***13a**–**b***

Compound **9a** (36.8 mg, 0.1 mmol) was dissolved in THF (10 mL) and CH_2_Cl_2_ (4 mL) at 0 °C. Triethylamine (20 μL) and isobutyl chloroformate (18 μL) were added (18 μL), and the mixture was stirred for 20 min. Then dimethylamine (0.5 mL) was added at 0 °C and the mixture was stirred for 30 min at room temperature. After evaporation of solvent under reduced pressure, the mixture was extracted with ethyl acetate. The combined organic phase was washed with H_2_O and brine, dried over anhydrous Na_2_SO_4_, and then concentrated under reduced pressure. The crude product was purified on silica gel column chromatography (DCM/MeOH, *v*/*v* = 30:1) to obtain compound **10a**. The same method was used for **10b**, **13a**, and **13b** from **9b**, **12a**, and **12b**.

*2-(8,9-dmethoxy-6-oxo-1,3,4,6-tetrahydro-2H-benzo[c]phenanthridin-5-yl)-N,N-dimethyl-acetamide* (**10a**) White solid, yield 85%, m.p. 196.0–197.8 °C. FTIR (KBr, cm^−1^) 3435, 3134, 2931, 1667, 1601, 1502, 1400, 1324, 1269, 1215, 1175, 1122, 1035, 769. ^1^H-NMR (CDCl_3_) δ 8.05 (d, *J* = 8.4 Hz, 1H), 7.78 (s,1H), 7.77 (s, 1H), 7.19 (d, *J* = 8.4 Hz, 1H), 5.27 (s, 2H), 4.11 (s, 3H), 4.06 (s, 3H), 3.19 (s, 3H), 3.18 (t, *J* = 6.5 Hz, 2H), 3.04 (s, 3H), 2.92 (t, *J* = 6.0 Hz, 2H), 1.94–1.85 (m, 4H). ^13^C-NMR (CDCl_3_) δ 168.6, 156.0, 152.8, 149.4, 140.7, 137.0, 133.9, 131.3, 125.9, 120.1, 118.6, 113.4, 104.8, 102.4, 63.4, 56.3, 56.2, 36.6, 35.8, 30.2, 25.2, 23.5, 23.2. ESI-MS *m*/*z*: 395.19 ([M + H]^+^). Anal. Calcd for C_23_H_26_N_2_O_5_: C 70.03; H 6.64; N 7.10; O 16.22. Found: C 69.71; H 6.55; N 6.96; O 16.19.

*2-(2-fluoro-8,9-dimethoxy-6-oxo-6H-phenanthridin-5-yl)-N,N-dimethyl-acetamide* (**10b**) White solid; yield 83%, m.p. 57.0–59.0 °C. FTIR (KBr, cm^−1^) 3442, 3123, 2934, 1652, 1600, 1508, 1399, 1325, 1257, 1212, 1155, 1032, 927, 847, 790. ^1^H-NMR (CDCl_3_) δ 7.37–7.34 (m, 2H), 6.87–6.82 (m, *2*H), 6.75 (s, 1H), 4.63 (s, 2H), 3.79 (s, 3H), 3.71 (s, 3H), 3.07 (s, 3H), 3.02 (s, 3H). ^13^C-NMR (CDCl_3_) δ 169.1, 167.4, 149.9, 148.1, 130.0, 129.6, 129.6, 115.9, 115.7, 115.2, 114.4, 112.3, 110.4, 108.4, 56.3, 56.2, 51.5, 36.6, 36.1. ESI-MS *m*/*z*: 361.17 ([M + H]^+^). Anal. Calcd for C_19_H_19_FN_2_O_4_: C 63.68; H 5.34; N 7.82; O 17.86. Found: C 63.27; H 5.09; N 7.72; O 17.48.

*4-(8,9-dimethoxy-6-oxo-1,3,4,6-tetrahydro-2H-benzo[c]phenanthridin-5-yl)-N,N-dimethyl-butyramide* (**13a**) White solid, yield 84.%, m.p. 249.2–251.5 °C. FTIR (KBr, cm^−1^) 3436, 3135, 2924, 1643, 1597, 1501, 1403, 1320, 1264, 1229, 1205, 1174, 1034, 996, 868, 776. ^1^H-NMR (CDCl_3_) δ 8.05 (d, *J* = 8.3 Hz, 1H), 7.78 (s, 1H), 7.65 (s, 1H), 7.18 (d, *J* = 8.3 Hz, 1H), 4.70 (t, *J* = 6.2 Hz, 2H), 4.11 (s, 3H), 4.05 (s, 3H), 3.25 (t, *J* = 5.9 Hz, 2H), 2.99 (s, 3H), 2.96 (s, 3H), 2.58 (t, *J* = 7.6 Hz, 2H), 2.58 (t, *J* = 7.5 Hz, 2H), 2.35-2.29 (m, 2H), 1.94-1.88 (m, 4H). ^13^C-NMR (CDCl_3_) δ 172.7, 156.9, 152.5, 149.2, 141.2, 136.9, 134.1, 131.0, 125.5, 119.6, 118.5, 113.8, 104.6, 102.4, 66.3, 56.2, 56.1, 37.3, 35.6, 30.3, 30.2, 25.4, 25.0, 23.4, 23.3. ESI-MS *m*/*z*: 423.22 ([M + H]^+^). Anal. Calcd for C_25_H_30_N_2_O_4_: C 71.07; H 7.16; N 6.63; O 15.15. Found: C 70.82; H 7.22; N 6.37; O 15.31.

*4-(2-fluoro-8,9-dimethoxy-6-oxo-6H-phenanthridin-5-yl)-N,N-dimethyl-butyramide* (**13b**) White solid, yield 82.%, m.p. 48.9–50.5 °C. FTIR (KBr, cm^−1^) 3437, 3132, 1646, 1508, 1400, 1258, 1211, 1024, 846. ^1^H-NMR (CDCl_3_) δ 7.18–7.05 (m, 1H), 6.88 (t, *J* = 8.5 Hz, 1H), 6.81 (s, 1H), 6.54 (s, 1H), 3.95 (t, *J* = 6.9 Hz, 2H), 3.78 (s, 3H), 3.69 (s, 3H), 3.02(s, 3H), 2.94 (s, 3H), 2.49 (t, *J* = 7.3 Hz, 2H), 2.02-1.97 (m, 2H). ^13^C-NMR (CDCl_3_) δ 172.3, 168.8, 149.7, 148.0, 138.2, 130.6, 129.6, 129.5, 116.2, 116.0, 115.3, 111.8, 110.5, 100.1, 56.2, 49.3, 37.4, 35.6, 30.8, 23.4. ESI-MS *m*/*z*: 387.16 ([M + H]^+^). Anal. Calcd for C_21_H_23_FN_2_O_4_: C 65.27; H 6.00; N 7.25; O 16.56. Found: C 64.92; H 5.97; N 6.97; O 16.37.

### 3.8. General Procedure for the Synthesis of Compounds ***14a**–**c***

POCl_3_ (0.64 mL) was added to compound 6a (100 mg, 0.35 mmol) in flask. Then the reaction mixture was stirred for 2 h at 105 °C. After cooled to room temperature, the mixture was poured carefully into a beaker filled with ice water. Concentrated ammonia water was added untill pH > 7. The precipitation was washed with water and purified on silica gel column chromatography (PE/DCM, *v*/*v* = 1:1) to obtain compound **14a**. The same method was used for **14b** and **14c** from compounds **6b** and **6d.**

*6-chloro-3,8,9-trimethoxyphenanthridine* (**14a**) White solid, yield 83%, m.p. 174.1–175.6 °C. FTIR (KBr, cm^−1^) 3443, 3133, 1617, 1579, 1503, 1400, 1313, 1234, 1213, 1155, 1113, 1038, 912, 845. ^1^H-NMR (CDCl_3_) δ 8.29 (d, *J* = 9.1 Hz, 1H), 7.77 (s, 1H), 7.72 (s, 1H), 7.47 (d, *J* = 2.6 Hz, 1H), 7.29 (d, *J* = 2.6 Hz), 4.14 (s, 3H), 4.09 (s, 3H), 3.96 (s, 3H). ^13^C-NMR (CDCl_3_) δ 160.1, 153.8, 150.4, 149.8, 144.5, 130.9, 123.0, 119.0, 118.5, 118.0, 109.0, 107.1, 101.8, 56.4, 56.3, 55.8. ESI-MS *m*/*z*: 304.08 ([M + H]^+^). Anal. Calcd for C_16_H_14_ClNO_3_: C 63.27; H 4.65; N 4.61; O 15.80. Found: C 63.41; H 4.80; N 4.39; O 16.26.

*6-chloro-8,9-dimethoxy-1,2,3,4-tetrahydrobenzo[c]phenanthridine* (**14b**) White solid, yield 85%, m.p. 201.3–202.3 °C. FTIR (KBr, cm^−1^) 3434, 3133, 2928, 1613, 1578, 1523, 1501, 1465, 1402, 1299, 1249, 1206, 1160, 1081, 1043, 953, 840. ^1^H-NMR (CDCl_3_) δ 8.13 (d, *J* = 8.4 Hz, 1H), 7.83 (s, 1H), 7.72 (s, 1H), 7.34 (d, *J* = 8.4 Hz, 1H), 4.14 (s, 3H), 4.09 (s, 3H), 3.35 (t, *J* = 6.1 Hz, 2H), 2.96 (t, *J* = 6.0 Hz, 2H), 1.96-1.89 (m, 4H)). ^13^C-NMR (CDCl_3_) δ 153.2, 149.0, 148.5, 141.9, 137.6, 135.3, 130.9, 128.7, 121.5, 119.6, 118.6, 106.9, 102.2, 56.3, 56.3, 30.2, 25.3, 23.1, 23.1. ESI-MS *m*/*z*: 328.11 ([M + H]^+^). Anal. Calcd for C_19_H_18_ClNO_2_: C 69.62; H 5.53; N 4.27; O 9.76. Found: C 69.62; H 5.48; N 4.17; O 9.45.

*9-chloro-6,7-dimethoxy-phenanthrene-3-carboxylic acid methyl ester* (**14c**) White solid, yield 83.%, m.p. 194.9–196.6 °C. FTIR (KBr, cm^−1^) 3444, 3135, 1716, 1615, 1514, 1400, 1254, 1163, 1101, 1037, 849. ^1^H-NMR (CDCl_3_) δ 9.08 (d, *J* = 1.4 Hz, 1H), 8.26 (dd, *J* = 8.6, 1.7 Hz, 1H), 8.06 (d, *J* = 8.6 Hz, 1H), 7.91 (s, 1H), 7.74 (s, 1H), 4.19 (s, 3H), 4.10 (s, 3H), 4.03 (s, 3H). ^13^C-NMR (CDCl_3_) δ 167.0, 154.1, 152.4, 150.9, 145.5, 130.6, 129.6, 128.5, 128.3, 124.6, 123.4, 120.4, 107.3, 102.5, 56.7, 56.4, 52.7. ESI-MS *m*/*z*: 332.07 ([M + H]^+^). Anal. Calcd for C_17_H_14_ClNO_4_: C 61.55; H 4.25; N 4.22; O 19.29. Found: C 61.76; H 4.72; N 4.02; O 19.77.

### 3.9. General Procedure for the Synthesis of Compounds ***15a**–**c***

To compound **14a** (60 mg, 0.20 mmol) was added *N*,*N*-dimethylaminoethylamine (696.5 mg, 7.90 mmol) under nitrogen atmosphere, and the reaction mixture was stirred at 105 °C for 6 h. After cooled to room temperature, the solvent was removed under reduced pressure and extracted with CH_2_Cl_2_. The organic phase was washed with 5% NaOH and H_2_O, dried over anhydrous Na_2_SO_4_, and then concentrated. The crude product was purified on silica gel column chromatography (DCM/MeOH, *v*/*v* = 5:1) to obtain compound **15a**. The same method was use for **15b** and **15c** from **14b** and **14c**.

*N,N-dimethyl-N’-(3,8,9-trimethoxy-phenanthridin-6-yl)-ethane-1,2-diamine* (**15a**) Yellow solid, yield 59.%, m.p. 126.0–128.0 °C. FTIR (KBr, cm^−1^) 3414, 3138, 1617, 1592, 1400, 1306, 1209, 1171, 1039, 841, 801. ^1^H-NMR (CDCl_3_) δ 8.08 (d, *J* = 8.8 Hz, 1H), 7.67 (d, *J* = 2.4 Hz, 1H), 7.34 (s, 1H), 7.16 (s, 1H), 6.94 (dd, *J* = 8.8, 2.3 Hz, 1H), 6.41 (br, 1H), 4.07 (s, 3H), 4.05 (s, 3H), 3.93 (s, 3H), 3.81 (t, *J* = 6.2 Hz, 2H), 2.74 (t, *J* = 6.2 Hz, 2H), 2.37 (s, 6H). ^13^C-NMR (CDCl_3_) δ 160.2, 153.7, 153.6, 149.8, 129.4, 122.3, 114.4, 112.8, 112.1, 106.5, 104.8, 102.3, 59.4, 57.7, 56.2, 55.6, 45.9, 44.6, 39.8. ESI-MS *m*/*z*: 356.19 ([M + H]^+^). Anal. Calcd for C_20_H_25_N_3_O_3_: C 67.58; H 7.09; N 11.82; O 13.50. Found: C 67.92; H 7.15; N 11.70; O 13.26.

*N’-(8,9-dimethoxy-1,2,3,4-tetrahydro-benzo[c]phenanthridin-6-yl)-N,N-dimethyl-ethane-1,2-diamine* (**15b**) Yellow solid, yield 78.%, m.p. 121.6–123.0 °C. FTIR (KBr, cm^−1^) 3430, 3134, 2926, 1593, 1530, 1488, 1401, 1262, 1253, 1207, 1037, 837, 784. ^1^H-NMR (CDCl_3_) δ 7.98 (d, *J* = 8.3 Hz, 1H), 7.77 (s, 1H), 7.42 (s, 1H), 7.06 (d, *J* = 8.3 Hz, 1H), 6.40 (br, 1H), 4.09 (s, 3H), 4.08 (s, 3H), 3.88 (t, *J* = 5.6 Hz, 2H), 3.21 (t, *J* = 6.2 Hz, 2H), 2.91 (t, *J* = 6.2 Hz, 2H), 2.84 (t, *J* = 6.7 Hz, 2H), 2.42 (s, 6H), 1.96–1.85 (m, 4H). ^13^C-NMR (CDCl_3_) δ 152.0, 151.9, 149.1, 141.8, 136.8, 132.5, 129.8, 123.9, 118.5, 117.9, 113.1, 103.8, 103.0, 58.7, 56.5, 56.0, 45.4, 39.2, 30.3, 25.4, 23.6, 23.4. ESI-MS *m*/*z*: 380.22 ([M + H]^+^). Anal. Calcd for C_23_H_29_N_3_O_2_: C 72.79; H 7.70; N 11.07; O 8.43. Found: C 72.24; H 7.85; N 10.83; O 8.91.

*6-(2-dimethylamino-ethylamino)-8,9-dimethoxy-phenanthridine-2-carboxylic acid* (**15c**) Yellow solid, yield 69%, m.p. 192.4–193.5 °C. FTIR (KBr, cm^−1^) 3451, 3142, 1687, 1619, 1590, 1511, 1398, 1262, 1209, 1115, 1033, 843. ^1^H-NMR (CDCl_3_) δ 8.95 (s, 1H), 8.13-8.11 (m, 1H), 7.89 (s, 1H), 7.73 (d, *J* = 8.3, Hz, 1H), 7.23 (s, 1H), 6.24 (br, 1H), 4.14 (s, 3H), 4.06 (s, 3H), 3.83-3.79 (m, 2H), 2.70 (t, *J* = 5.3 Hz, 2H), 2.34 (s, 6H). ^13^C-NMR (CDCl_3_) δ 167.4, 154.4, 152.4, 149.9, 148.1, 129.4, 128.4, 126.8, 124.4, 123.6, 120.1, 113.8, 103.4, 103.3, 58.2, 56.4, 56.4, 45.4, 38.9, 29.8. ESI-MS *m*/*z*: 384.17([M + H]^+^). Anal. Calcd for C_21_H_25_N_3_O_4_: C 65.78; H 6.57; N 10.96; O 16.69. Found: C 65.59; H 6.71; N 10.95; O 16.45.

### 3.10. General Procedure for the Synthesis of Compounds ***16a**–**b***

CH_3_I (158.2 mg, 1.11 mmol) was added to the suspension of compound **7a** (100 mg, 0.37 mmol) in dry toluene, and the mixture was stirred for 16 h at 80 °C. After cooled to room temperature, the solution was filtrated and washed with toluene and ether to obtain compound **16a**. The same method was used for **16b** from compound **7c**.

*3,8,9-trimethoxy-5-methyl-phenanthridinium iodide* (**16a**) Yellow solid, yield 84%, m.p. 245.2–247.2 °C. FTIR (KBr, cm^−1^) 3423, 3131, 1624, 1501, 1402, 1294, 1232, 1172, 1033, 836. ^1^H-NMR (DMSO-d_6_) δ 9.81 (s, 1H), 9.09 (d, *J* = 9.2 Hz, 1H), 8.30 (s, 1H), 7.82 (s, 1H), 7.71–7.66 (m, 2H), 4.55 (s, 3H), 4.19 (s, 3H), 4.09 (s, 3H), 4.00 (s, 3H). ^13^C-NMR (DMSO-d_6_) δ 161.8, 158.8, 151.5, 151.0, 135.9, 132.9, 127.0, 120.0, 119.3, 118.5, 109.9, 103.2, 101.0, 57.7, 56.9, 56.7, 45.8. ESI-MS *m*/*z*: 284.14 ([M + H]^+^). Anal. Calcd for C_17_H_18_NO_3_: C 49.65; H 4.41; N 3.41; O 11.67. Found: C 49.52; H 4.32; N 3.49; O 12.17.

*2-fluoro-8,9-dimethoxy-5-methyl-phenanthridinium iodide* (**16b**) Yellow solid, yield 75%, m.p. 246.7–247.7 °C.FTIR (KBr, cm^−1^) 3438, 3120, 3016, 1606, 1516, 1474, 1438, 1400, 1279, 1205, 1131, 1036, 988, 876. ^1^H-NMR (DMSO-d_6_) δ 9.89 (s, 1H), 9.08 (d, *J* = 10.3 Hz, 1H), 8.55–8.52 (m, 1H), 8.40 (s, 1H), 8.03–7.99 (m, 1H), 7.92 (s, 1H), 4.59 (s, 3H), 4.20 (s, 3H), 4.03 (s, 3H). ^13^C-NMR (DMSO-d_6_) δ 162.1 (d, *J* = 249.6 Hz), 158.6, 152.0, 151.9, 131.7 (d, *J* = 3.8 Hz), 130.0, 127.2 (d, *J* = 9.9 Hz), 123.3 (d, *J* = 9.6 Hz), 120.3 (d, *J* = 25.1 Hz), 119.6, 110.7, 110.4 (d, *J* = 25.0 Hz), 104.7, 58.0, 56.8, 46.1. ESI-MS *m*/*z*: 272.11 ([M + H]^+^). Anal. Calcd for C_16_H_15_FINO_2_: C 48.14; H 3.79; N 3.51; O 8.02. Found: C48.30; H 3.72; N 3.62; O 8.43.

### 3.11. Cytotoxicity Assay In Vitro

All cell lines used in this study, including HepG2, A549, NCI-H460, and CNE1, were purchased from the cell bank of Chinese Academy of Sciences (Shanghai, China) and cultured at 37 °C in a humidified atmosphere containing 5% CO_2_. HepG2, A549 and CNE1 cell lines were cultured in Dulbecco’s Modified Eagle’s Medium (DMEM) supplemented with 10% (*v*/*v*) FBS, 100 U/mL penicillin and 100 μg/mL streptomycin. NCI-H460 cell line was cultured in ATCC Modified 1640 Medium supplemented with 10% (*v*/*v*) FBS, 100 U/mL penicillin and 100 μg/mL streptomycin.

The antitumor activity of new compounds was evaluated by MTT assay in vitro. 5 × 10^3^ cells in 100 μL medium were plated to each well of a 96-well flat-bottom microtiter plate and cultured overnight. Then the cells were treated with serial diluted candidate compounds. The control was treated with equivalent DMSO. After 48 h, 10 μL MTT stock (5 mg/mL) was added to each well and incubated for 2 h. Then, the supernatants were discarded and 100 μL DMSO was added to each well. After 10 minutes’ shaking, the optical density at the wavelengths of 490 nm (OD_490_) was measured on a SPARK microplate reader (Tecan, Männedorf, Switzerland). All samples were repeated 3 times and each time tested in triplicate. The cell inhibition rate of each sample was calculated by the following formula. The IC_50_ values of the compounds were calculated using SPSS 17.0 software.

Inhibition (%) = [1 − (OD_sample_ − OD_blank_)/(OD_control_ − OD_blank_)] × 100%. (1)

## 4. Conclusions

In conclusion, we have synthesized series of structurally simple phenanthridine analogues based on nitidine and evaluated their antitumor activities against human cancer cell lines including HepG2, A549, NCI-H460, and CNE1 cells. Most of the derivatives exhibited moderate to high activity, especially compounds **15a**, **15b**, and **15c**. It was found that the C-6 modified structure could greatly increase the antitumor activity, and the structure of ammonium salt was not necessary to the antitumor activity in the test compounds. The result motivated us to investigate more C-6 substituted derivatives and their structure–activity relationships to discover potent antitumor drugs with high activity and excellent selectivity.

## Figures and Tables

**Table 1:** Antitumor activity of some compounds.

Compounds	IC_50_ (μM)			
	HepG2	A549	NCI-H460	CNE1
**5a**	>50	36.20 ± 1.52	/	26.74 ± 3.54
**5b**	>50	44.57 ± 3.02	31.03 ± 6.91	14.75 ± 1.96
**5c**	>50	34.56 ± 3.28	/	11.25 ± 3.59
**5e**	9.07 ± 1.14	2.77 ± 0.11	12.59 ± 3.57	1.13 ± 0.17
**5h**	/	/	26.13 ± 4.35	/
**5k**	/	>50	48.01 ± 0.21	>50
**5l**	25.01 ± 4.85	/	>50	42.446 ± 1.95
**6a**	44.49 ± 2.91	/	/	>50
**6b**	>50	/	48.79 ± 1.92	>50
**7b**	>50	>50	/	4.38 ± 1.66
**7c**	33.67 ± 2.51	45.84 ± 1.79	34.69 ± 3.42	8.57 ± 1.21
**8c**	16.79 ± 5.33	/	>50	37.98 ± 4.91
**9a**	26.96 ± 3.82	41.45 ± 2.05	/	34.50 ± 2.31
**11a**	/	44.00 ± 2.90	/	23.84 ± 4.57
**14c**	>50	>50	>50	38.52 ± 1.50
**15a**	1.68 ± 0.10	1.94 ± 0.09	11.38 ± 0.53	1.20 ± 0.14
**15b**	1.19 ± 0.09	2.11 ± 0.24	8.98 ± 0.58	1.87 ± 0.18
**15c**	2.15 ± 0.17	1.37 ± 0.15	5.24 ± 0.51	1.19 ± 0.18
**16a**	25.41 ± 5.15	18.69 ± 3.34	28.09 ± 4.20	8.79 ± 1.66
**16b**	>50	33.01 ± 6.61	21.48 ± 3.96	25.43 ± 1.99
Nitidine chloride	1.40 ± 0.16	1.88 ± 0.24	2.35 ± 0.35	1.85 ± 0.08

## Supplementary Materials

The following are available online. FTIR, ^1^H-NMR, ^13^C-NMR and ESI-MS spectra of compounds.
