# Supplementary material for: Structurally Simple Phenanthridine Analogues Based on Nitidine and Their Antitumor Activities
Source: Molecules. 2019 Jan 26;24(3):437. doi: 10.3390/molecules24030437 (PMC6385102; doi:10.3390/molecules24030437)
Supplement: Supplementary file 1 [file molecules-24-00437-s001.pdf]

Supplementary Information

Structurally Simple Phenanthridine Analogues Based on Nitidine and Their Antitumor Activities

Shu-Qin Qin [1, 2], Lian-Chun Li [1], Jing-Ru Song [1,*], Hai-Yun Li [2] and Dian-Peng Li [1,*]

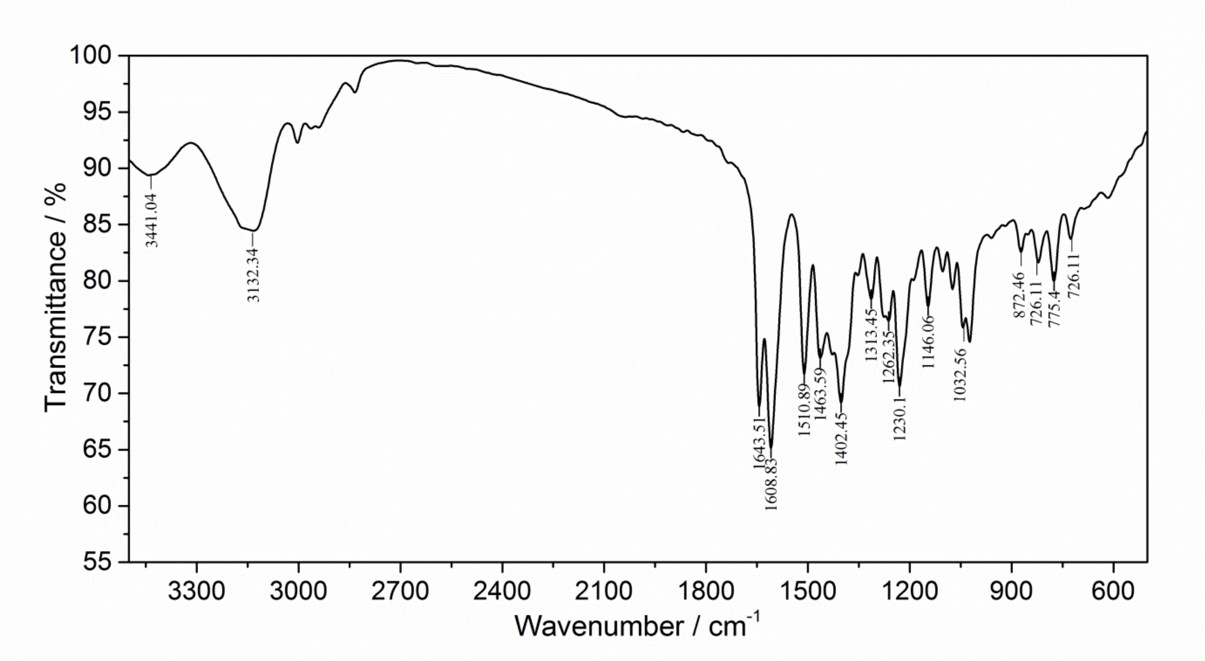

Fig. S1 FTIR of compound 5a

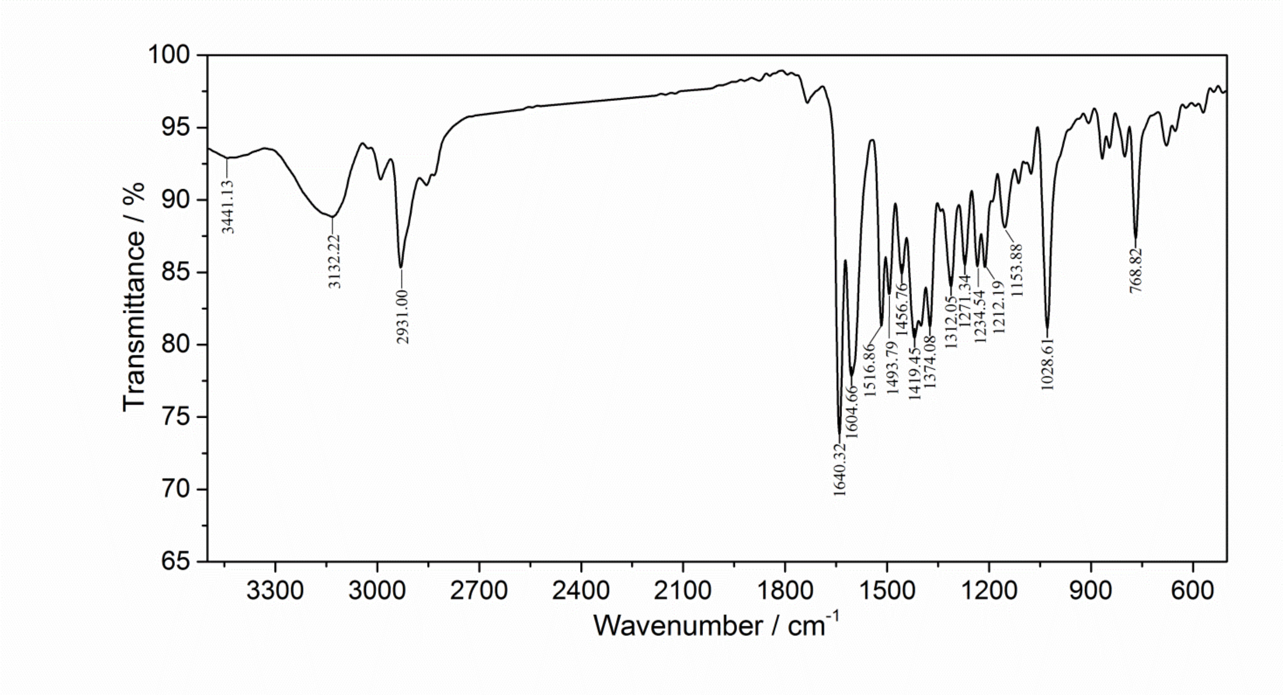

Fig. S2 FTIR of compound 5d

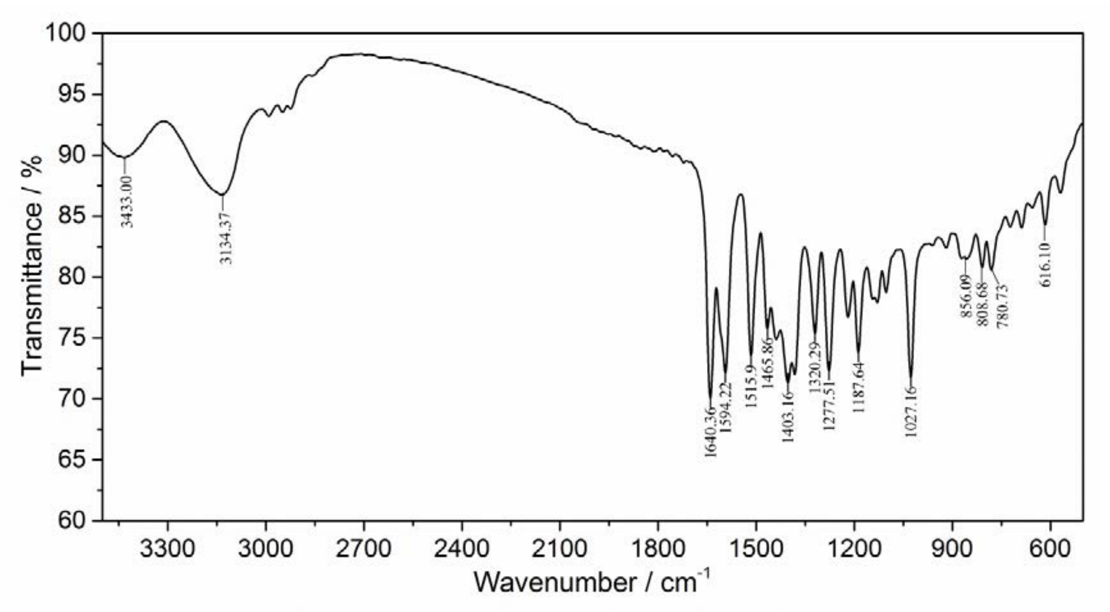

Fig. S3 FTIR of compound 5g

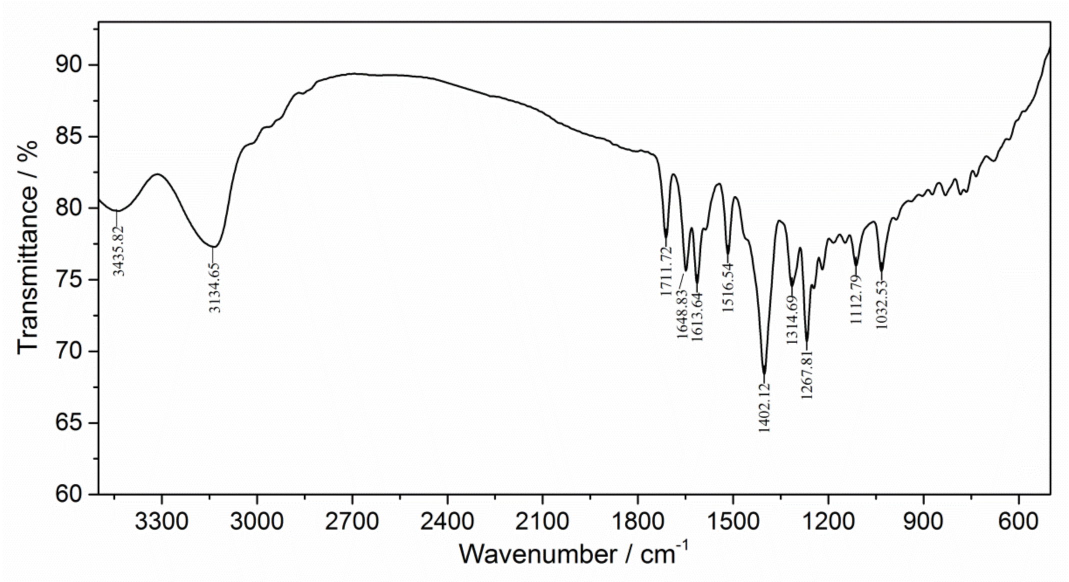

Fig. S4 FTIR of compound 5j

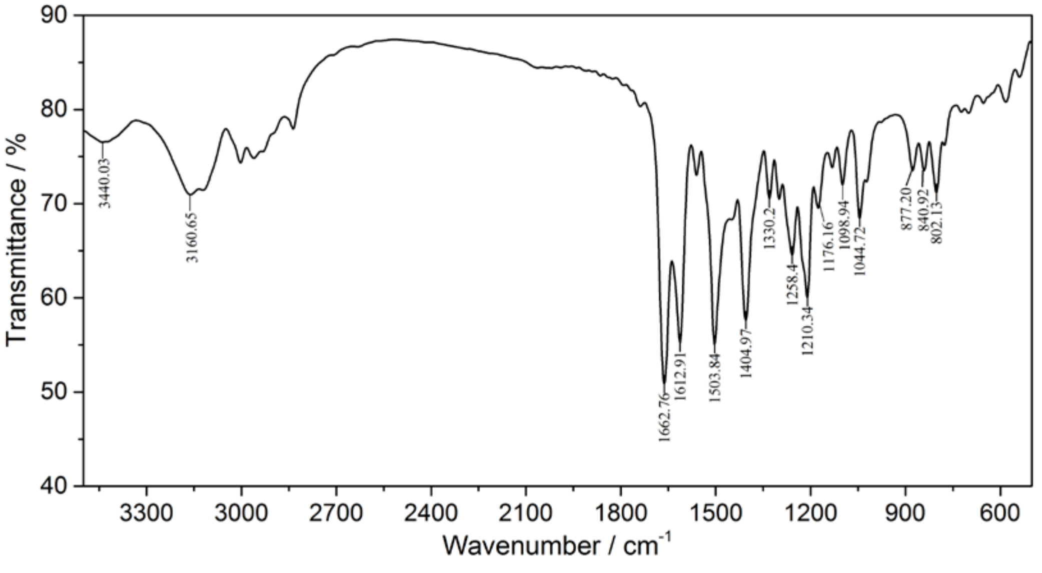

Fig. S5 FTIR of compound 6a

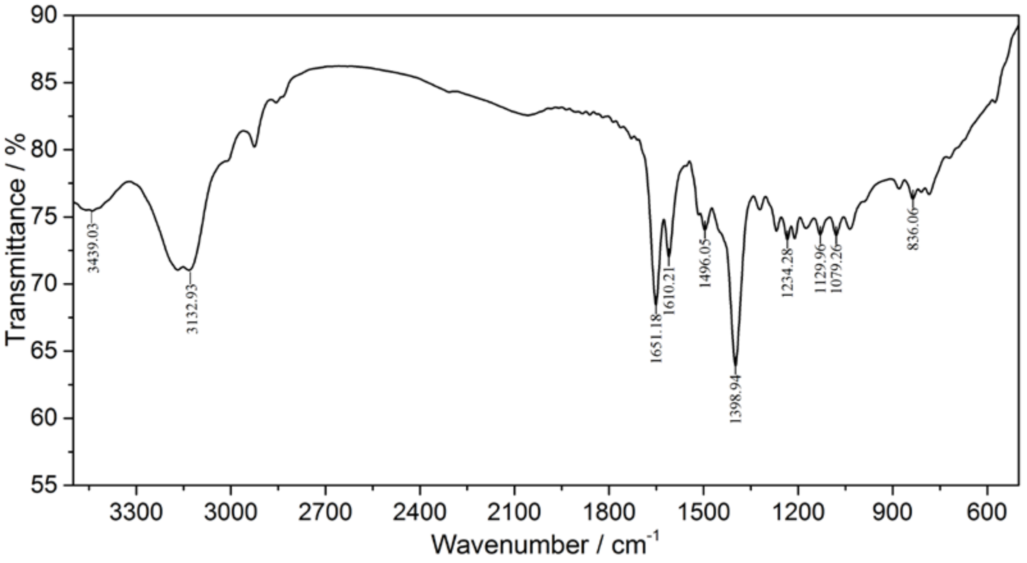

Fig. S6 FTIR of compound 6b

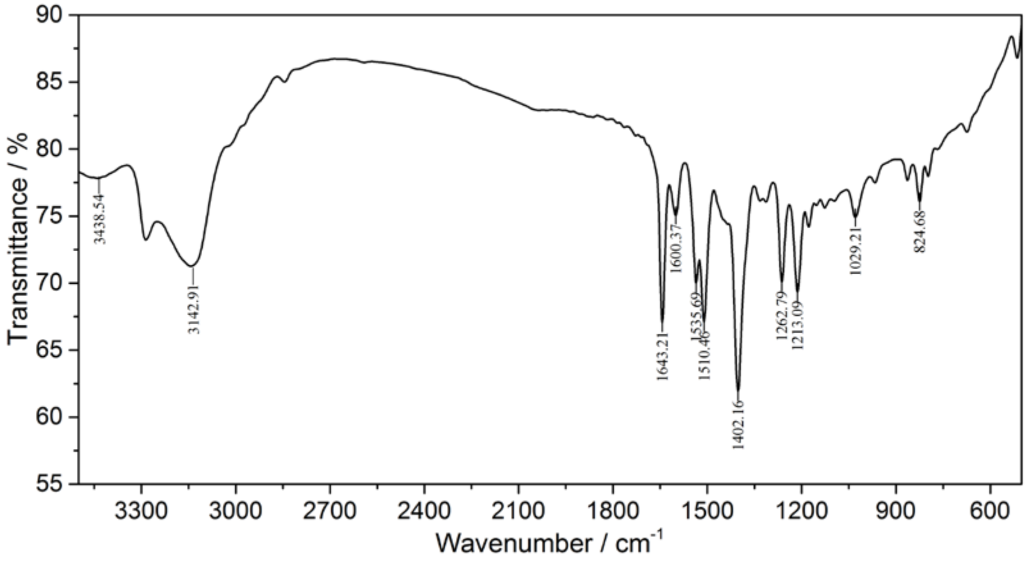

Fig. S7 FTIR of compound 6c

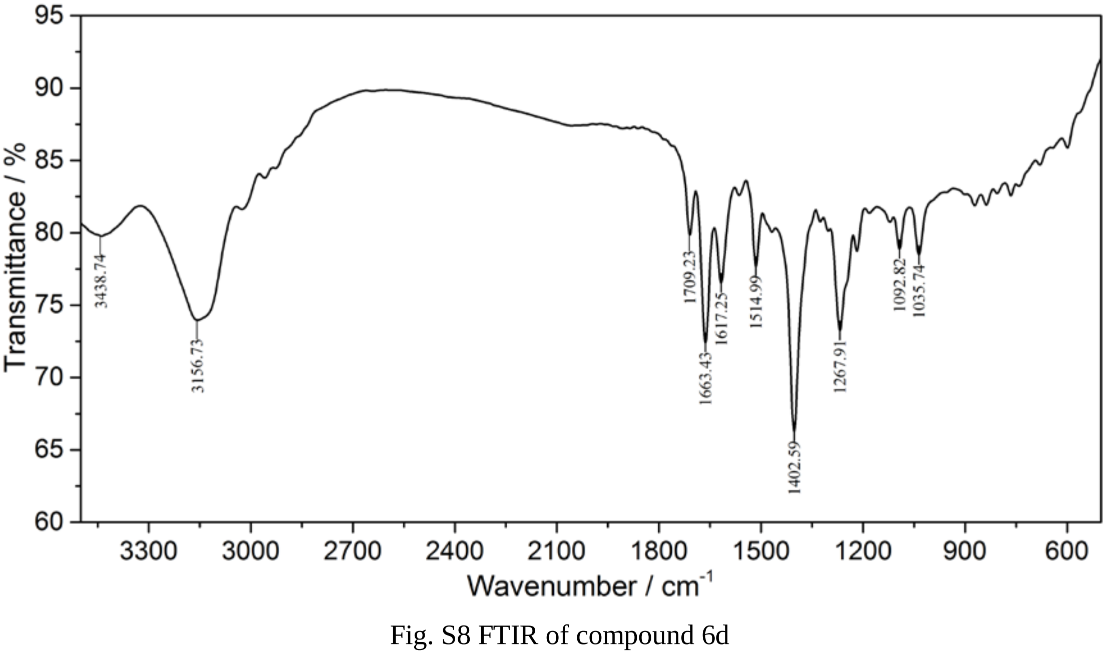

Fig. S8 FTIR of compound 6d

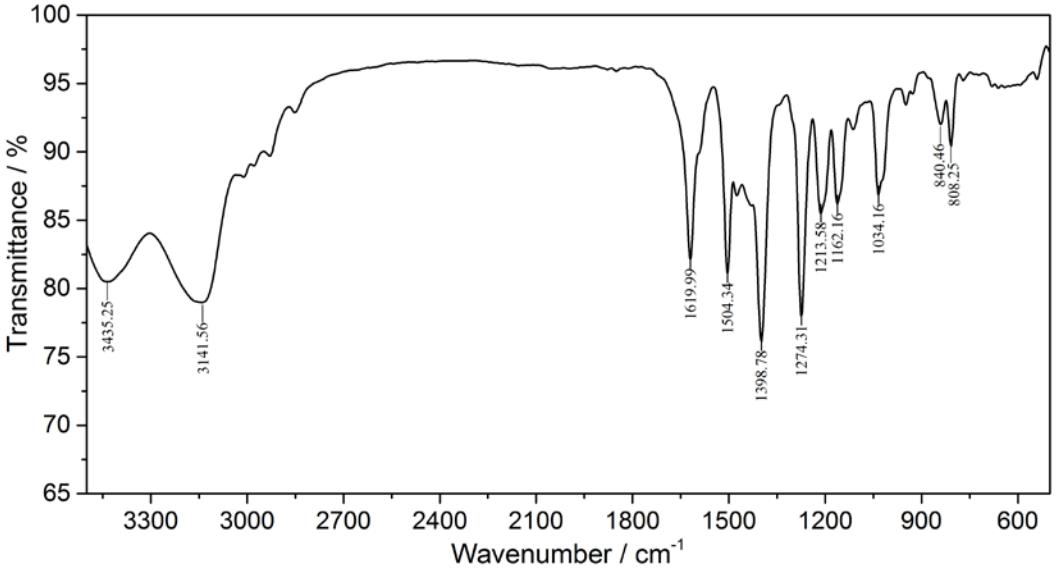

Fig. S9 FTIR of compound 7a

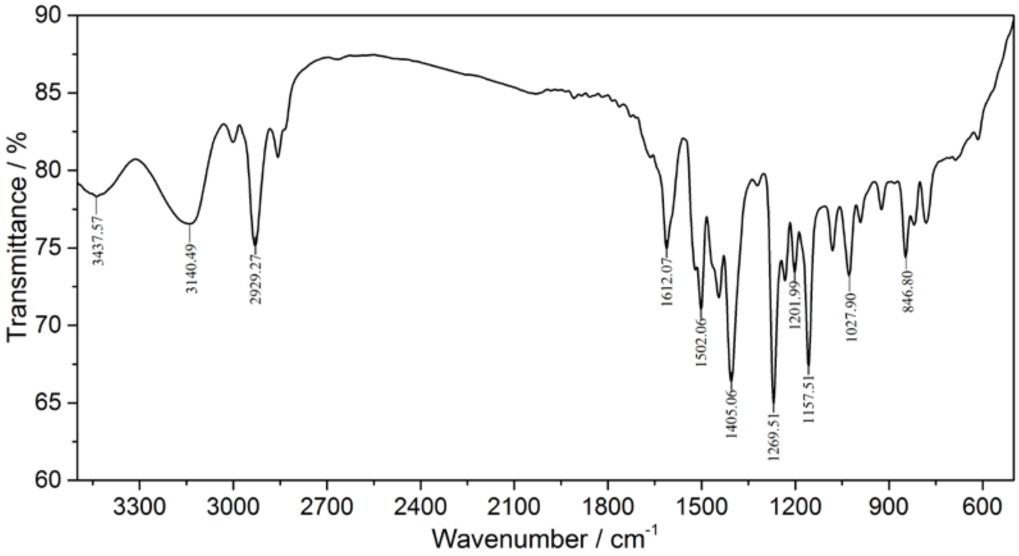

Fig. S10 FTIR of compound 7b

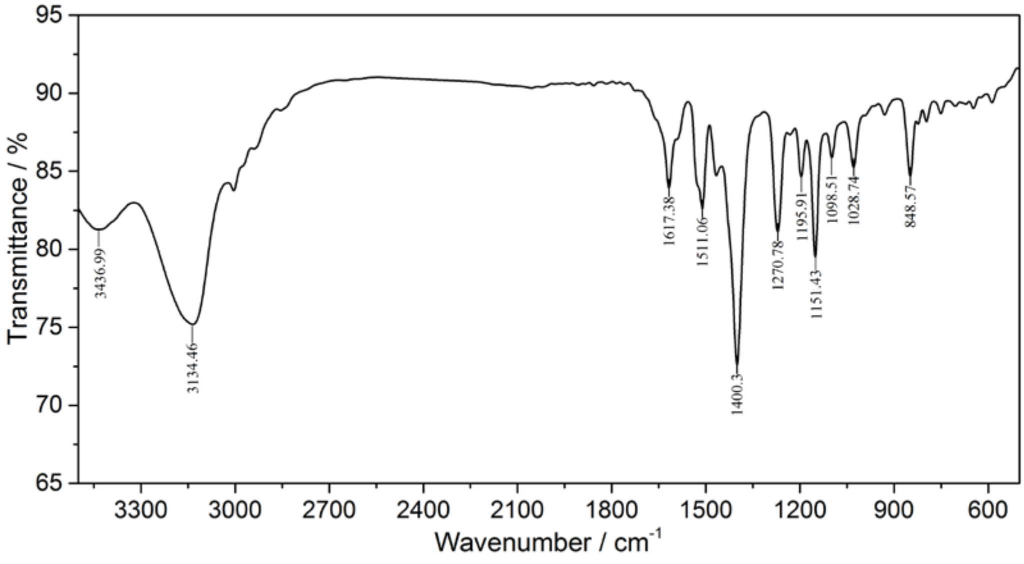

Fig. S11 FTIR of compound 7c

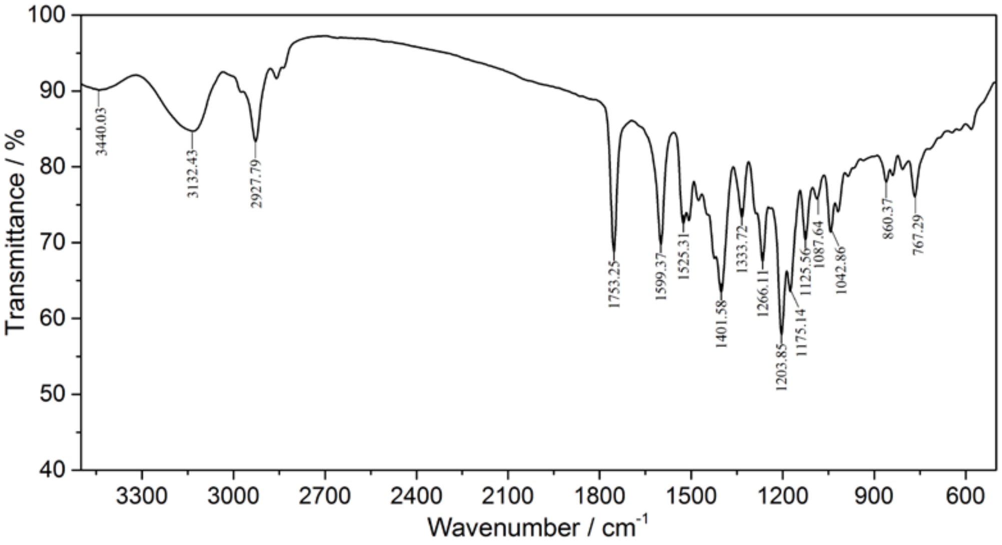

Fig. S12 FTIR of compound 8a

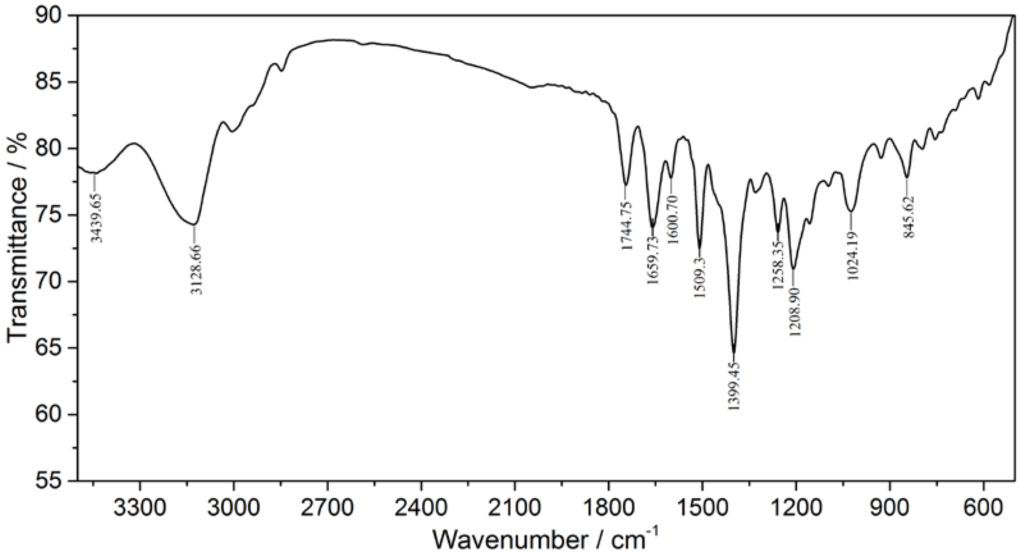

Fig. S13 FTIR of compound 8b

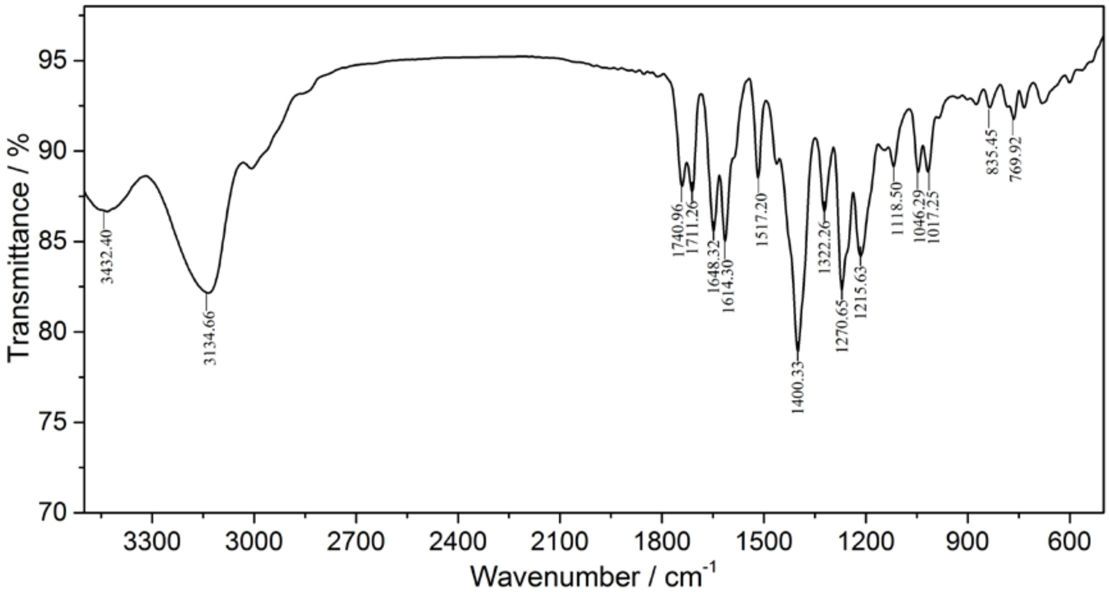

Fig. S14 FTIR of compound 8c

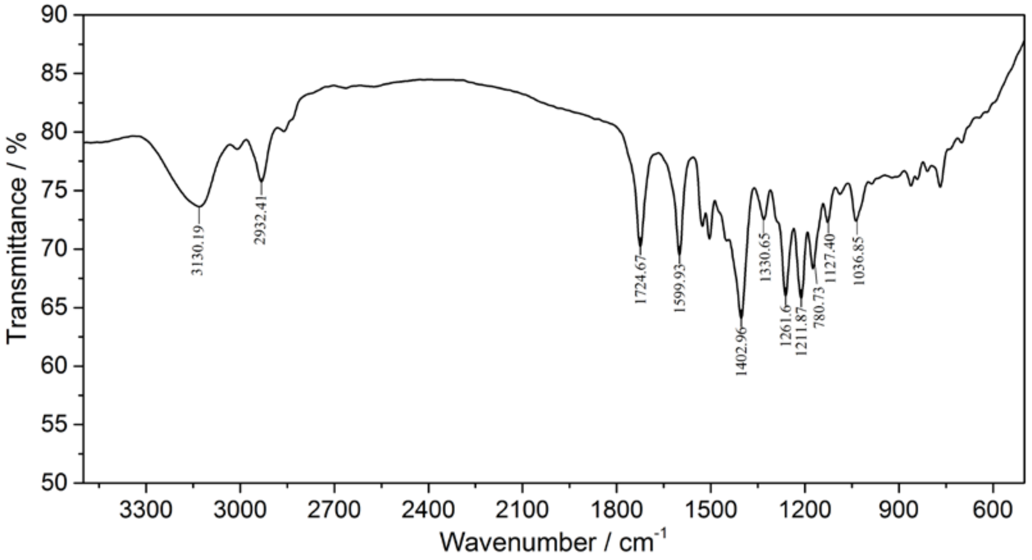

Fig. S15 FTIR of compound 9a

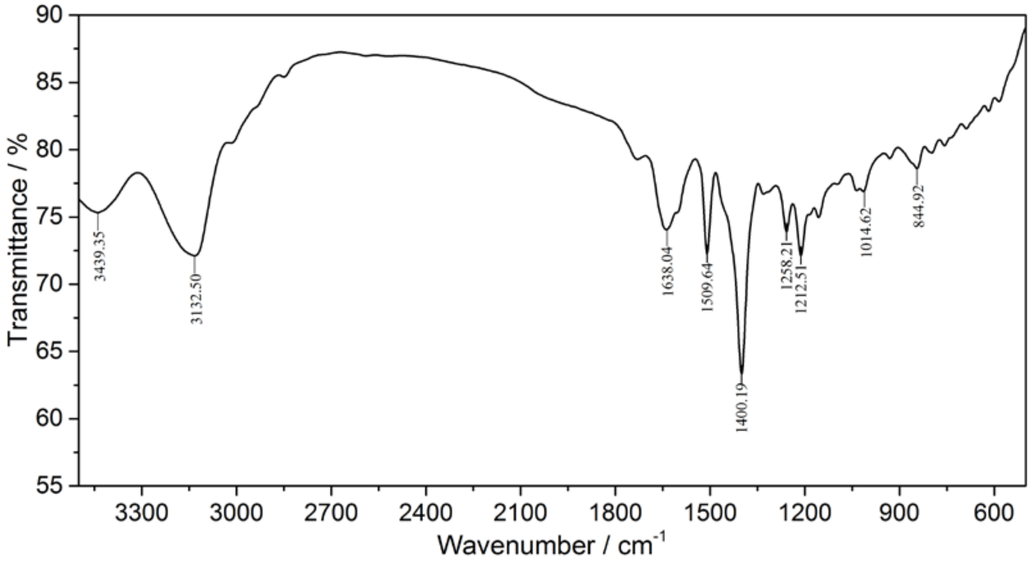

Fig. S16 FTIR of compound 9b

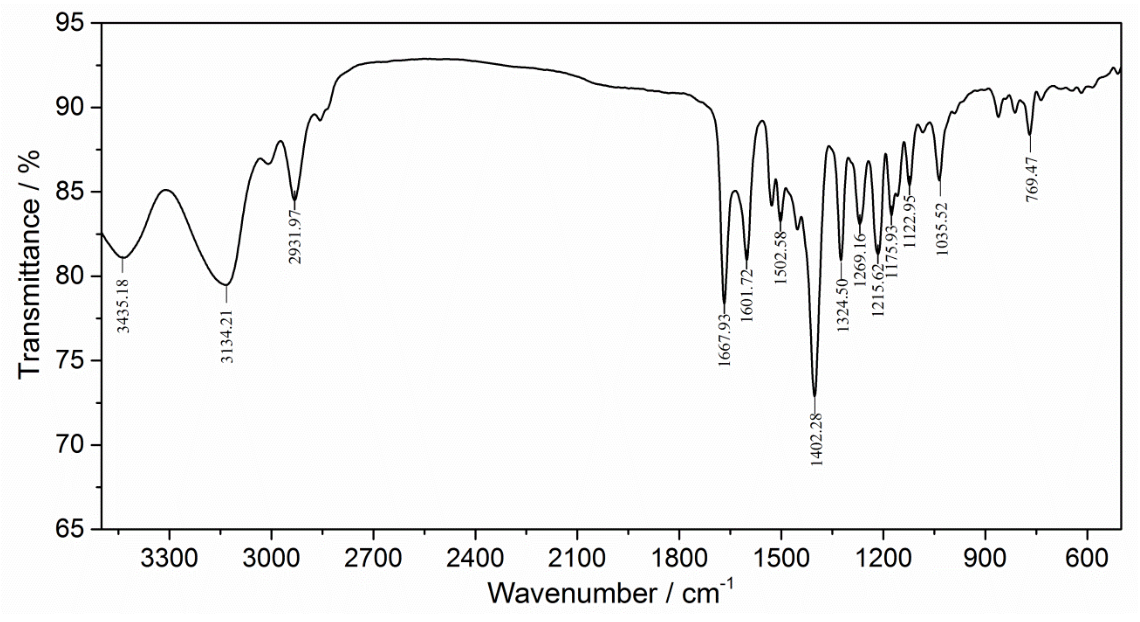

Fig. S17 FTIR of compound 10a

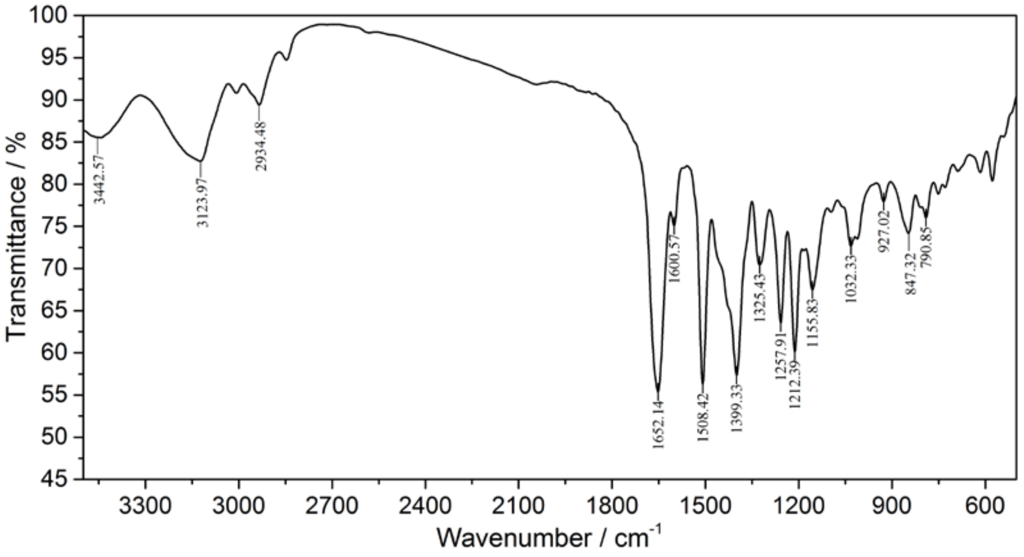

Fig. S18 FTIR of compound 10b

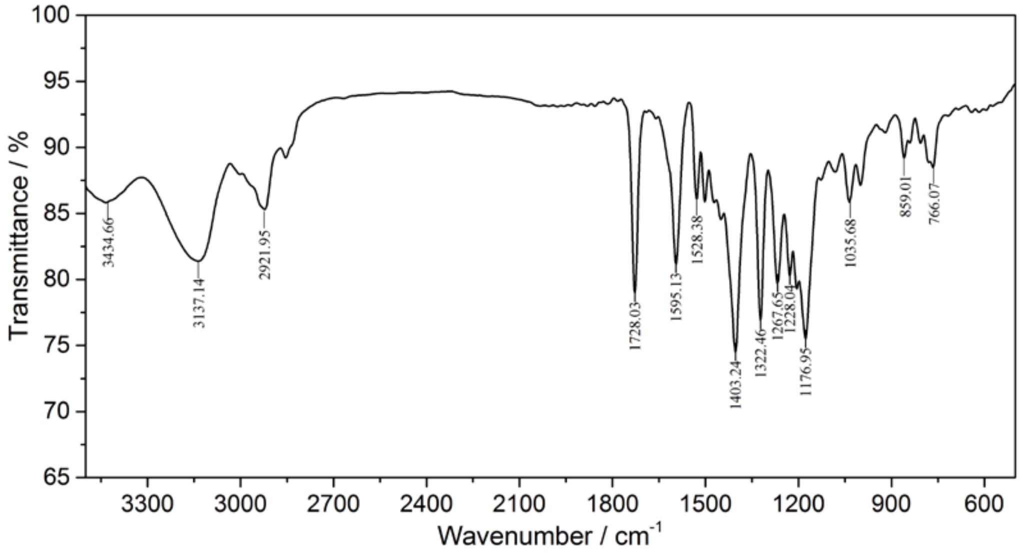

Fig. S19 FTIR of compound 11a

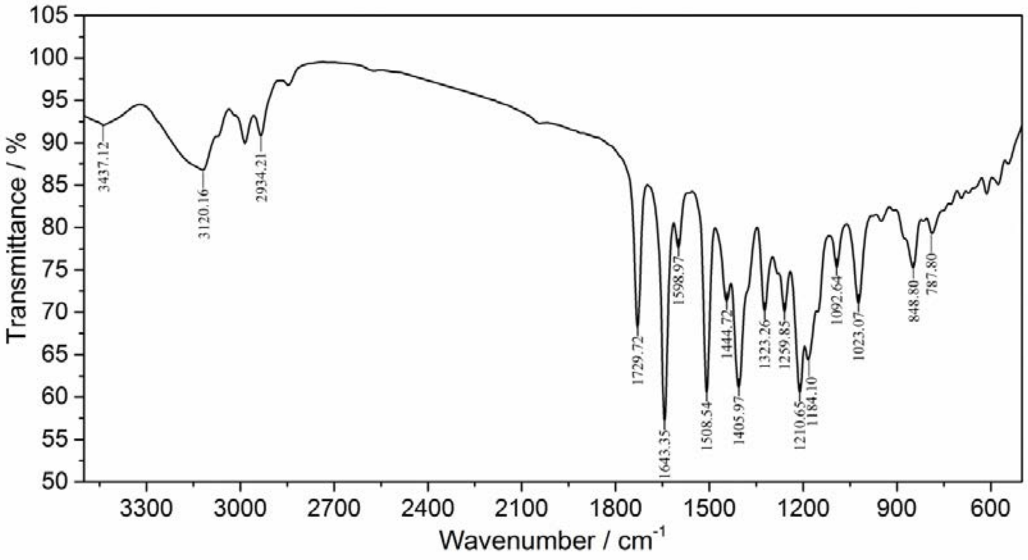

Fig. S20 FTIR of compound 11b

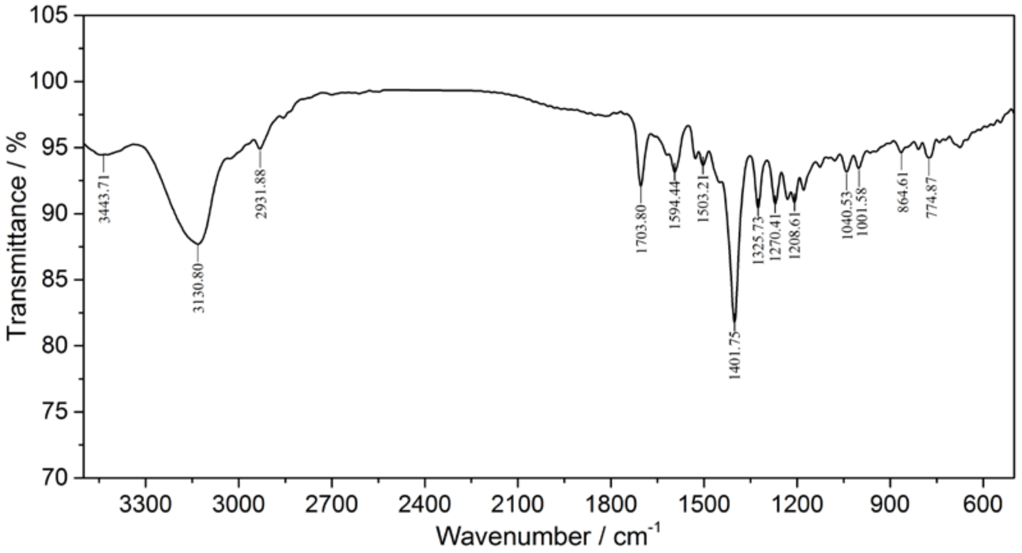

Fig. S21 FTIR of compound 12a

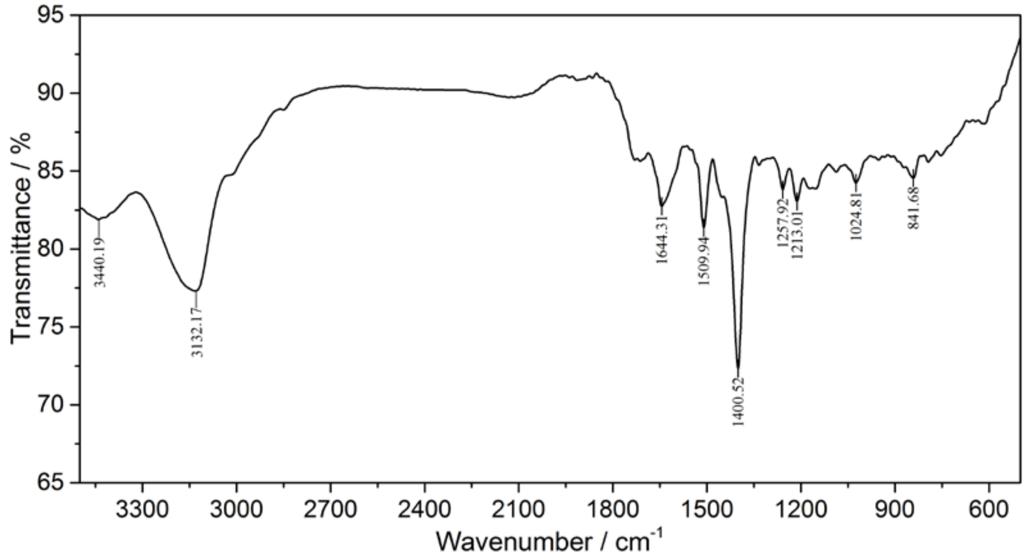

Fig. S22 FTIR of compound 12b

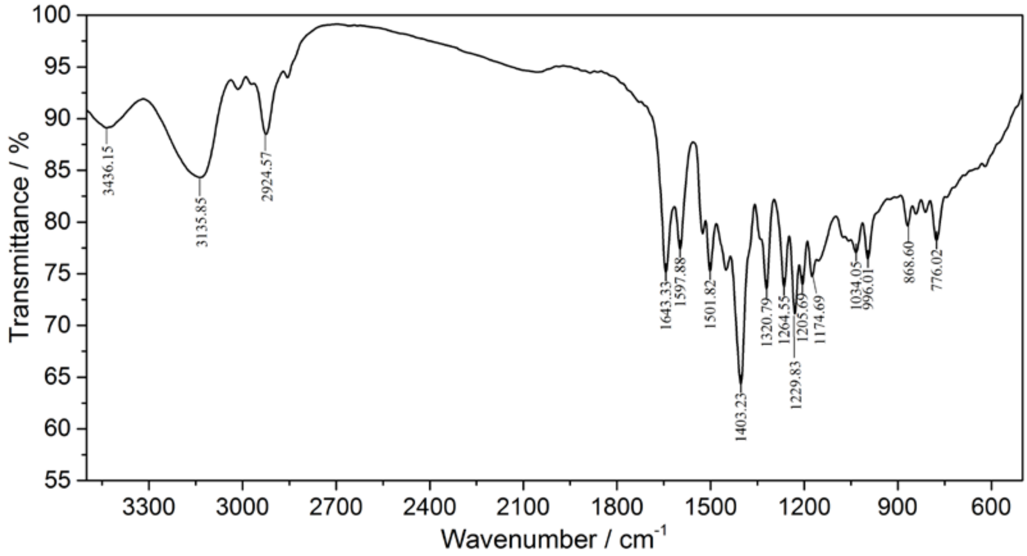

Fig. S23 FTIR of compound 13a

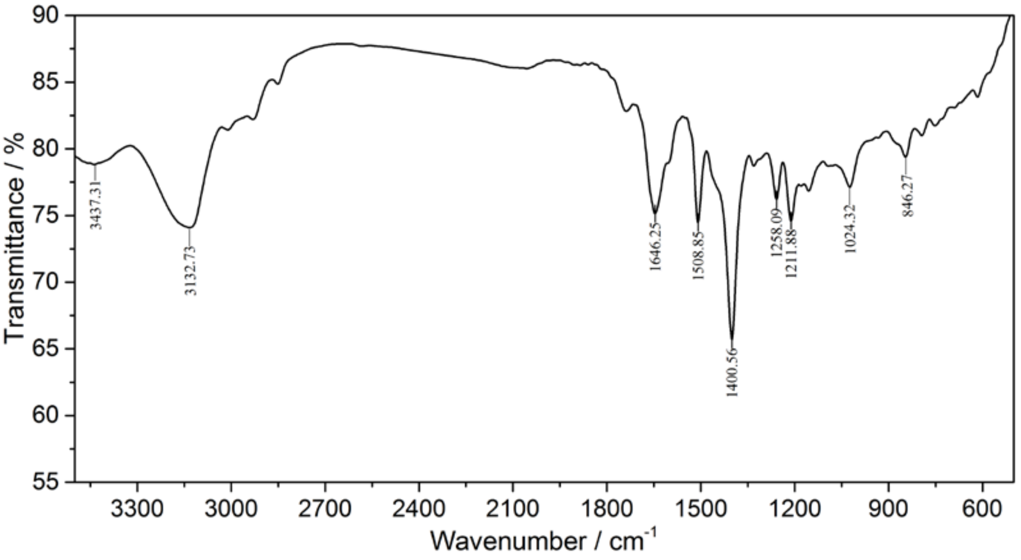

Fig. S24 FTIR of compound 13b

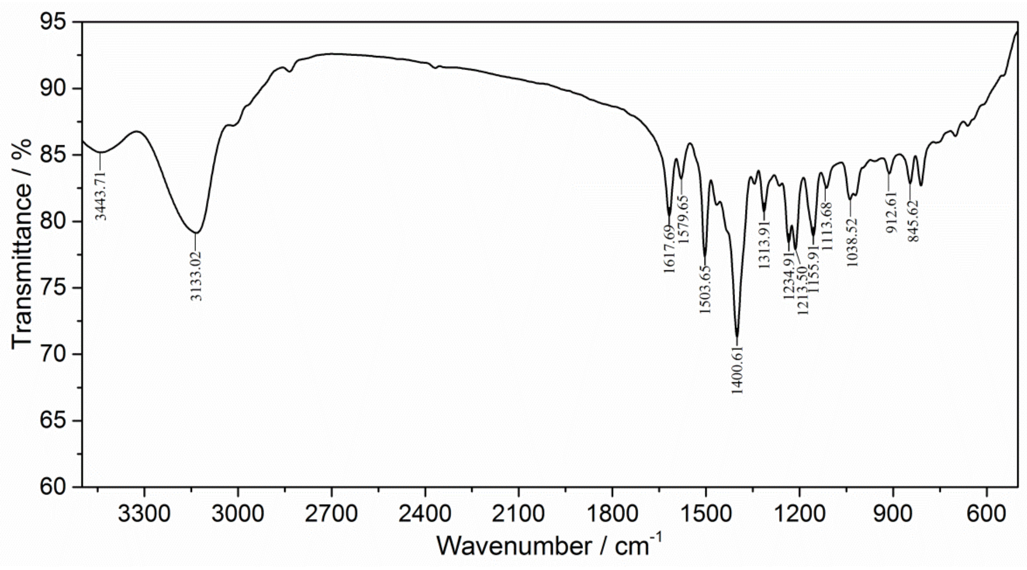

Fig. S25 FTIR of compound 14a

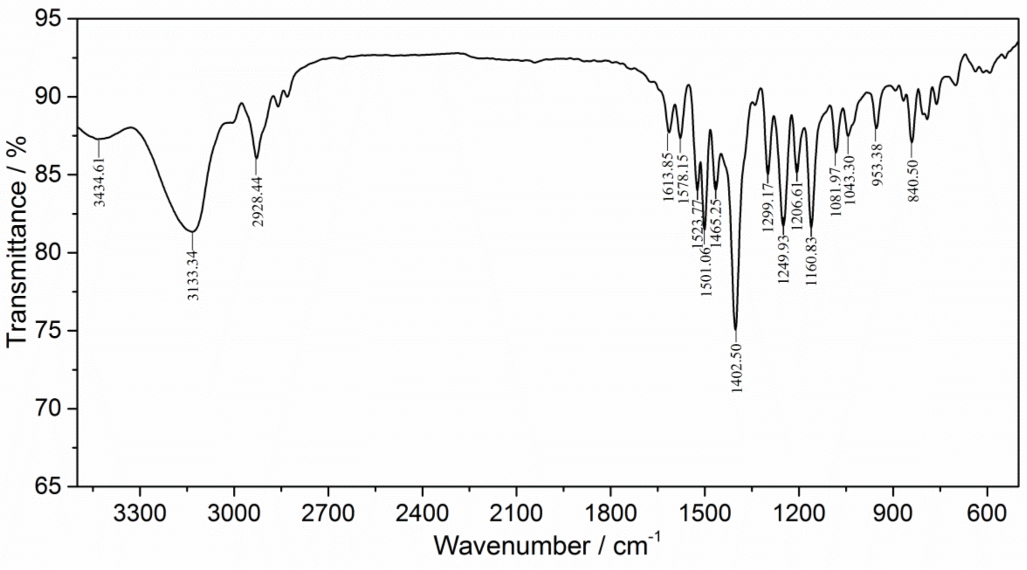

Fig. S26 FTIR of compound 14b

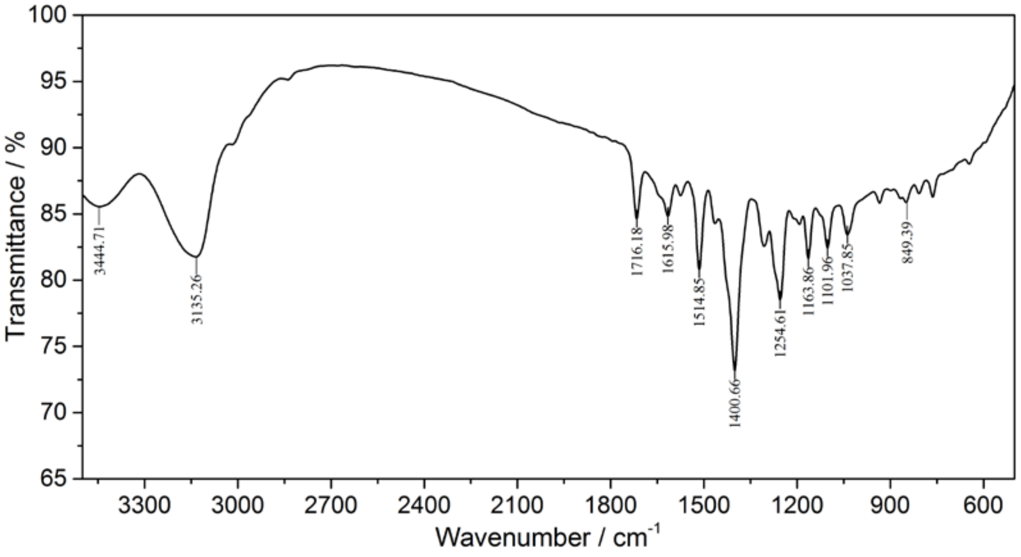

Fig. S27 FTIR of compound 14c

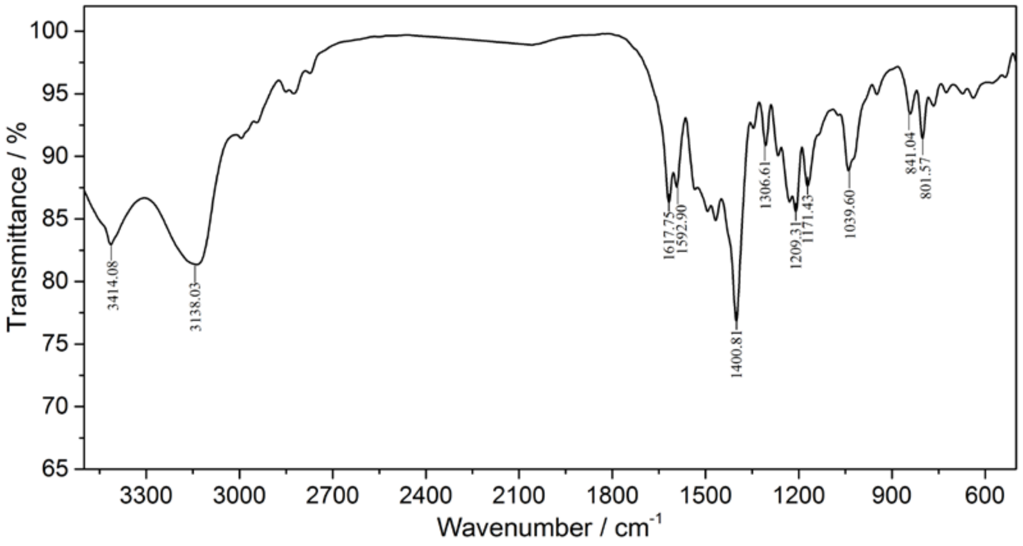

Fig. S28 FTIR of compound 15a

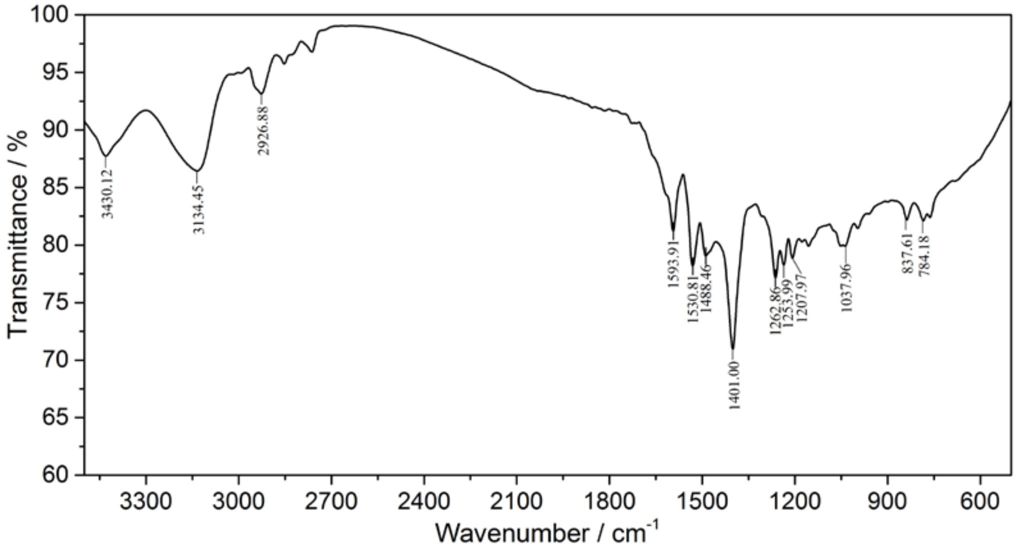

Fig. S29 FTIR of compound 15b

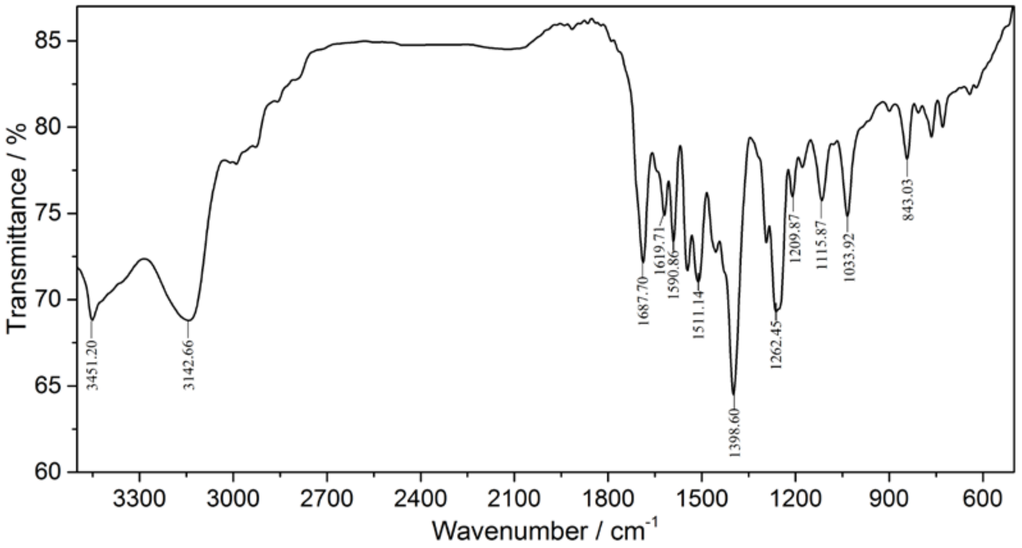

Fig. S30 FTIR of compound 15c

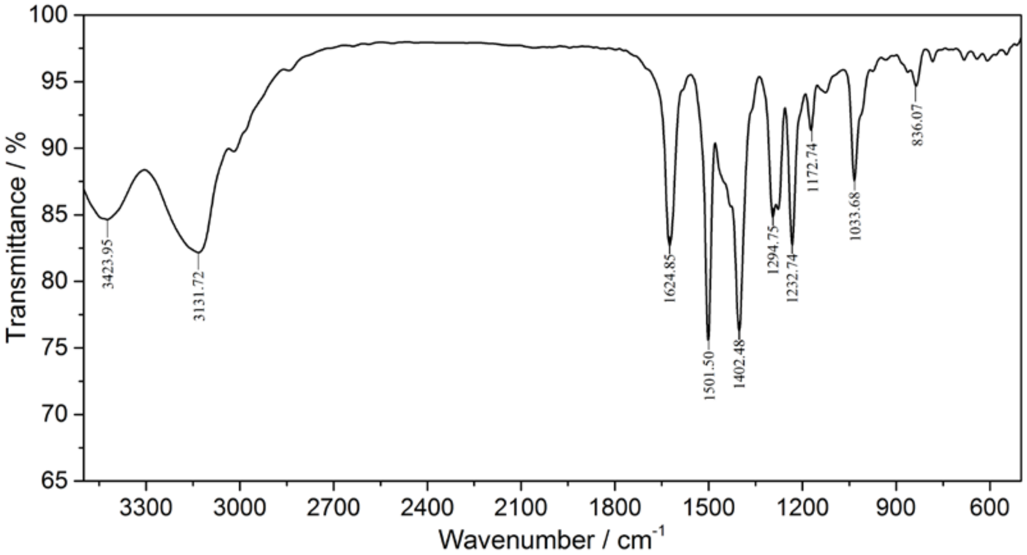

Fig. S31 FTIR of compound 16a

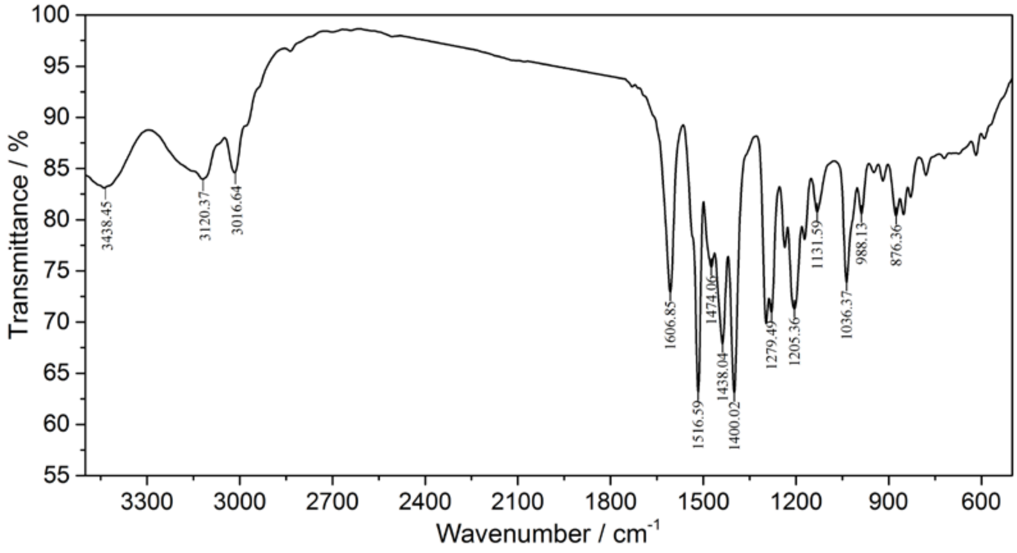

Fig. S32 FTIR of compound 16b

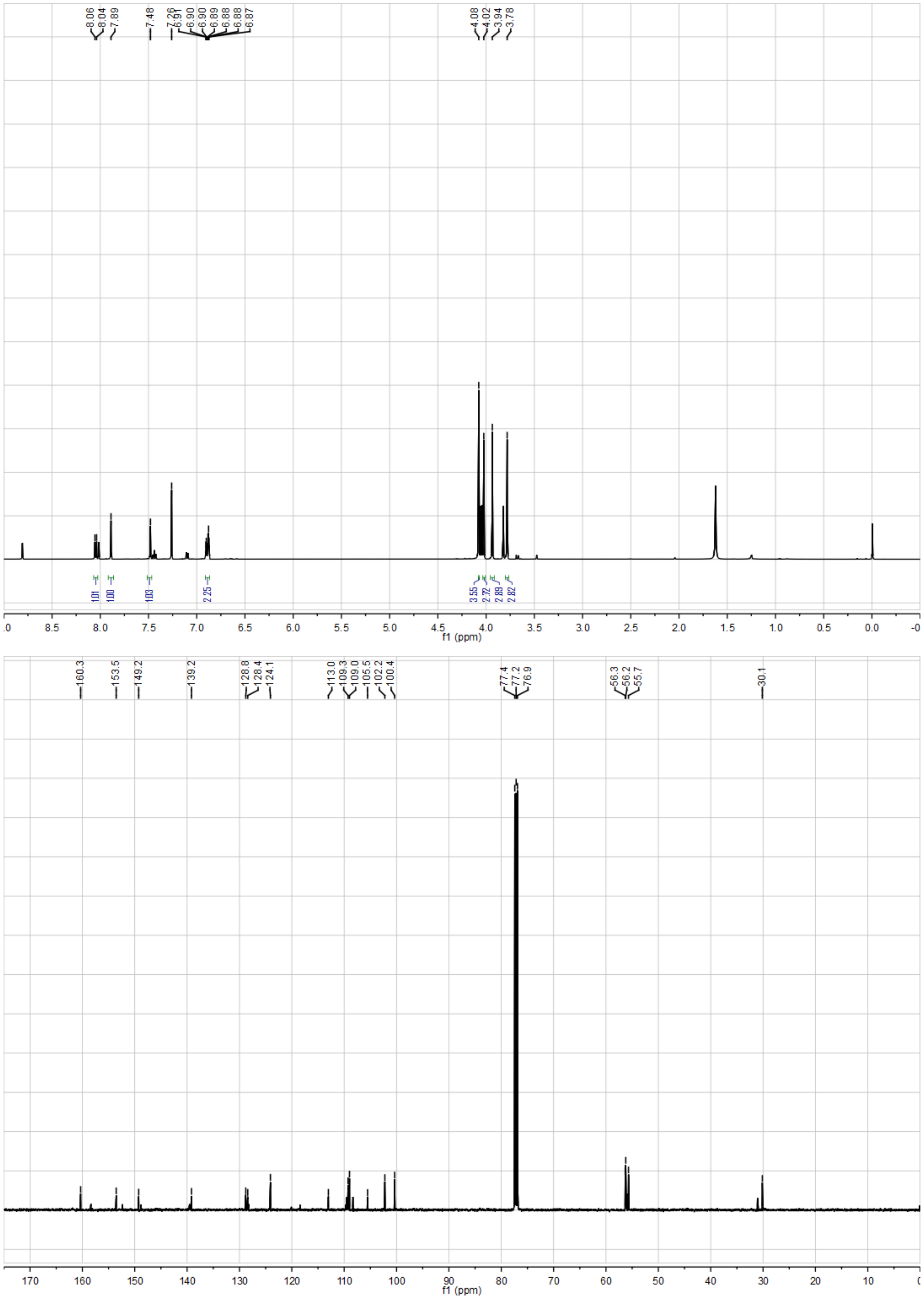

Fig. S33 NMR of compound 5a

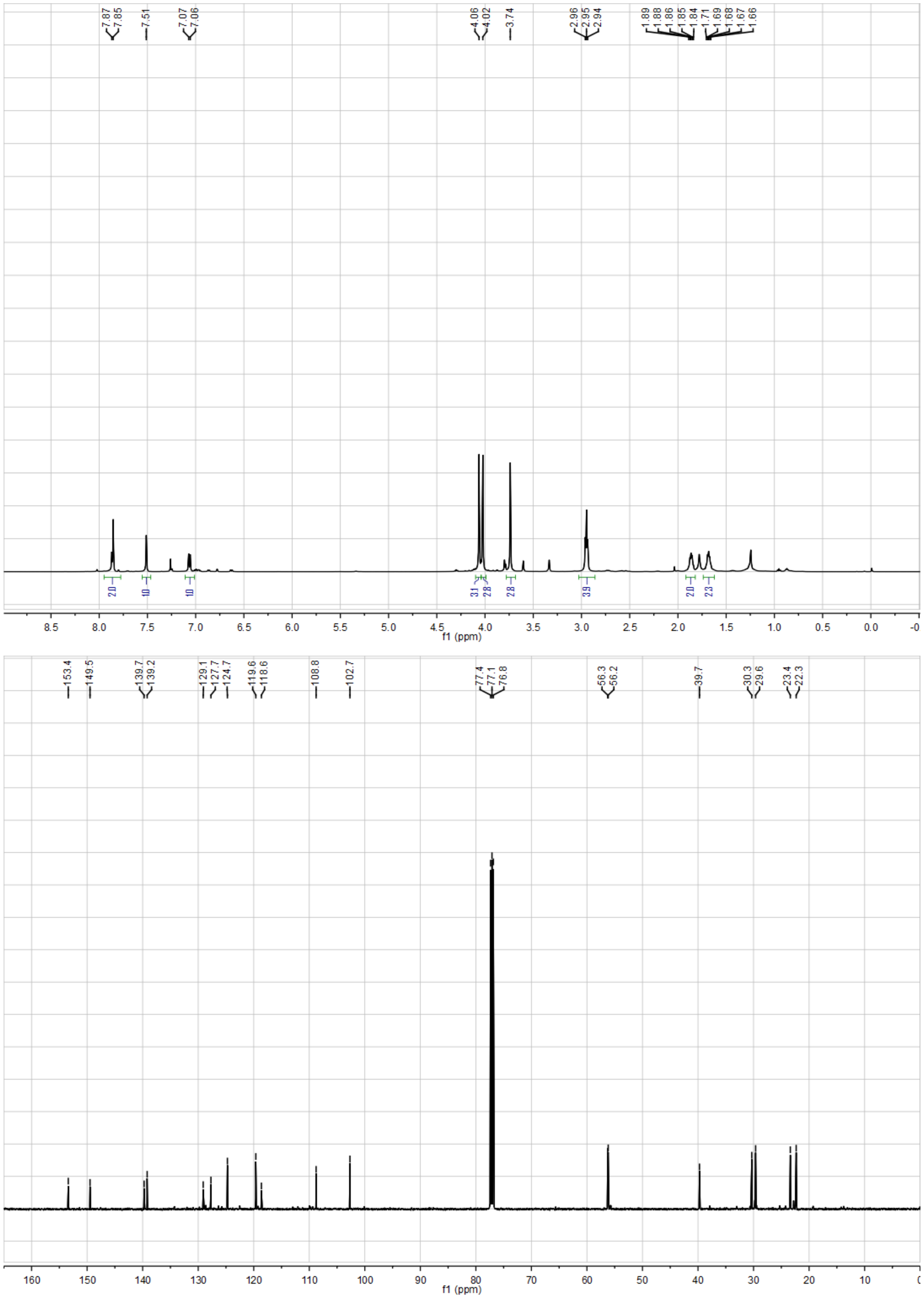

Fig. S34 NMR of compound 5d

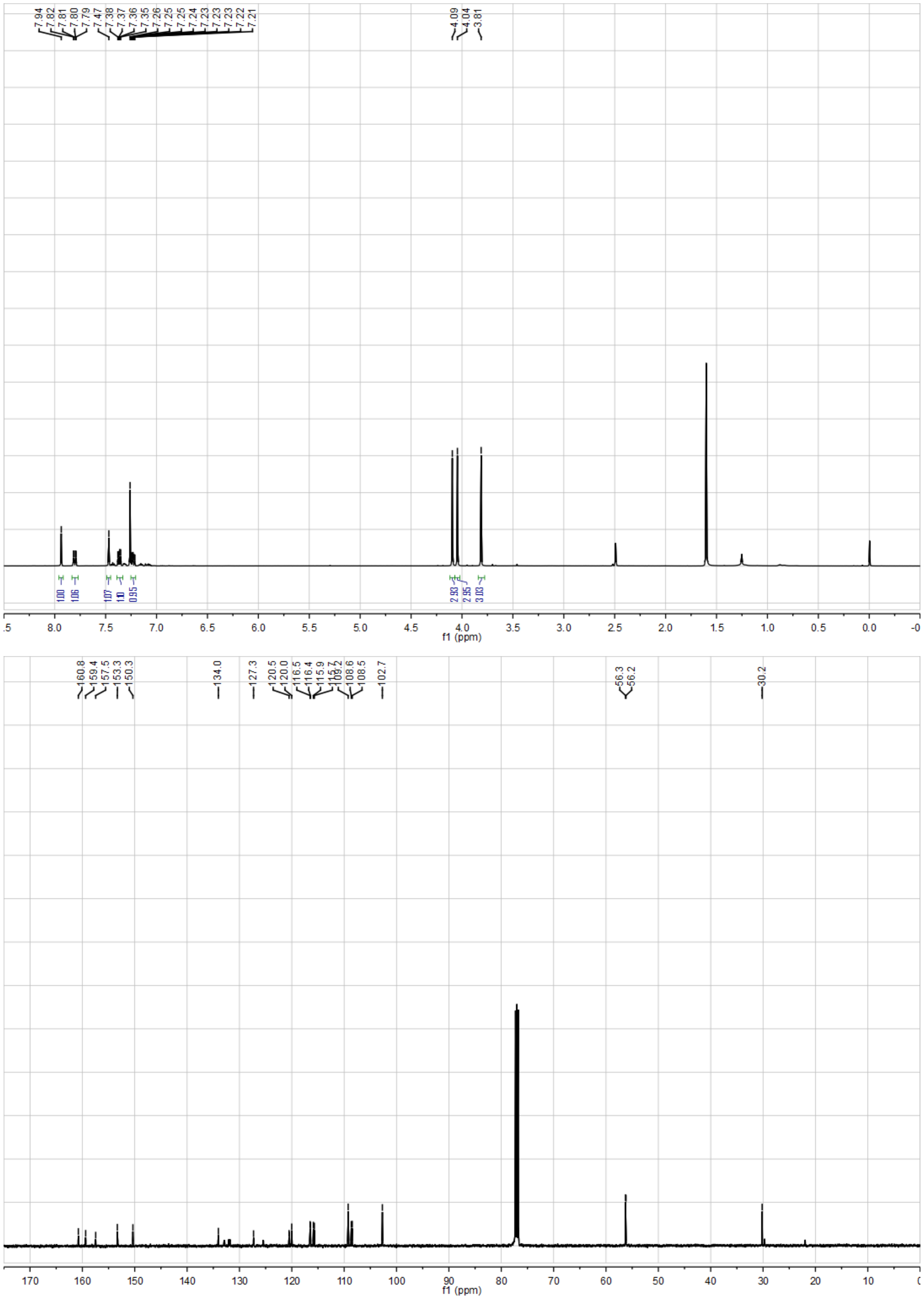

Fig. S35 NMR of compound 5g

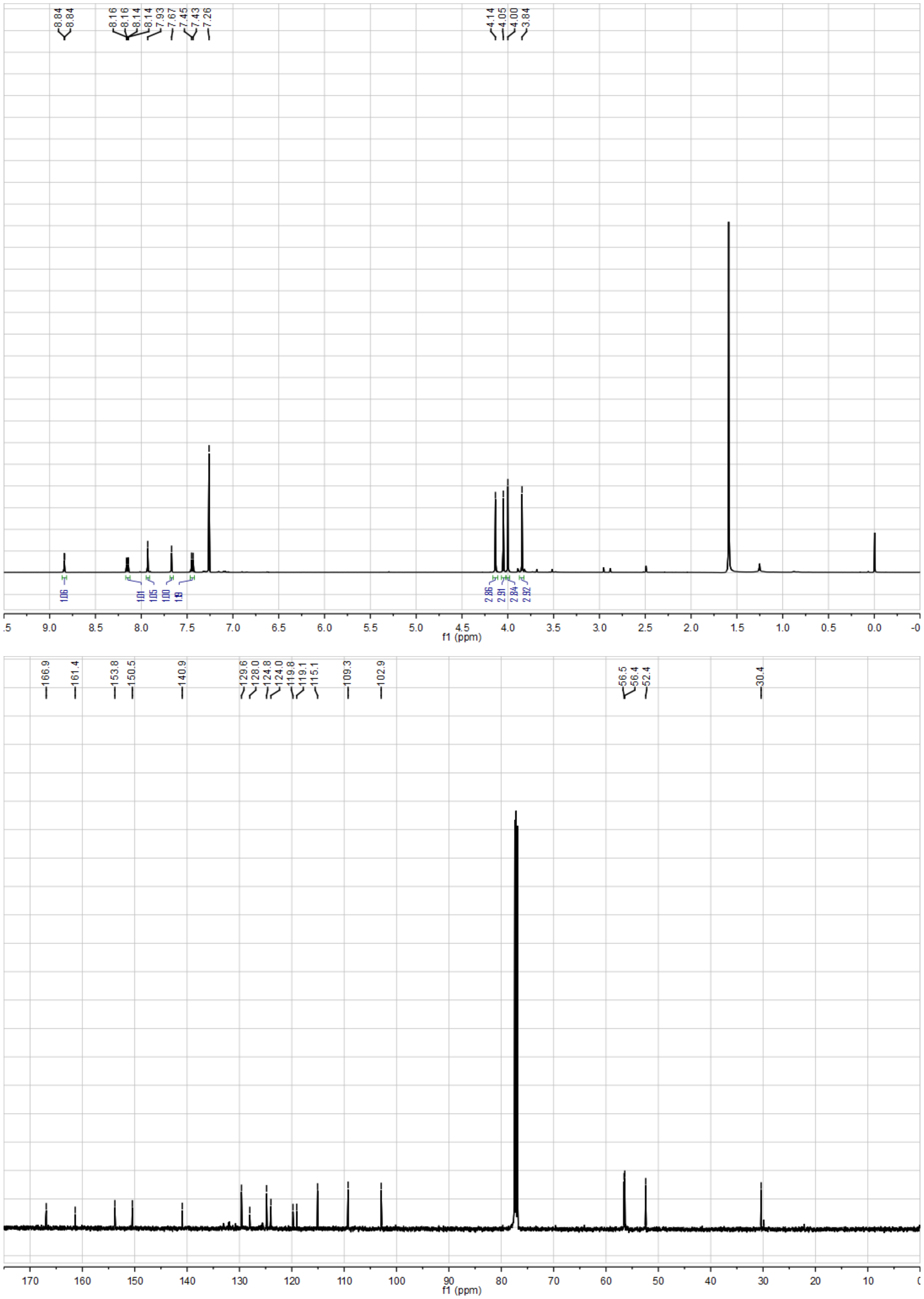

Fig. S36 NMR of compound 5j

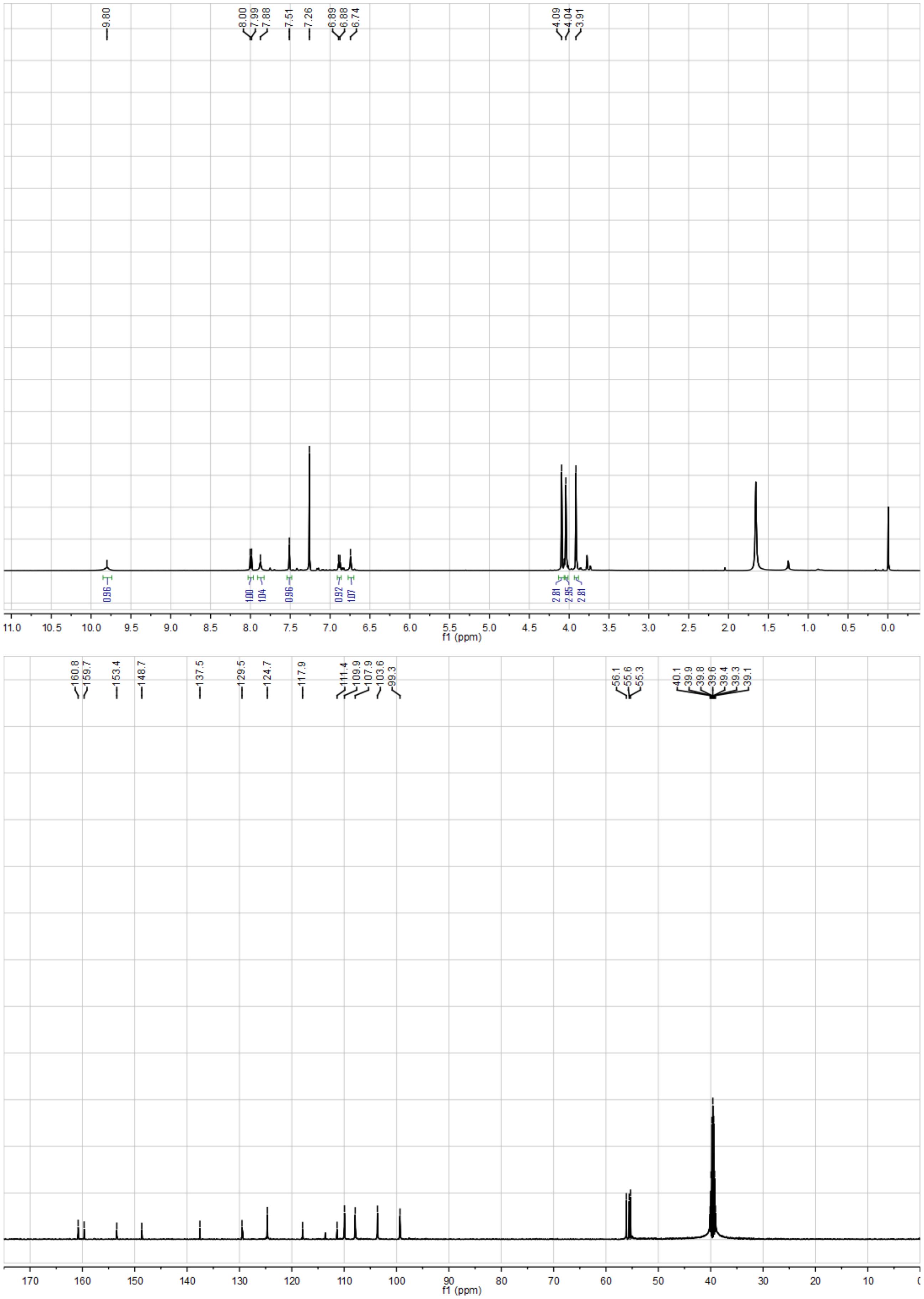

Fig. S37 NMR of compound 6a

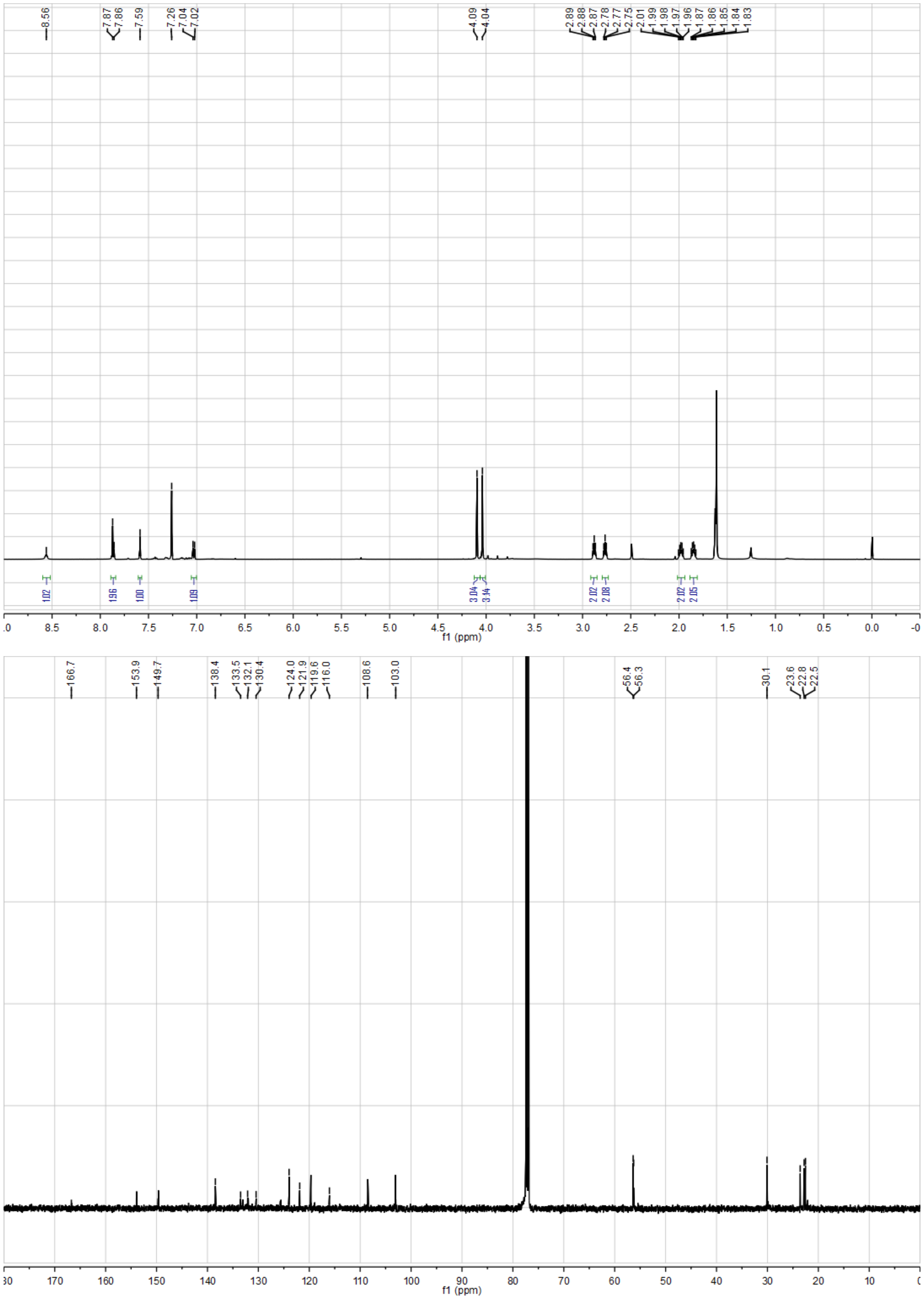

Fig. S38 NMR of compound 6b

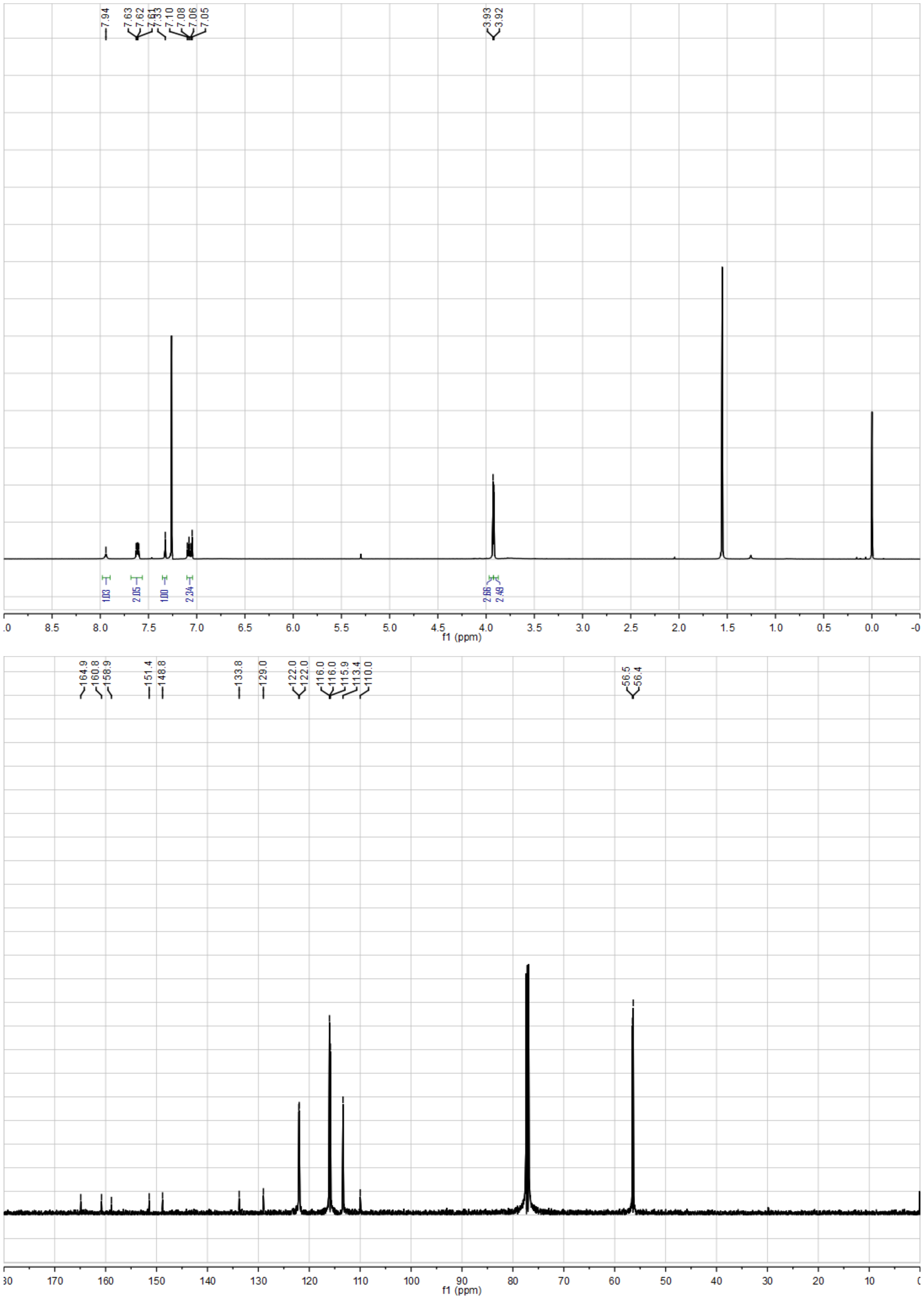

Fig. S39 NMR of compound 6c

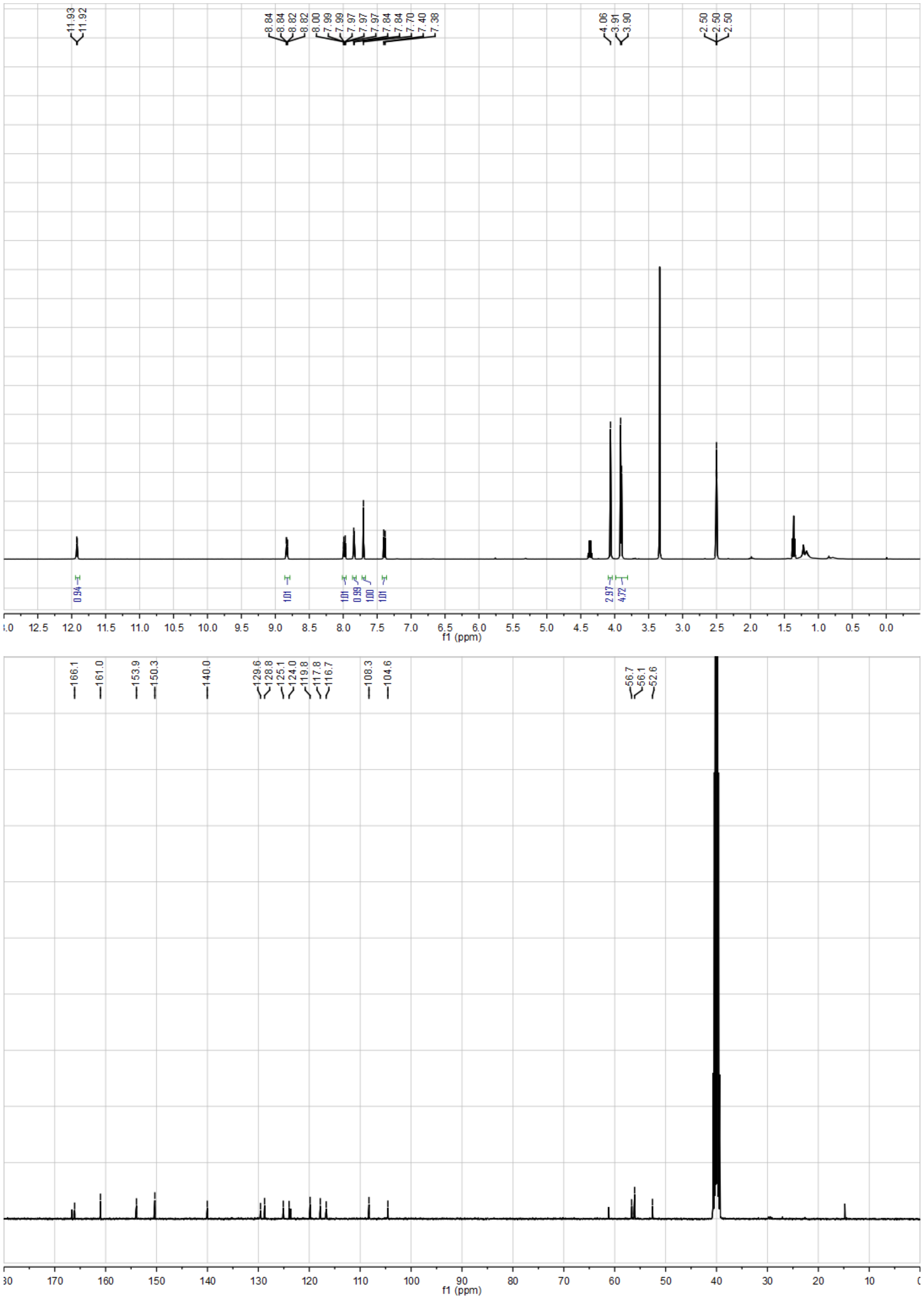

Fig. S40 NMR of compound 6d

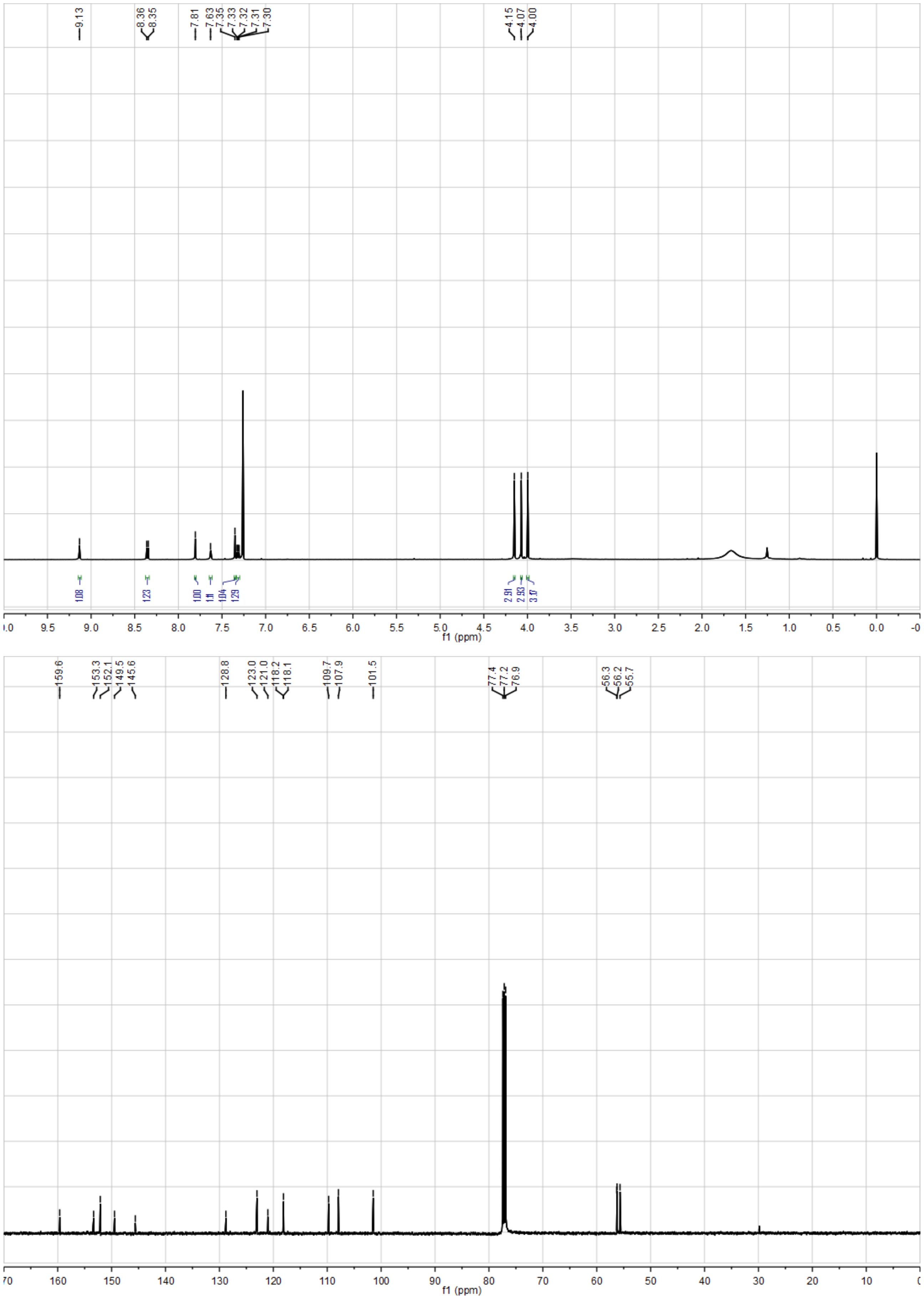

Fig. S41 NMR of compound 7a

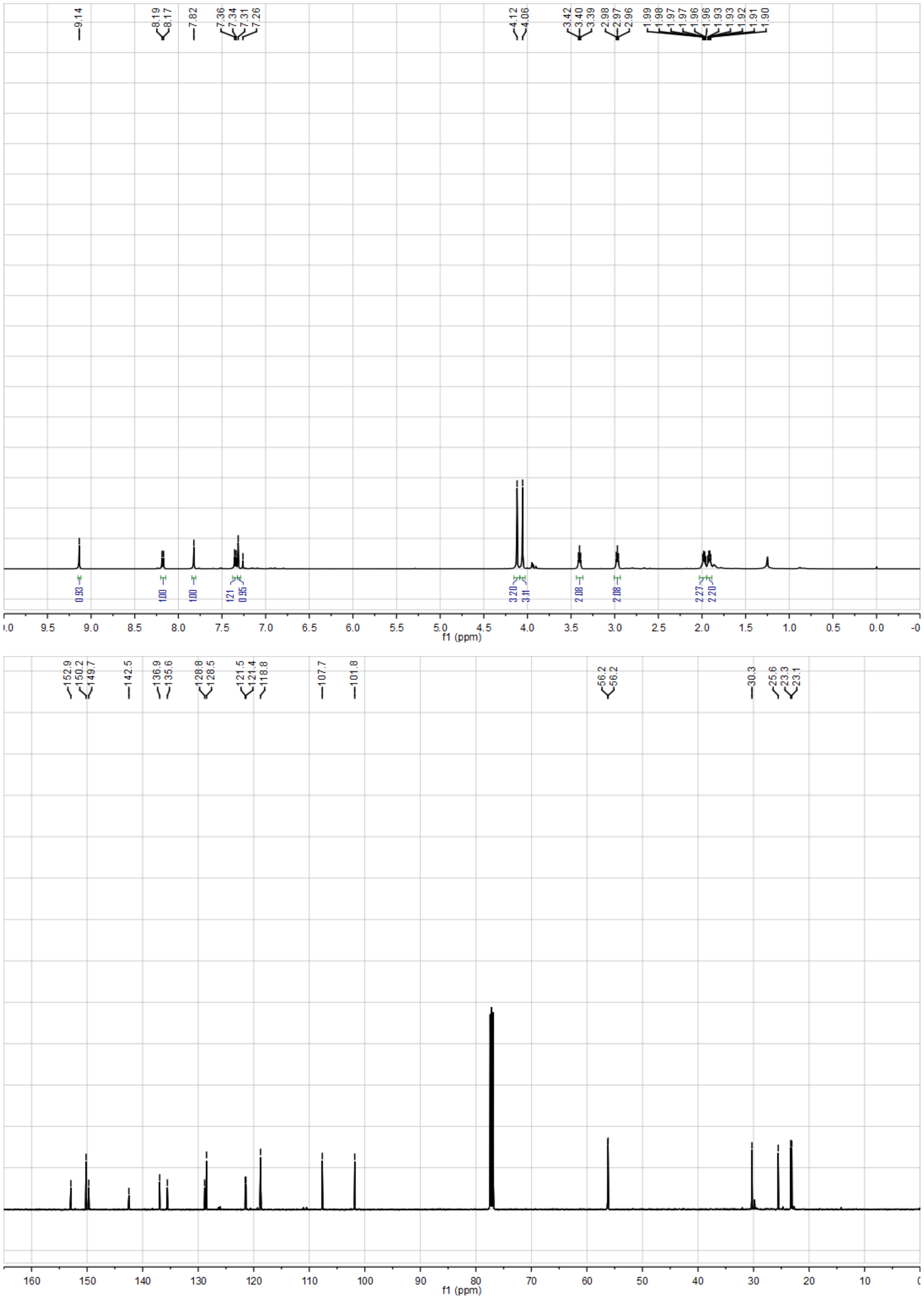

Fig. S42 NMR of compound 7b

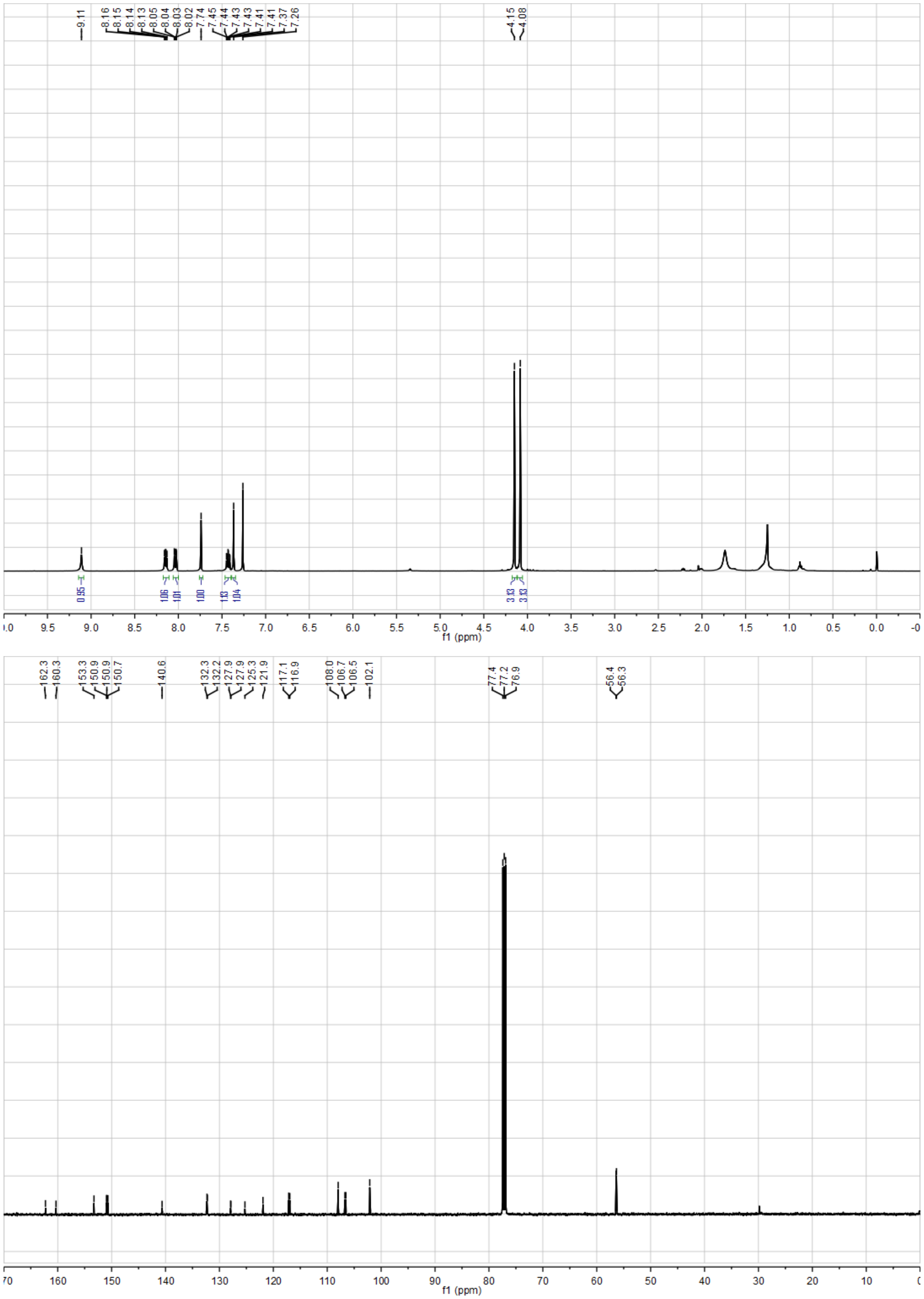

Fig. S43 NMR of compound 7c

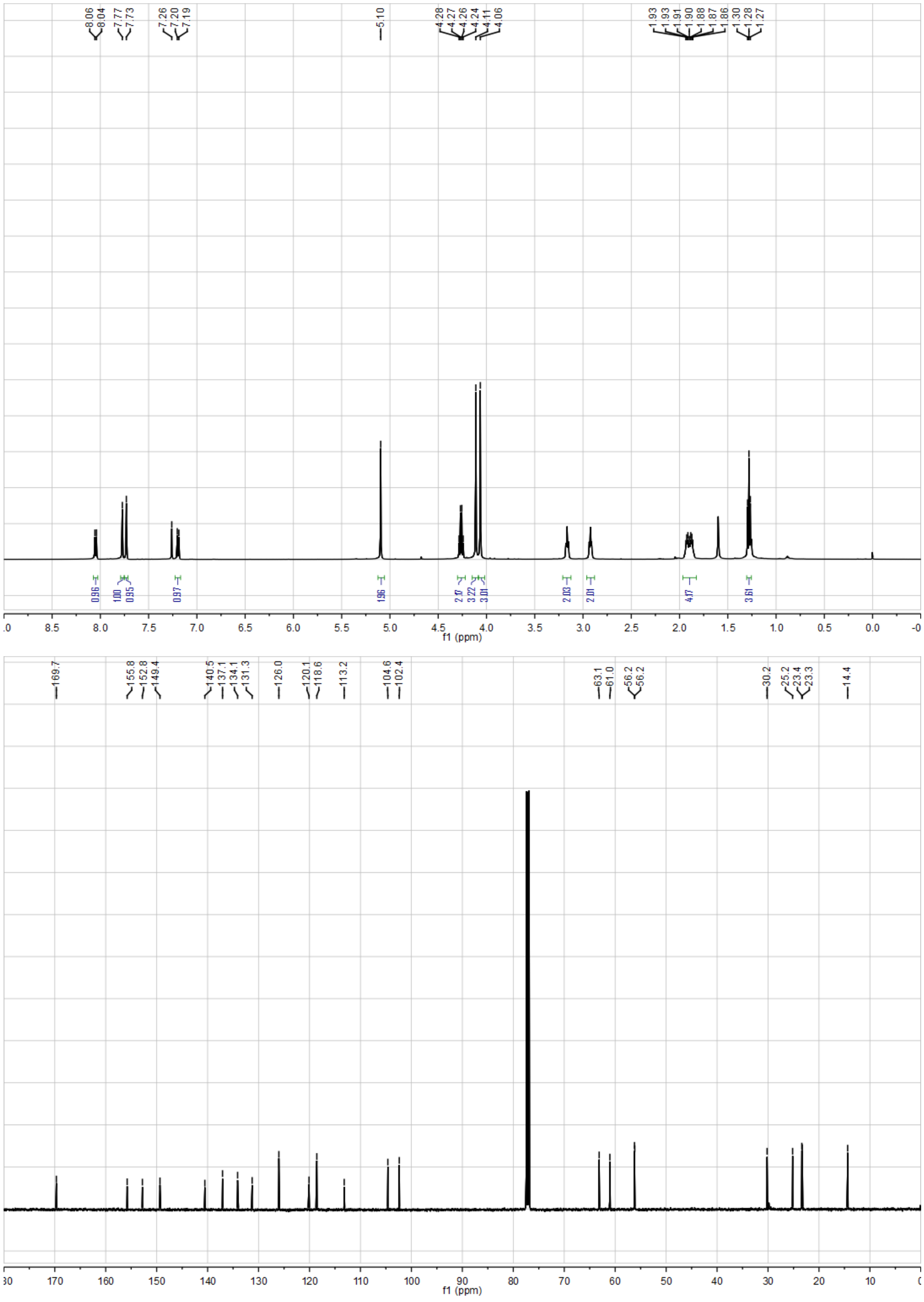

Fig. S44 NMR of compound 8a

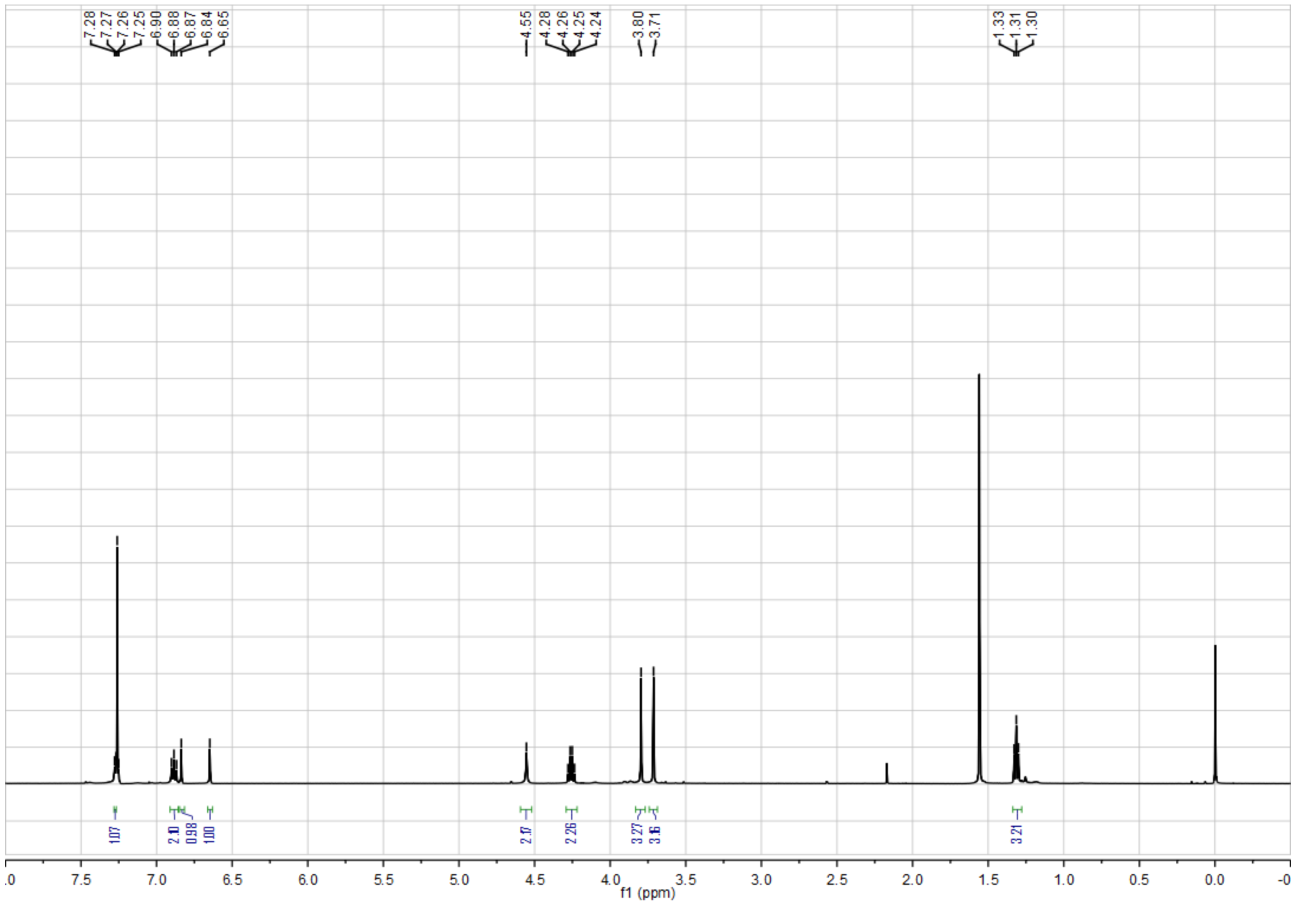

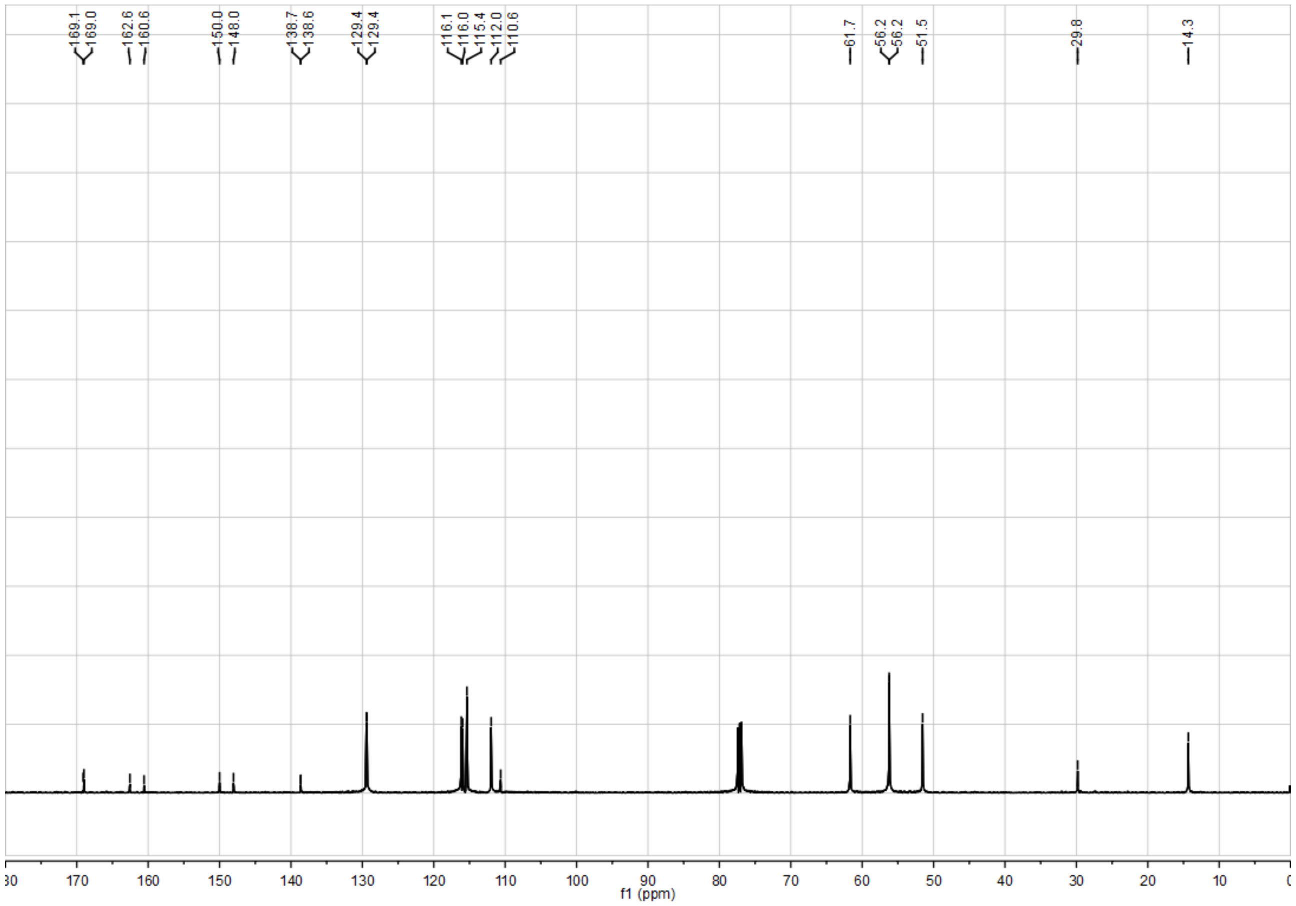

Fig. S45 NMR of compound 8b

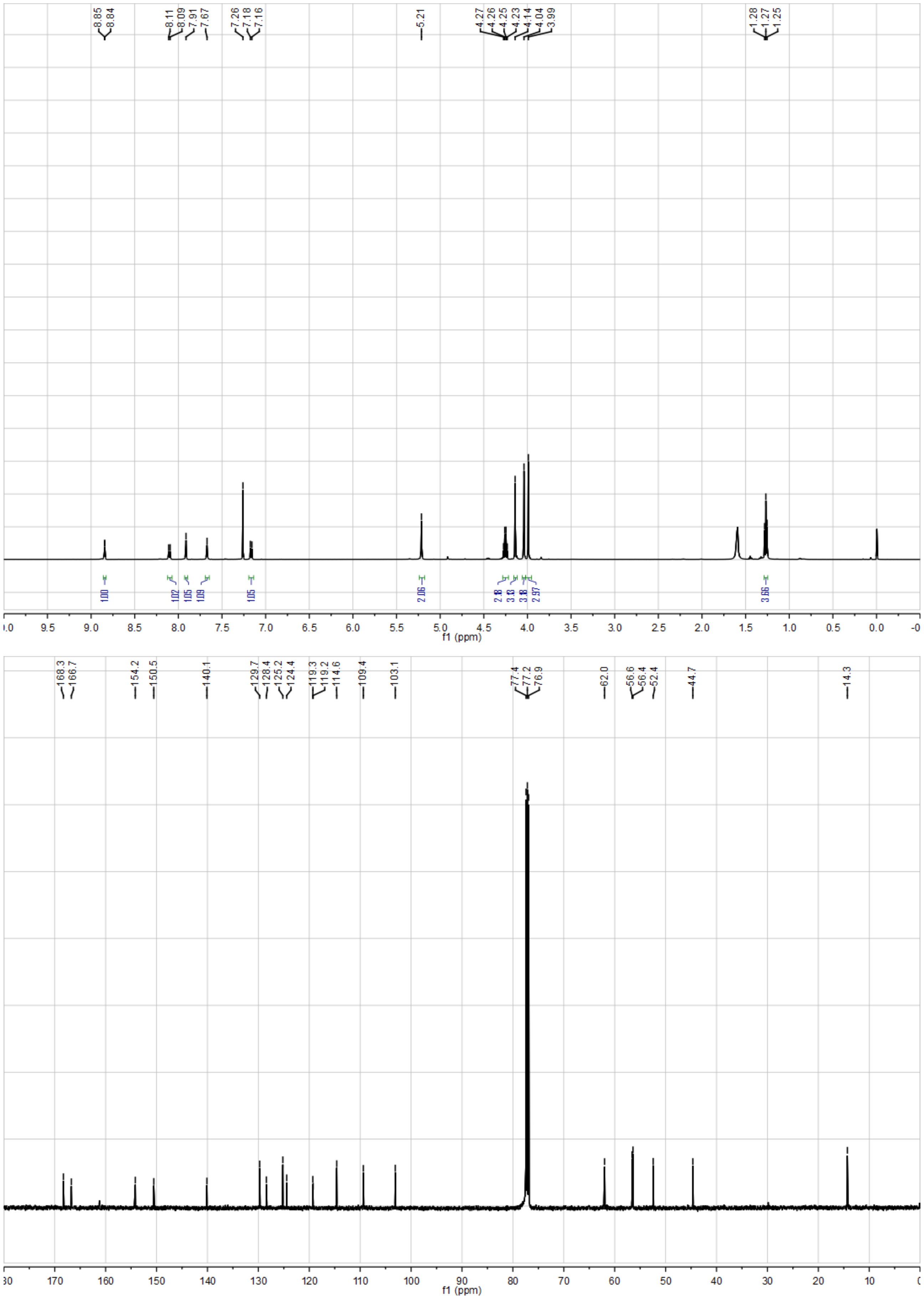

Fig. S46 NMR of compound 8c

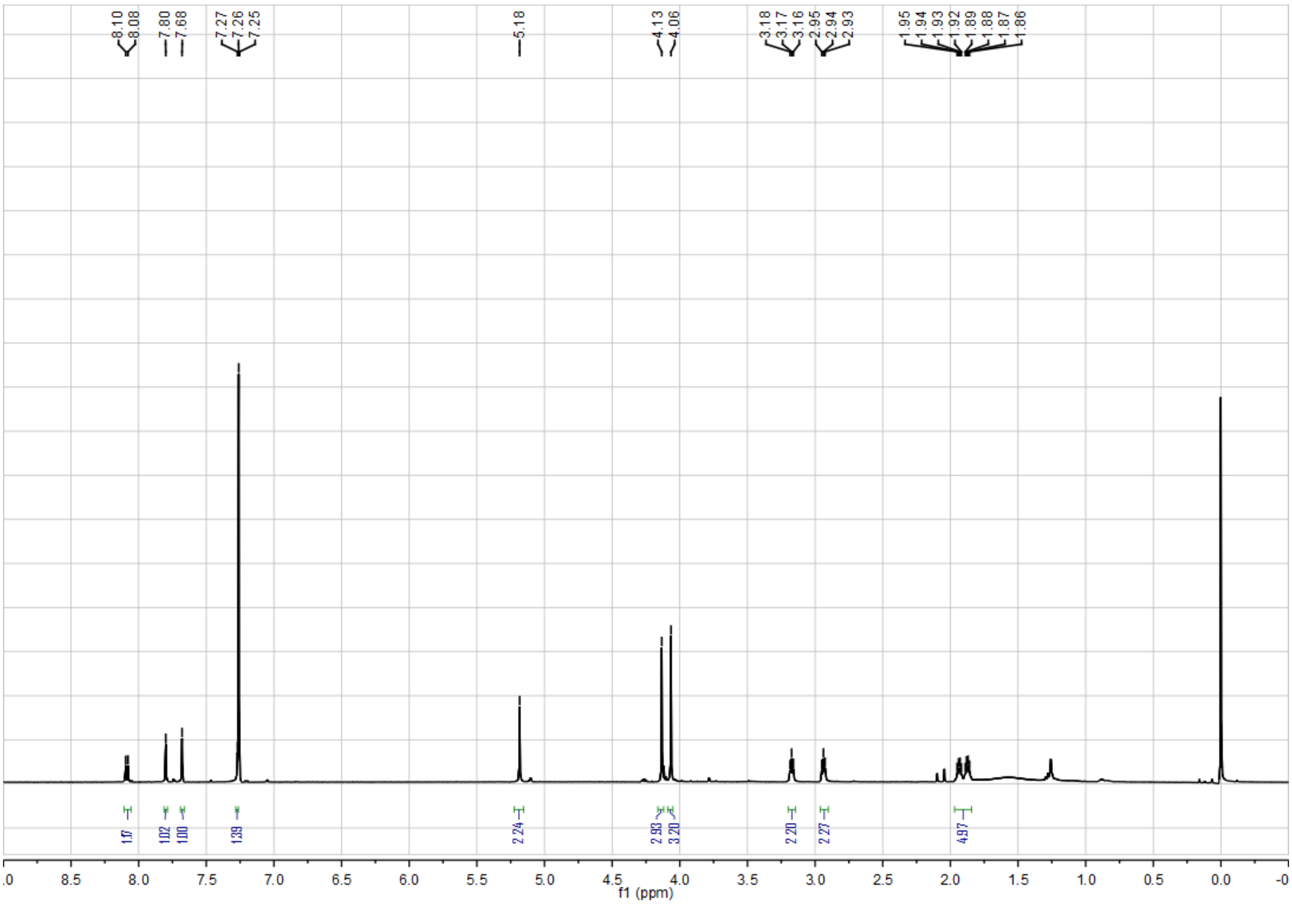

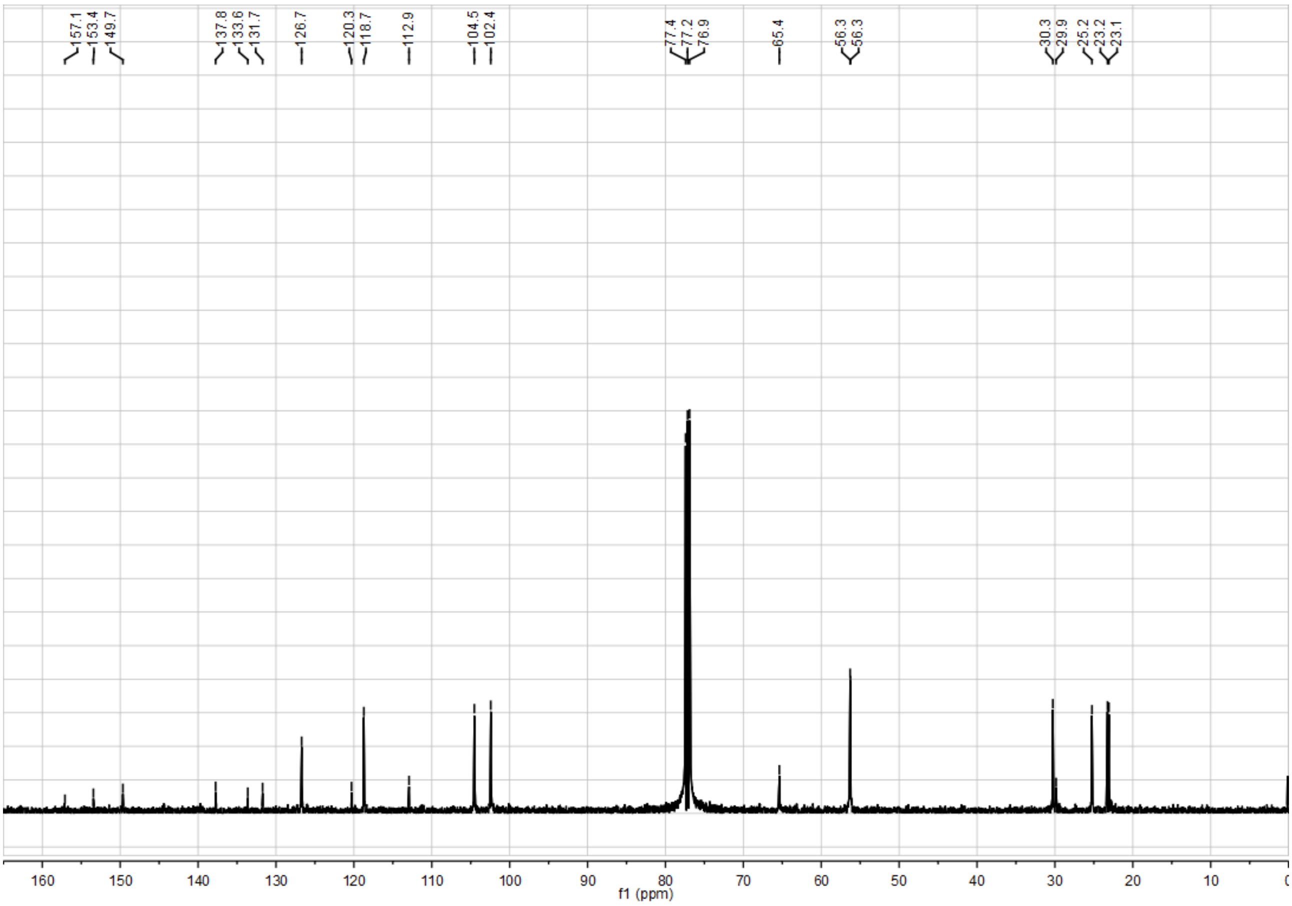

Fig. S47 NMR of compound 9a

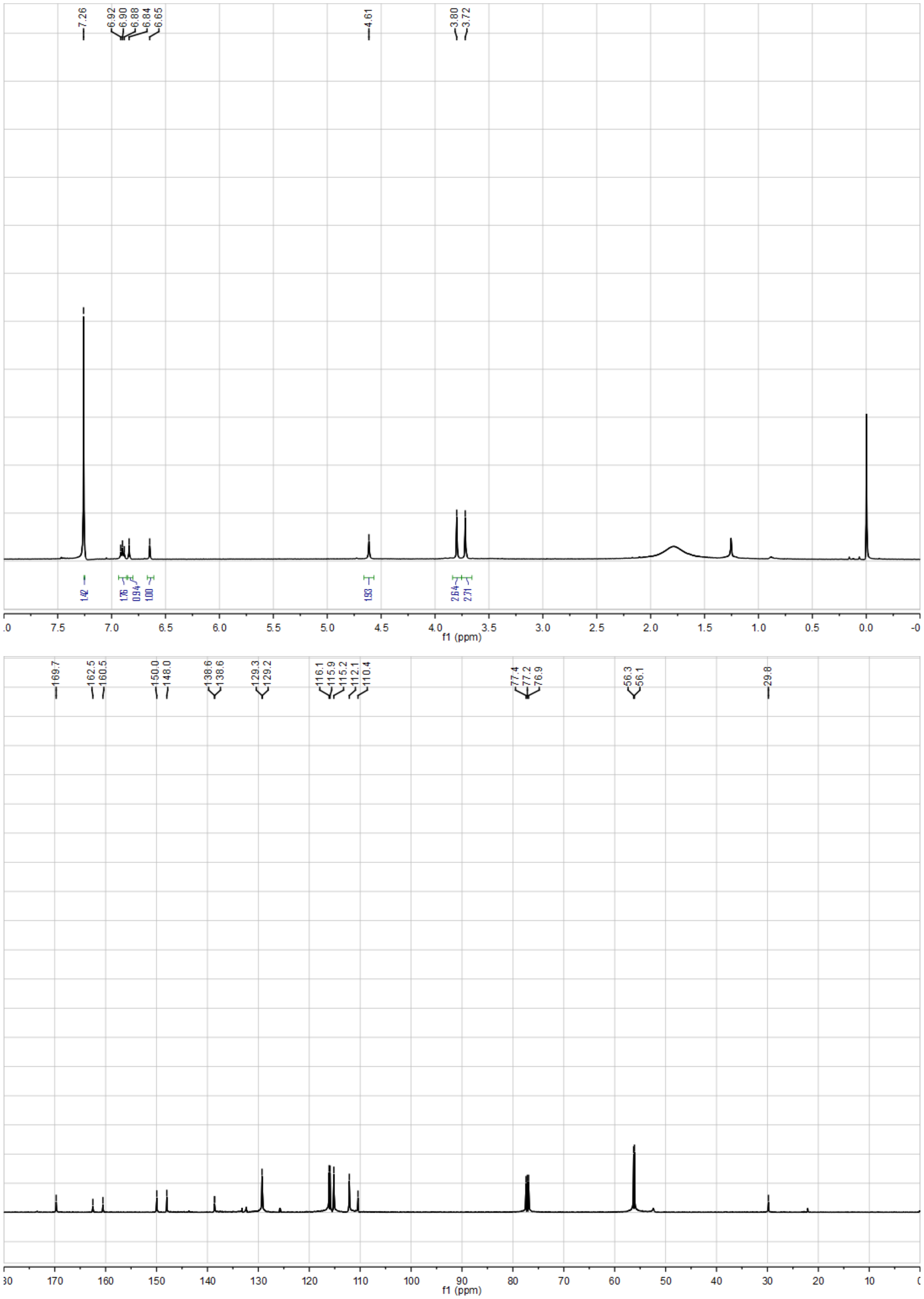

Fig. S48 NMR of compound 9b

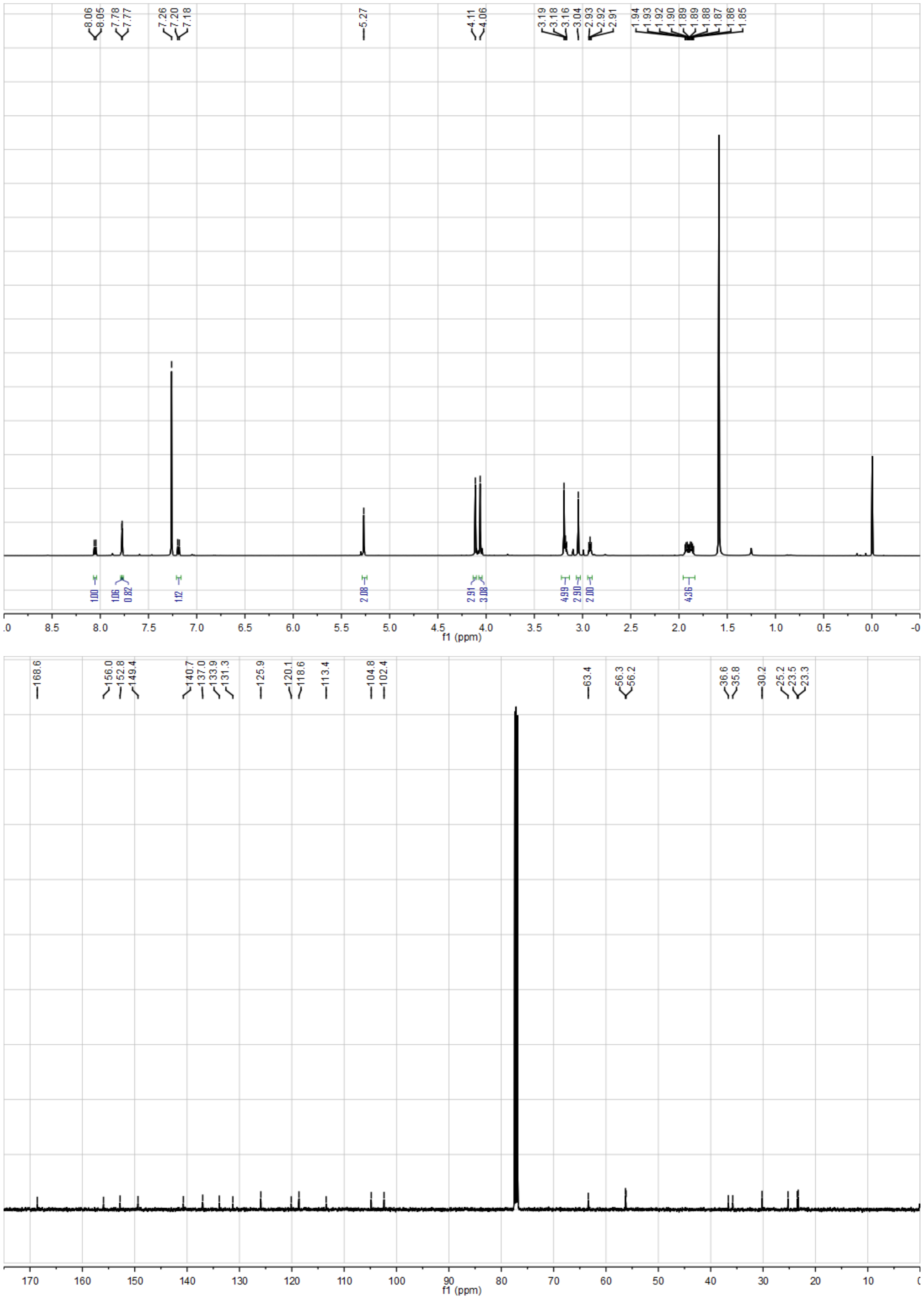

Fig. S49 NMR of compound 10a

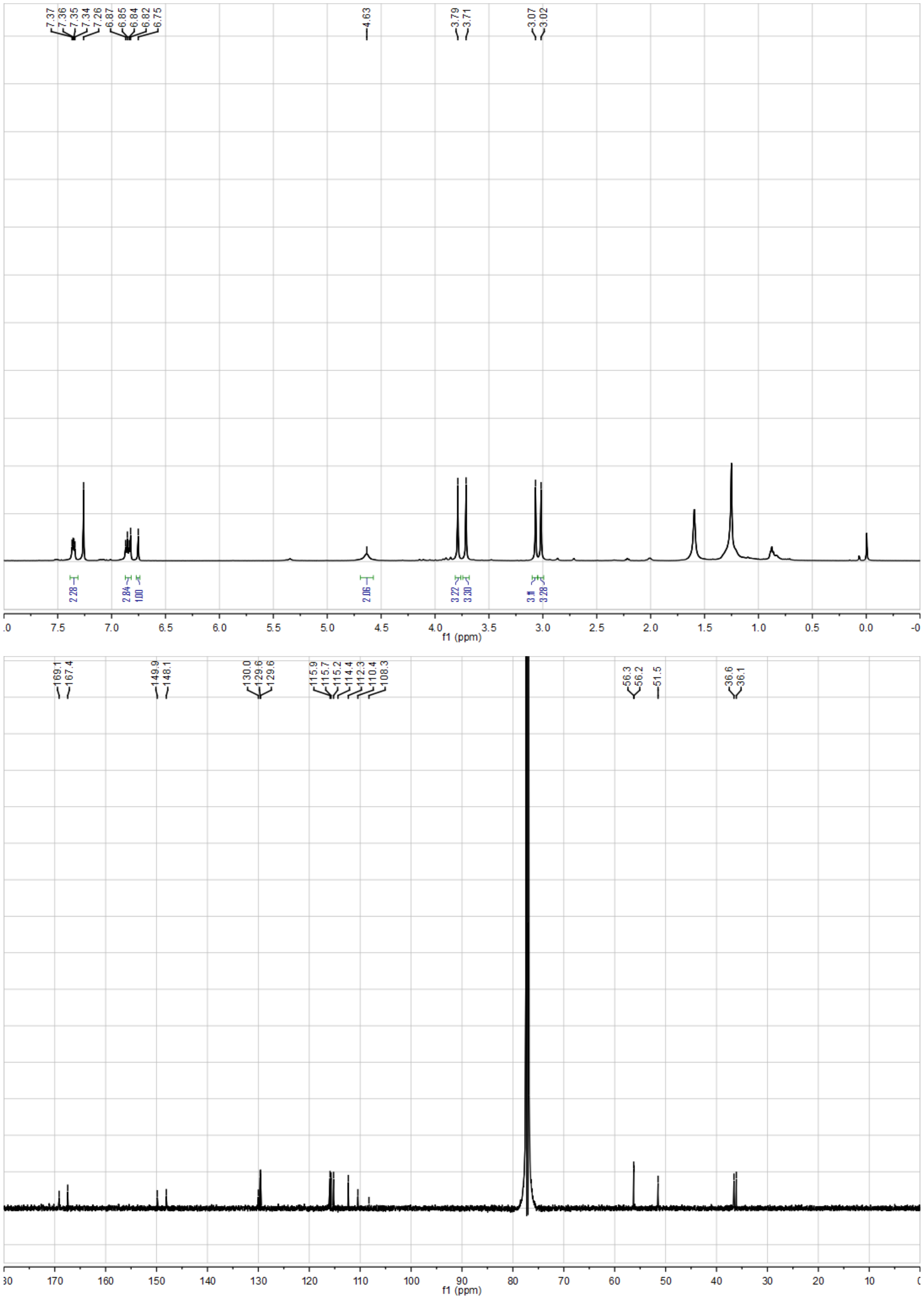

Fig. S50 NMR of compound 10b

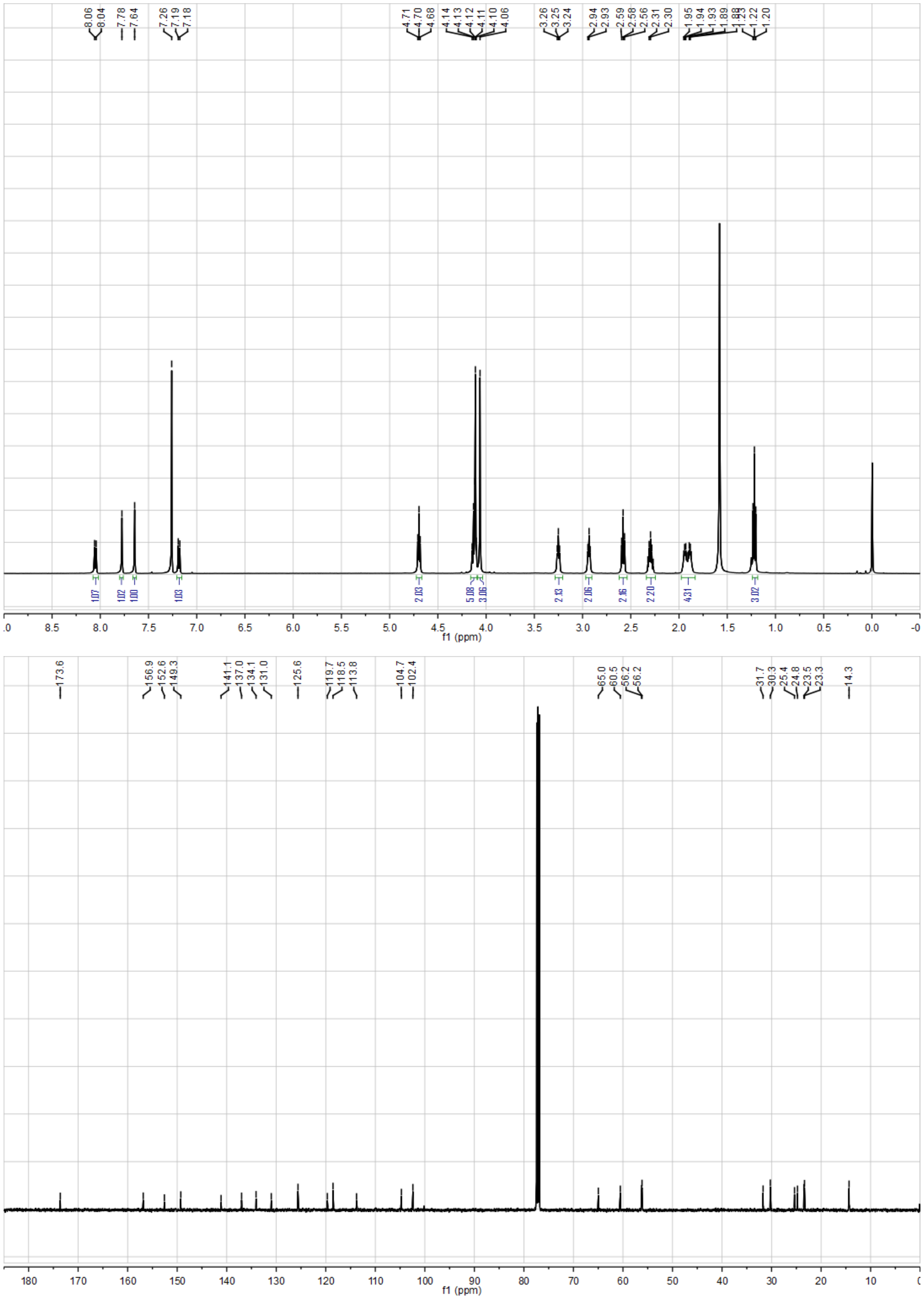

Fig. S51 NMR of compound 11a

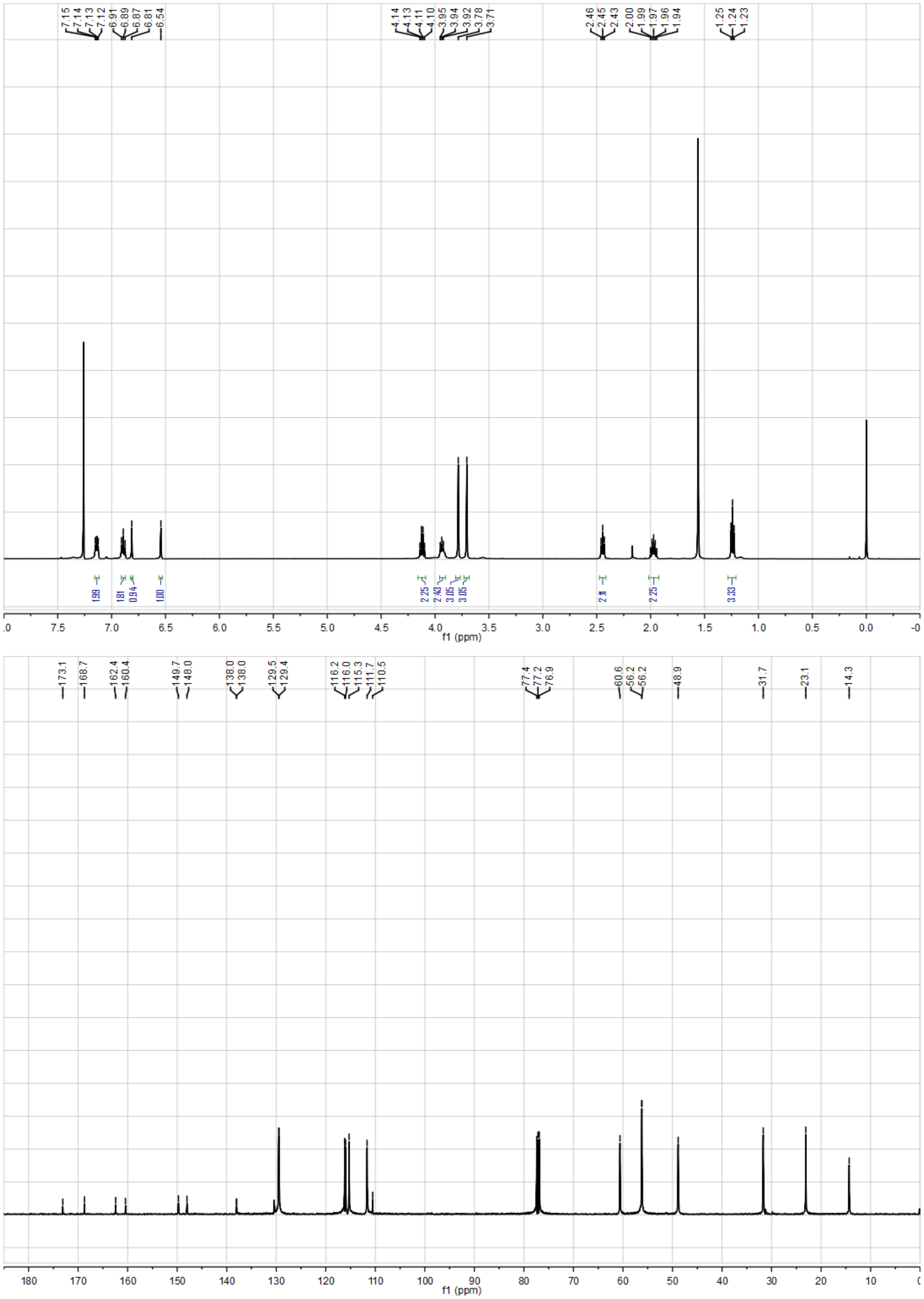

Fig. S52 NMR of compound 11b

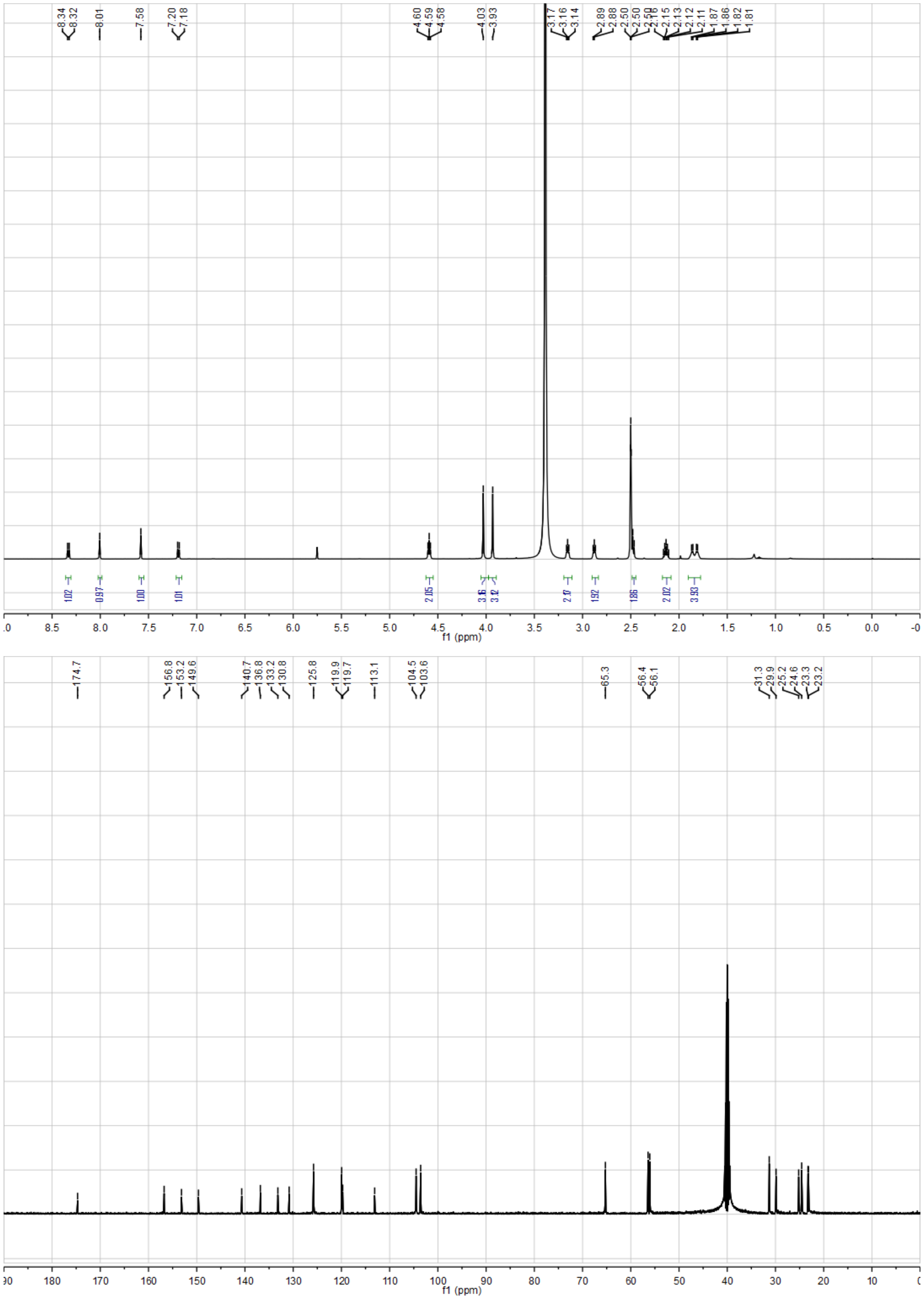

Fig. S53 NMR of compound 12a

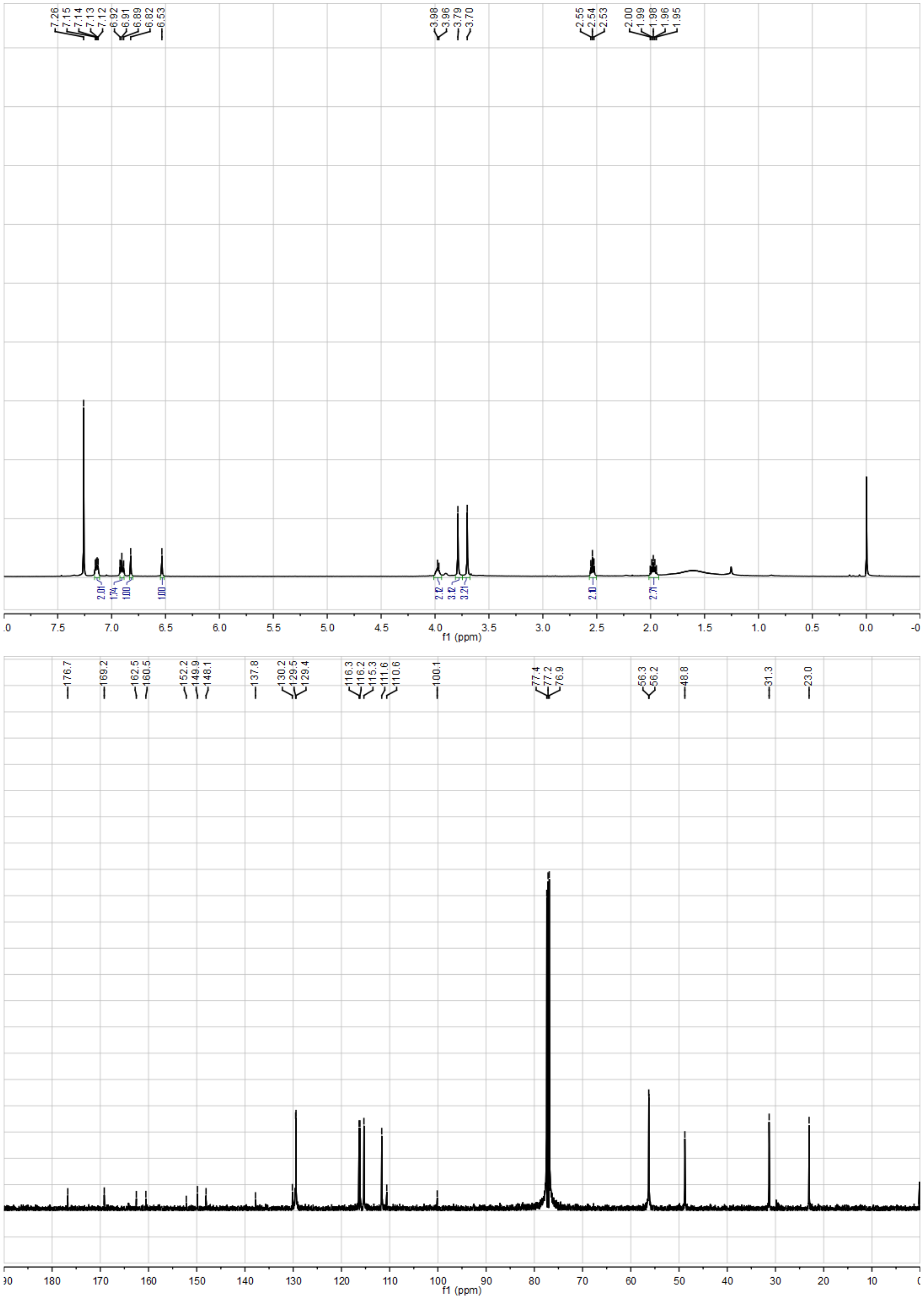

Fig. S54 NMR of compound 12b

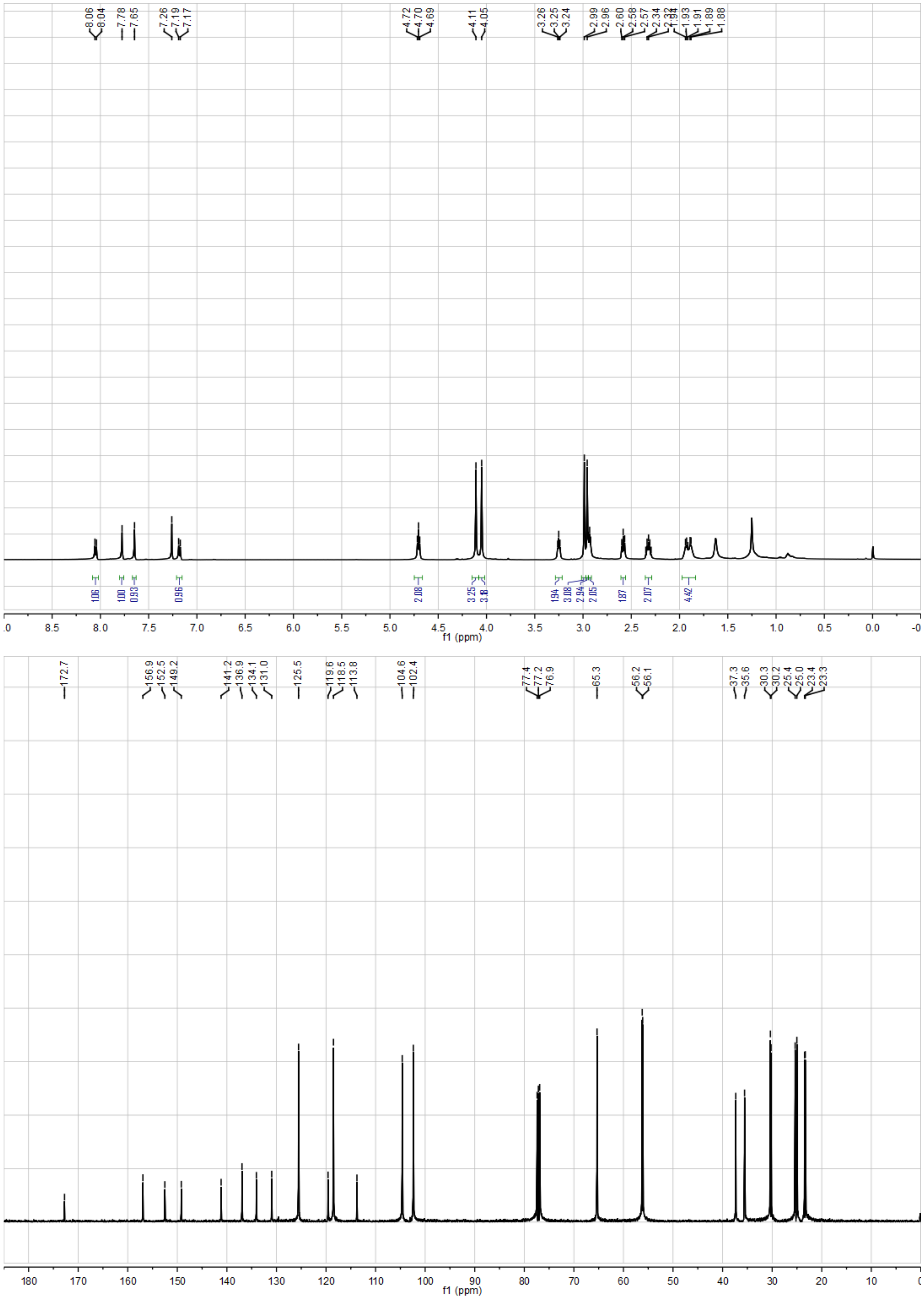

Fig. S55 NMR of compound 13a

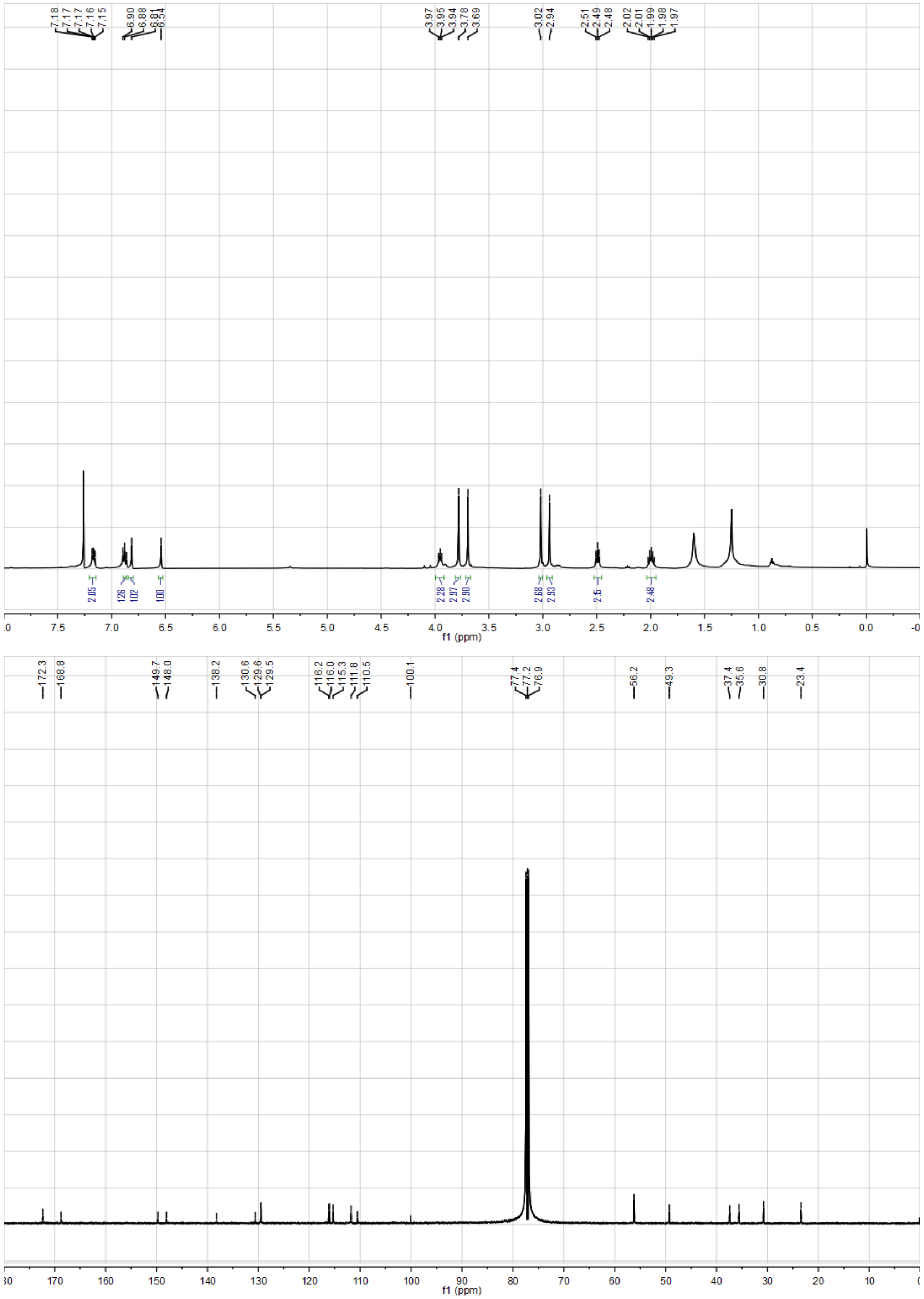

Fig. S56 NMR of compound 13b

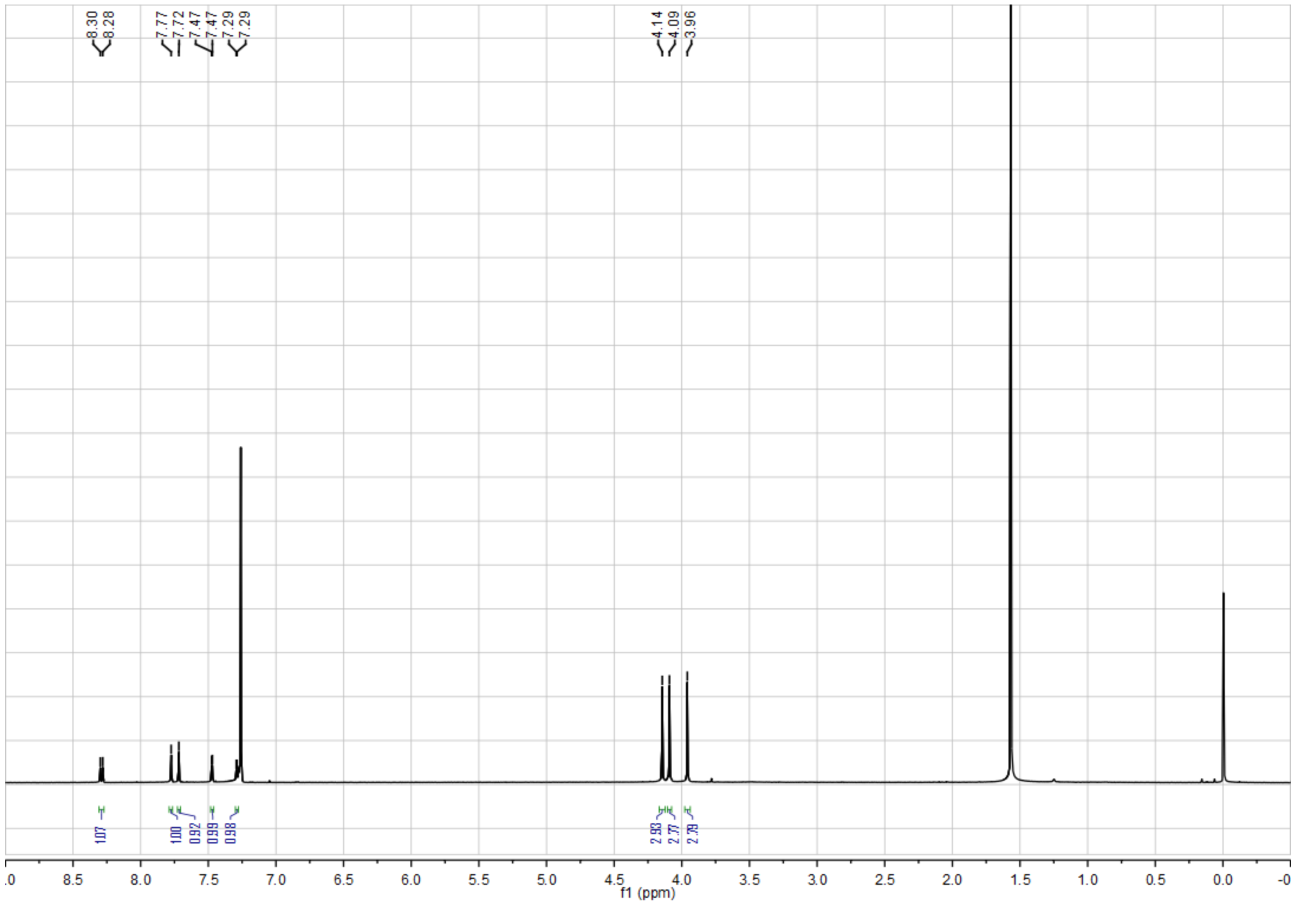

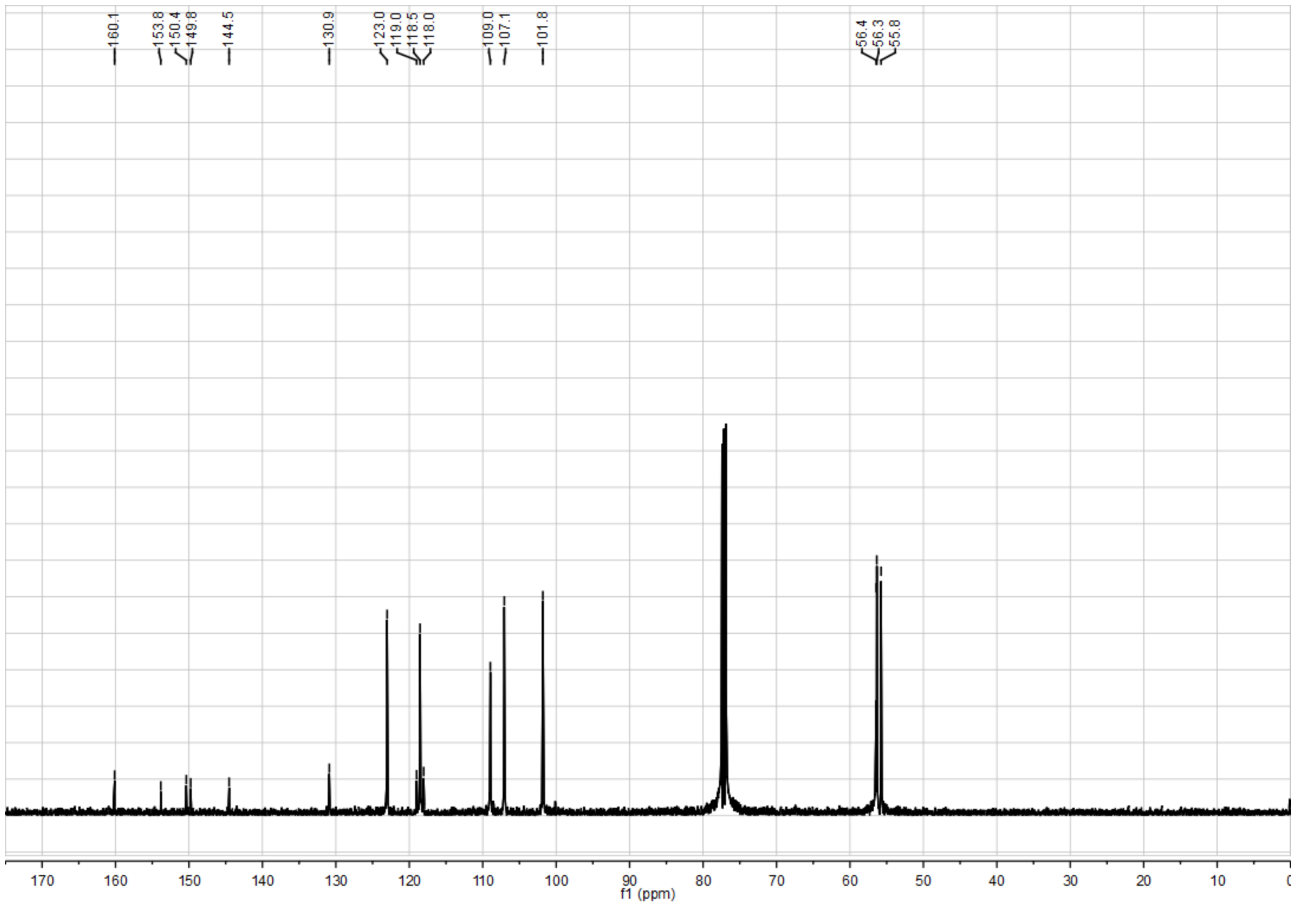

Fig. S57 NMR of compound 14a

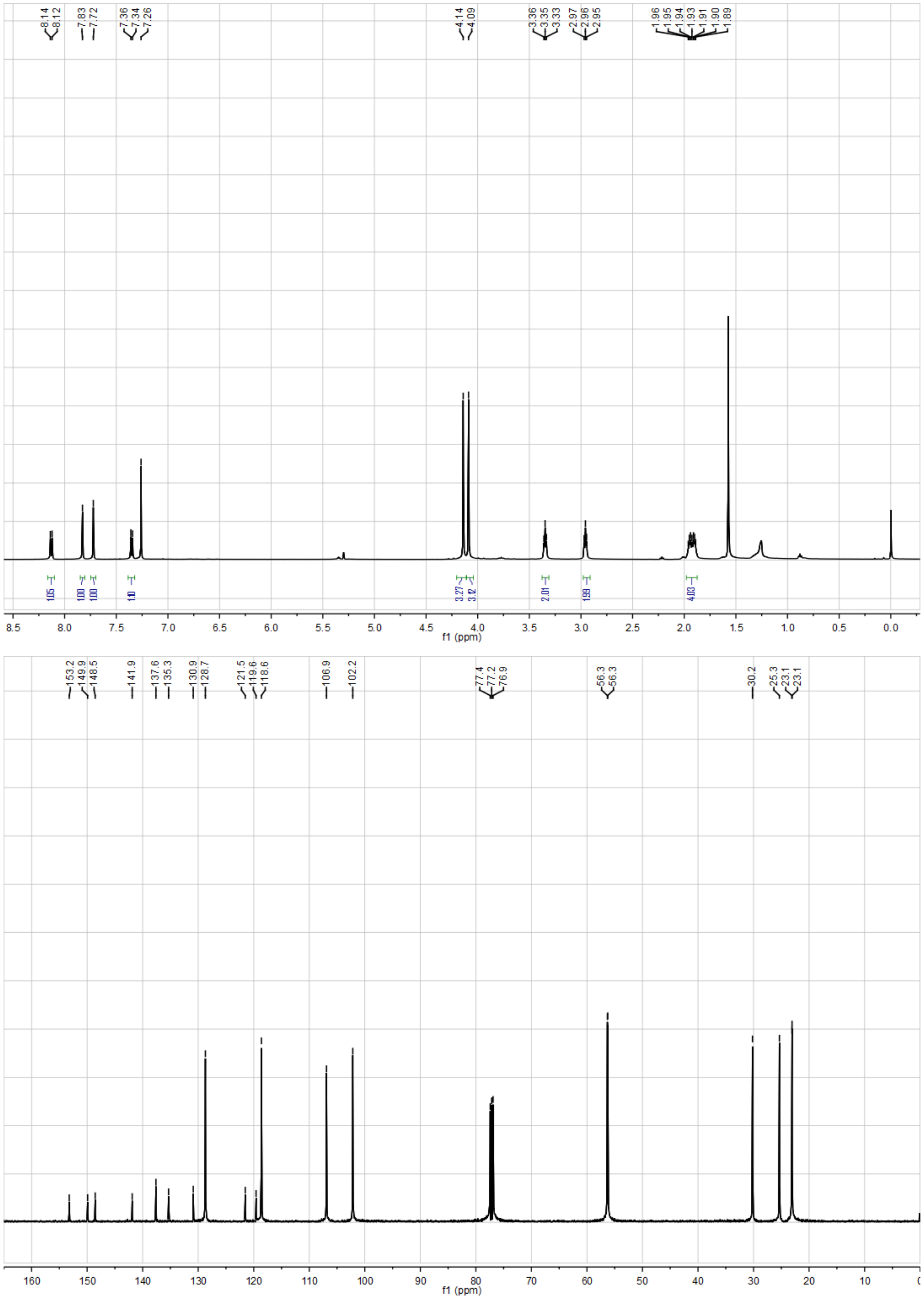

Fig. S58 NMR of compound 14b

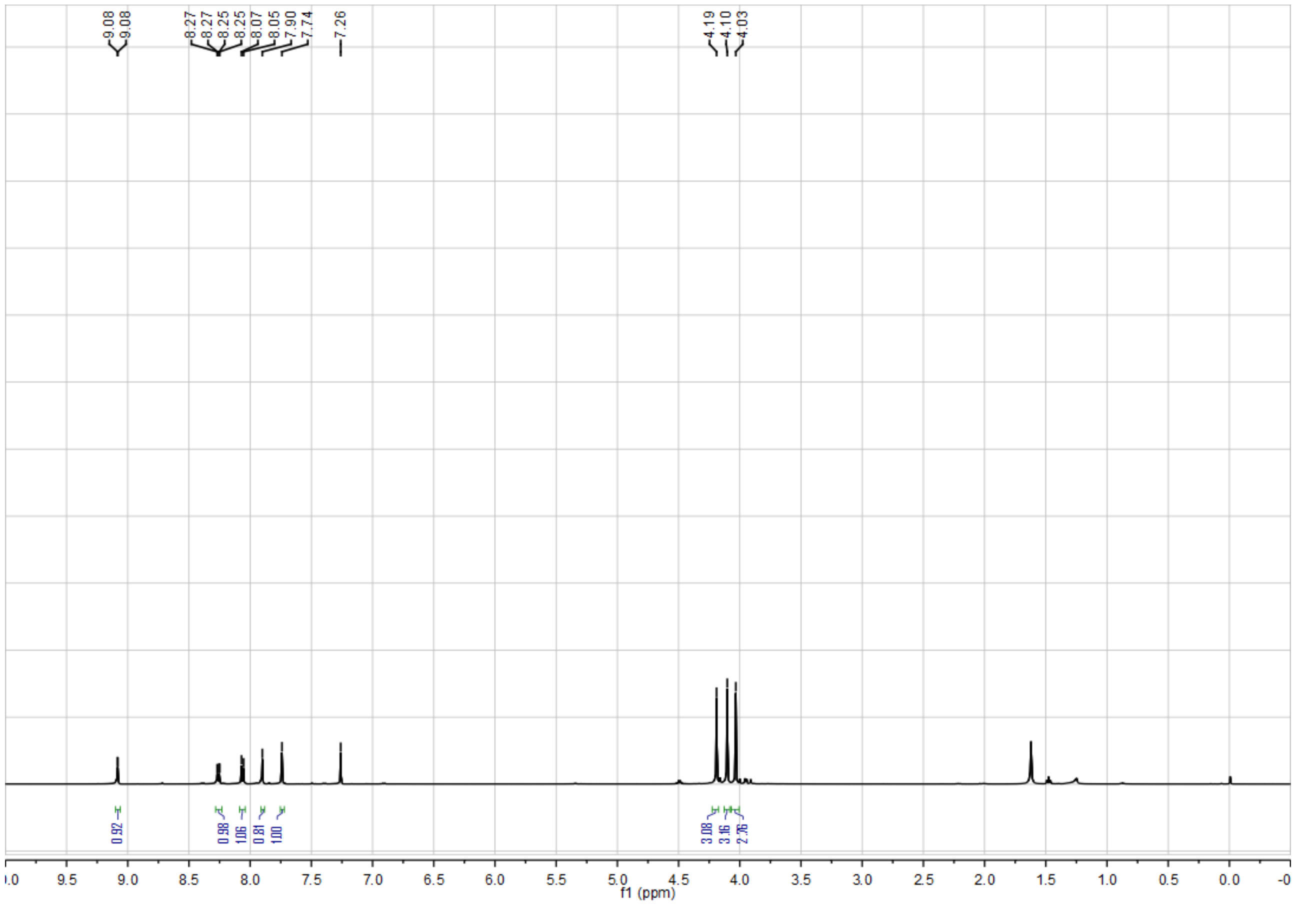

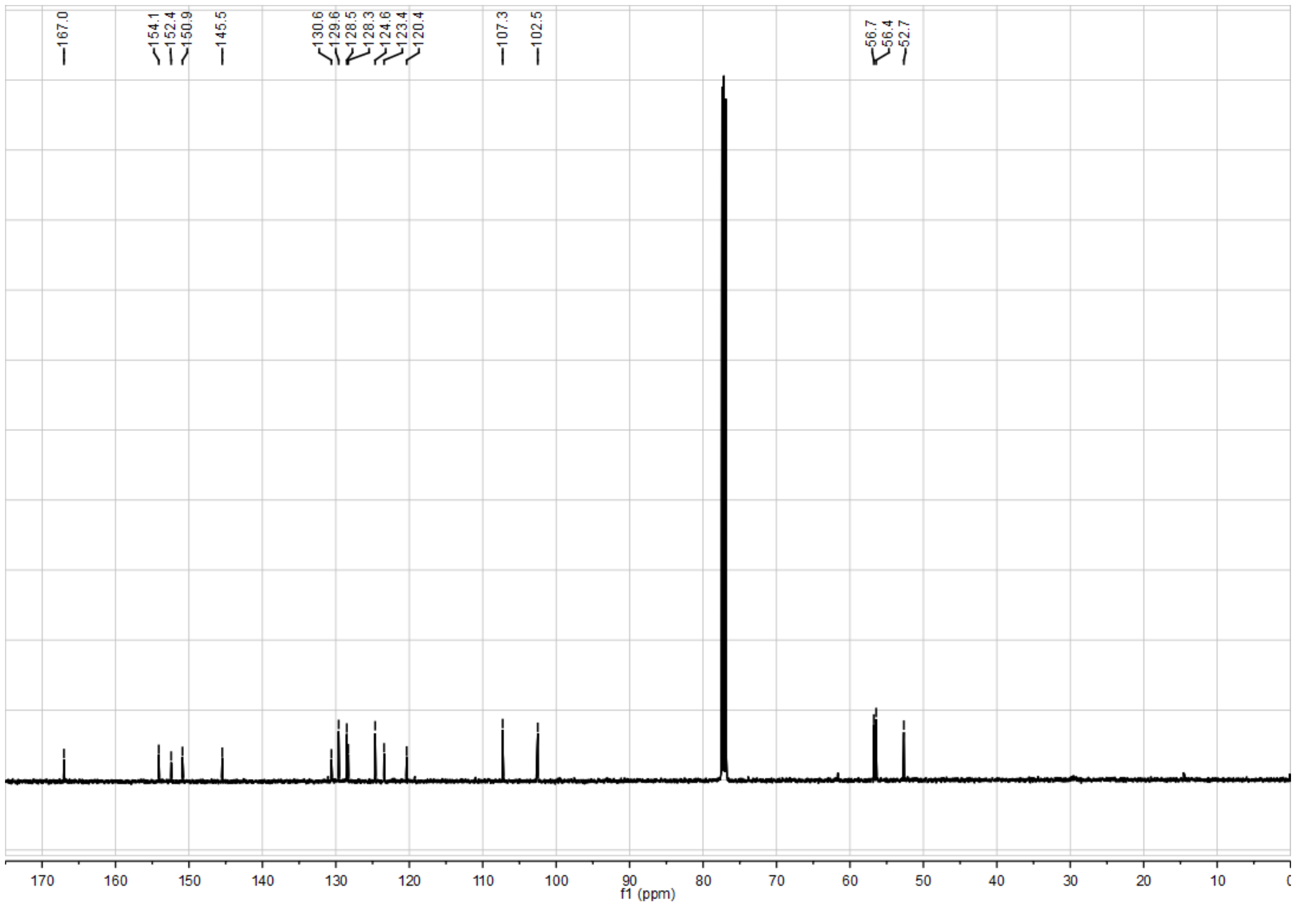

Fig. S59 NMR of compound 14c

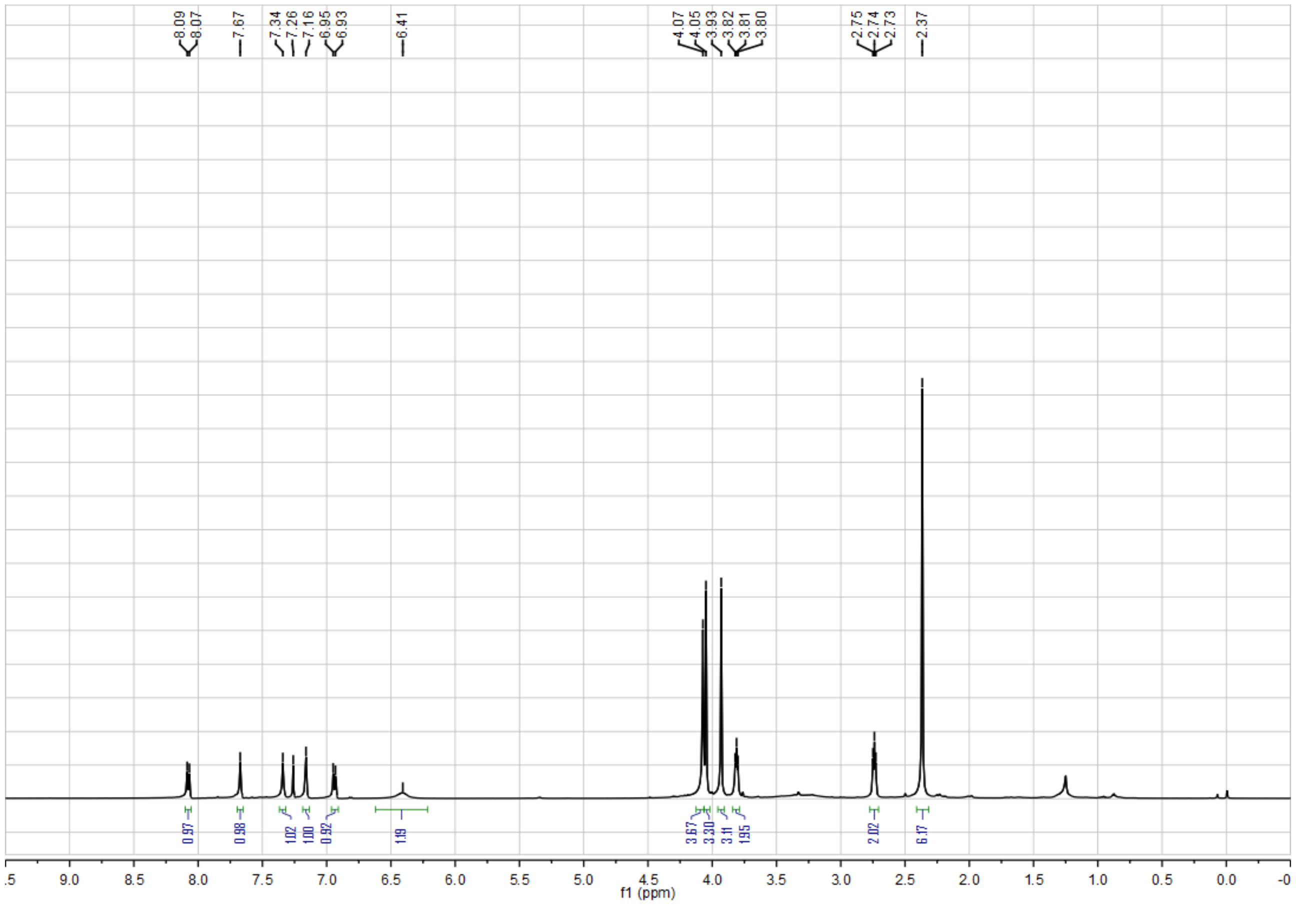

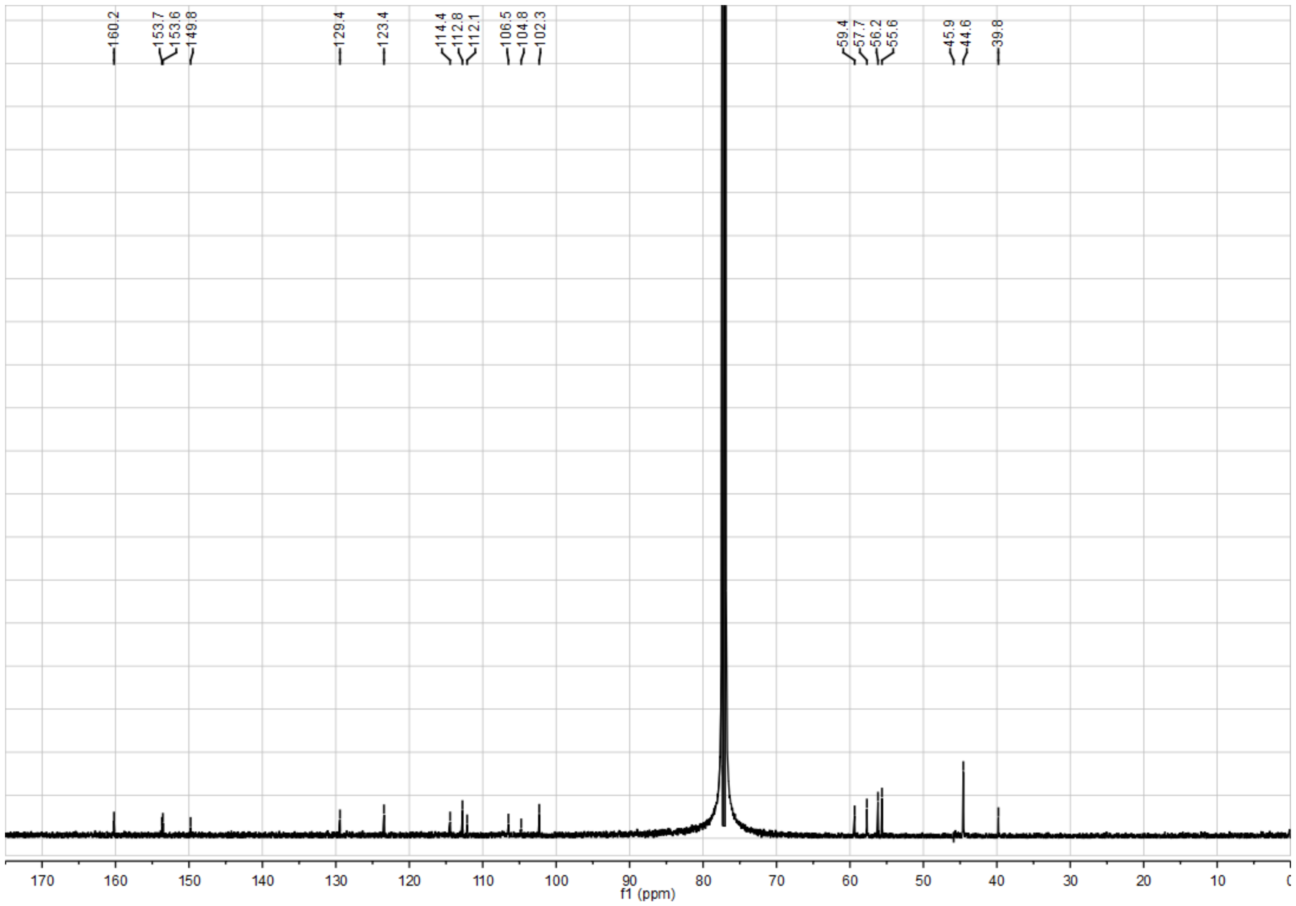

Fig. S60 NMR of compound 15a

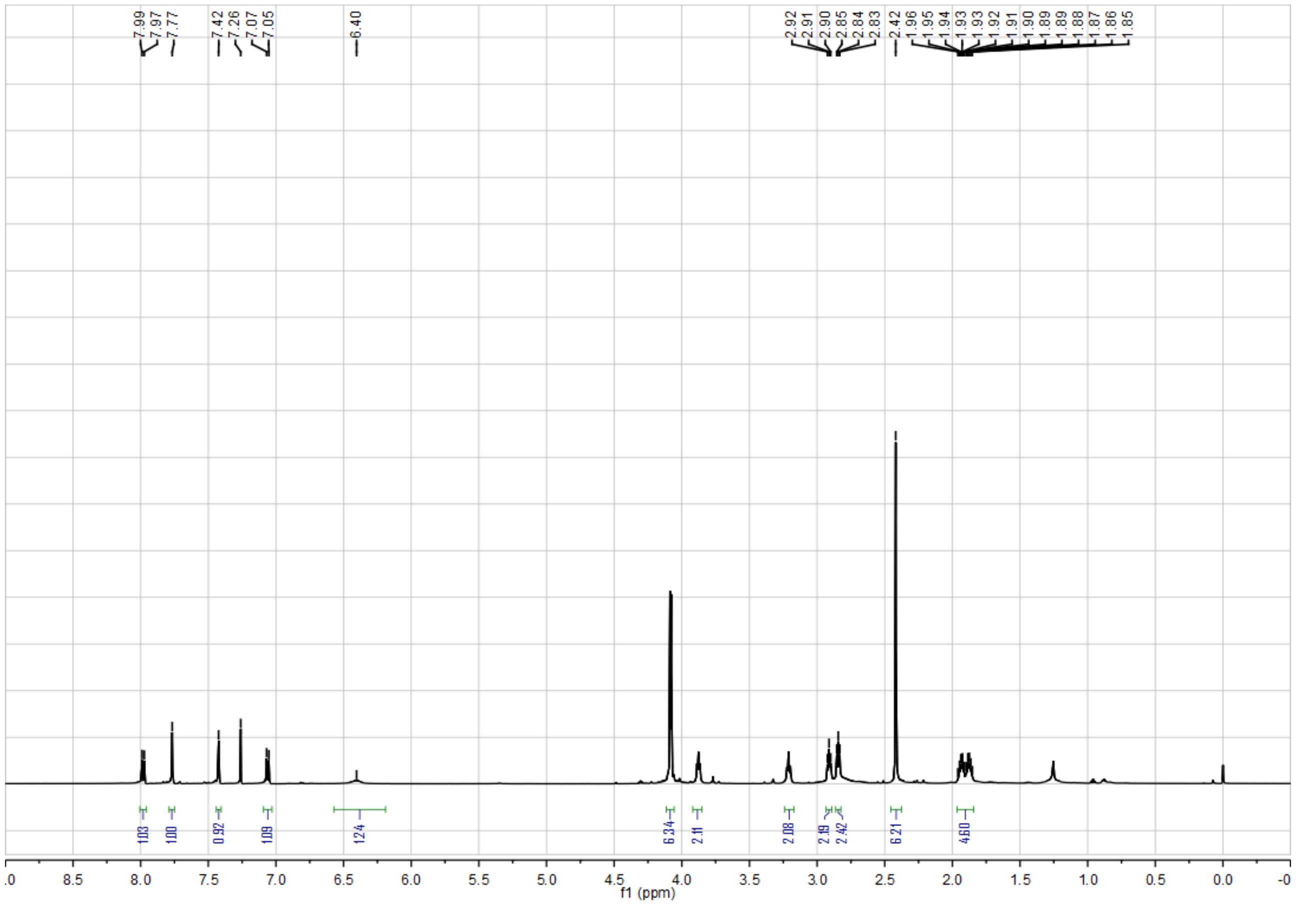

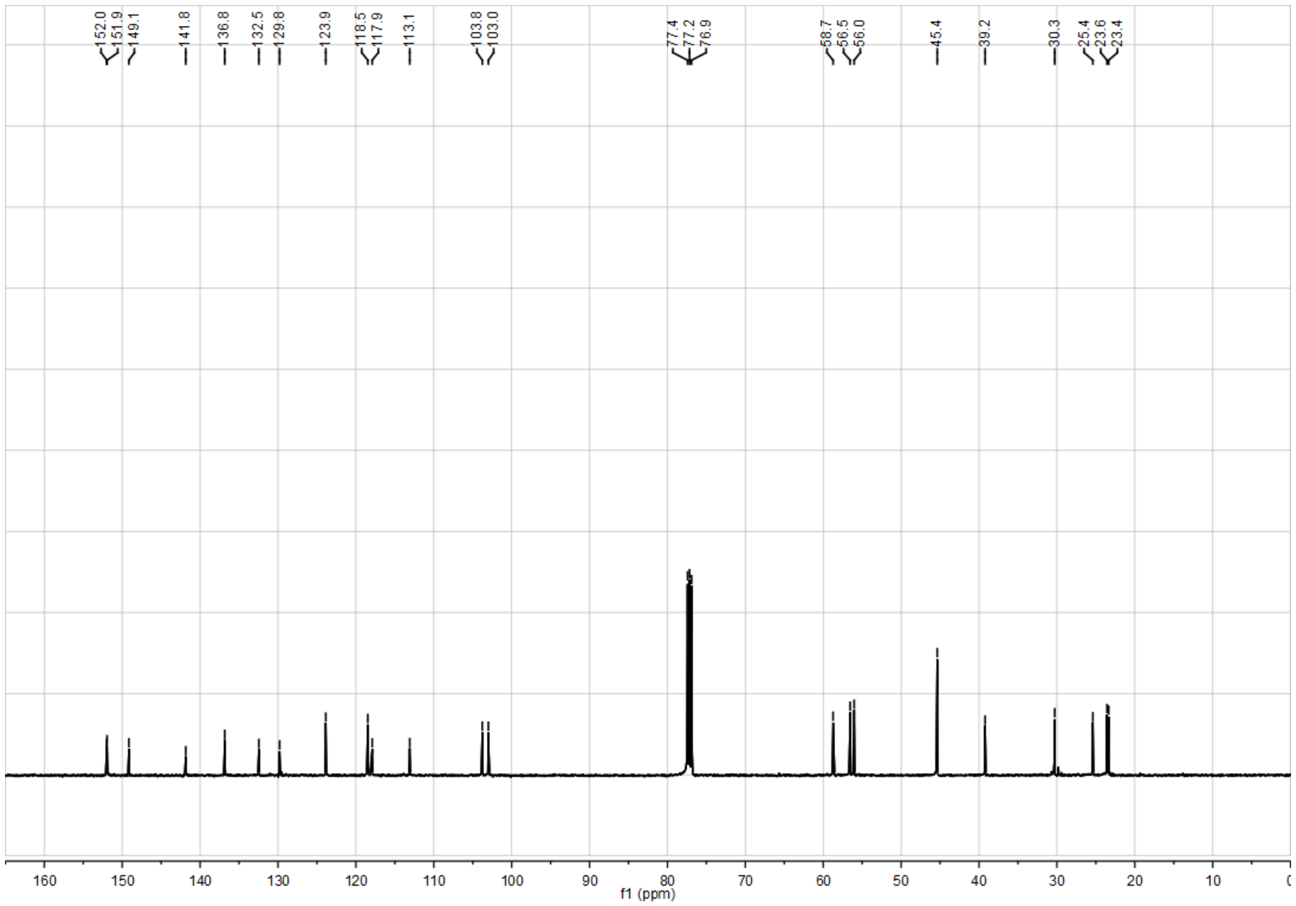

Fig. S61 NMR of compound 15b

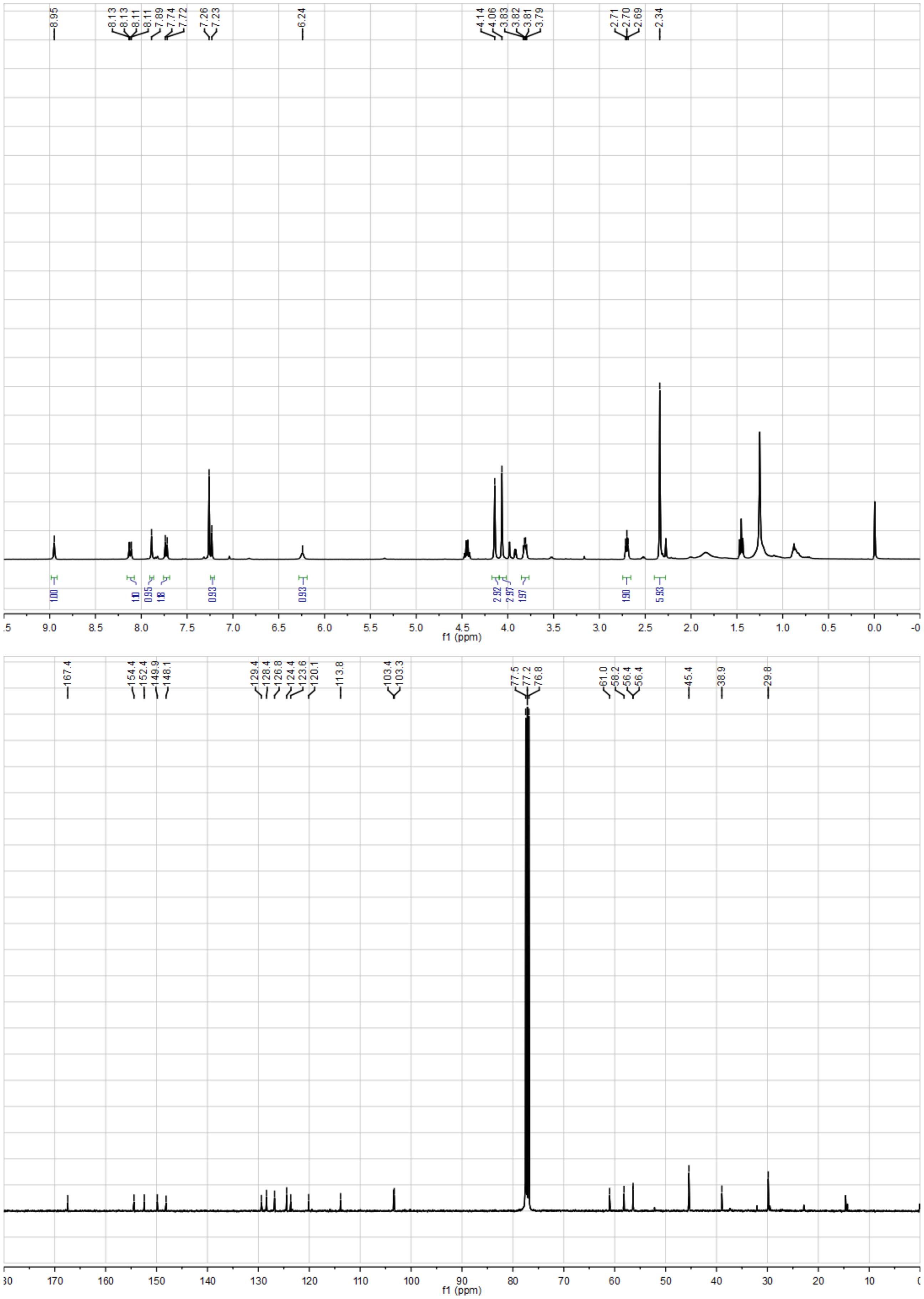

Fig. S62 NMR of compound 15c

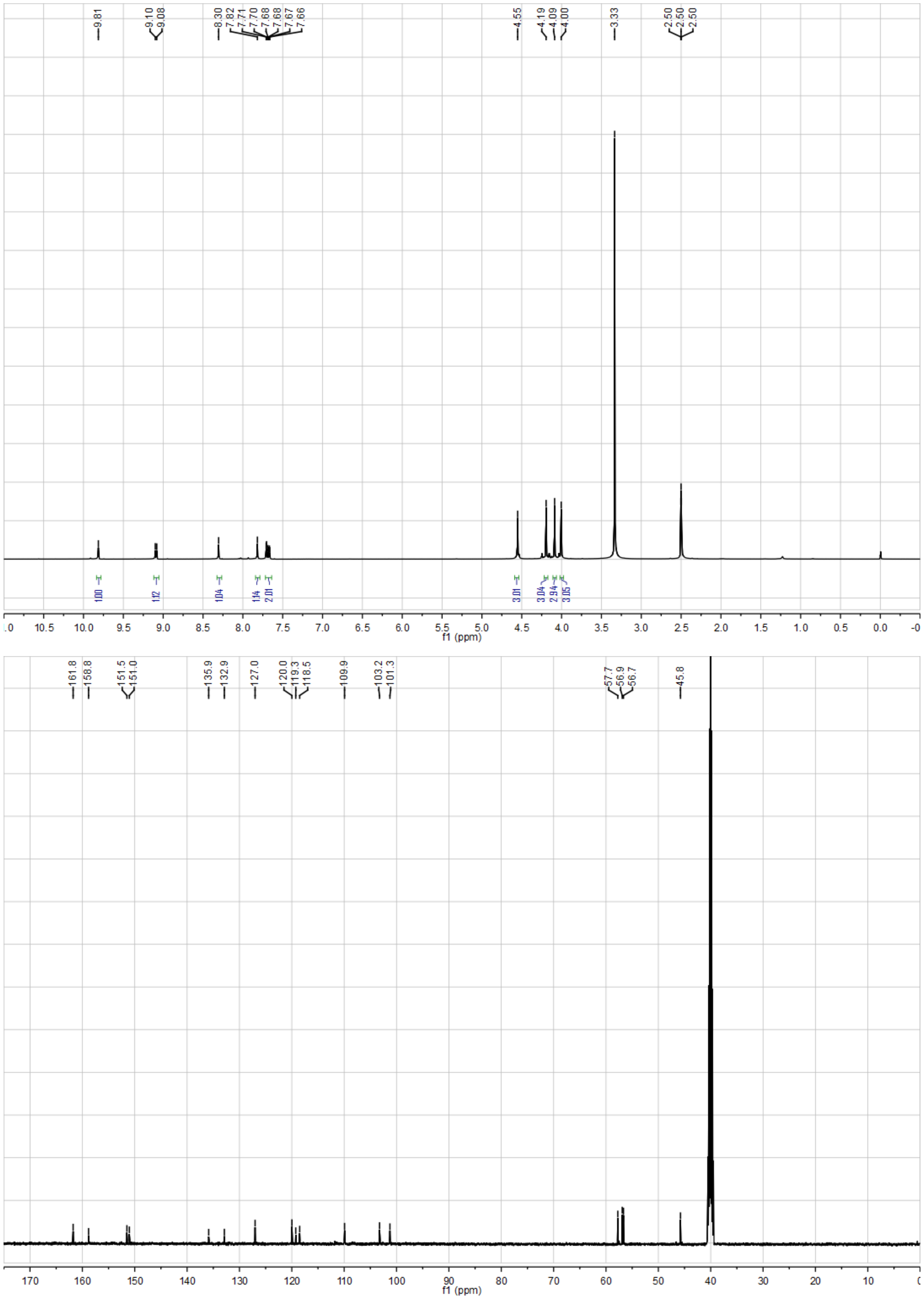

Fig. S63 NMR of compound 16a

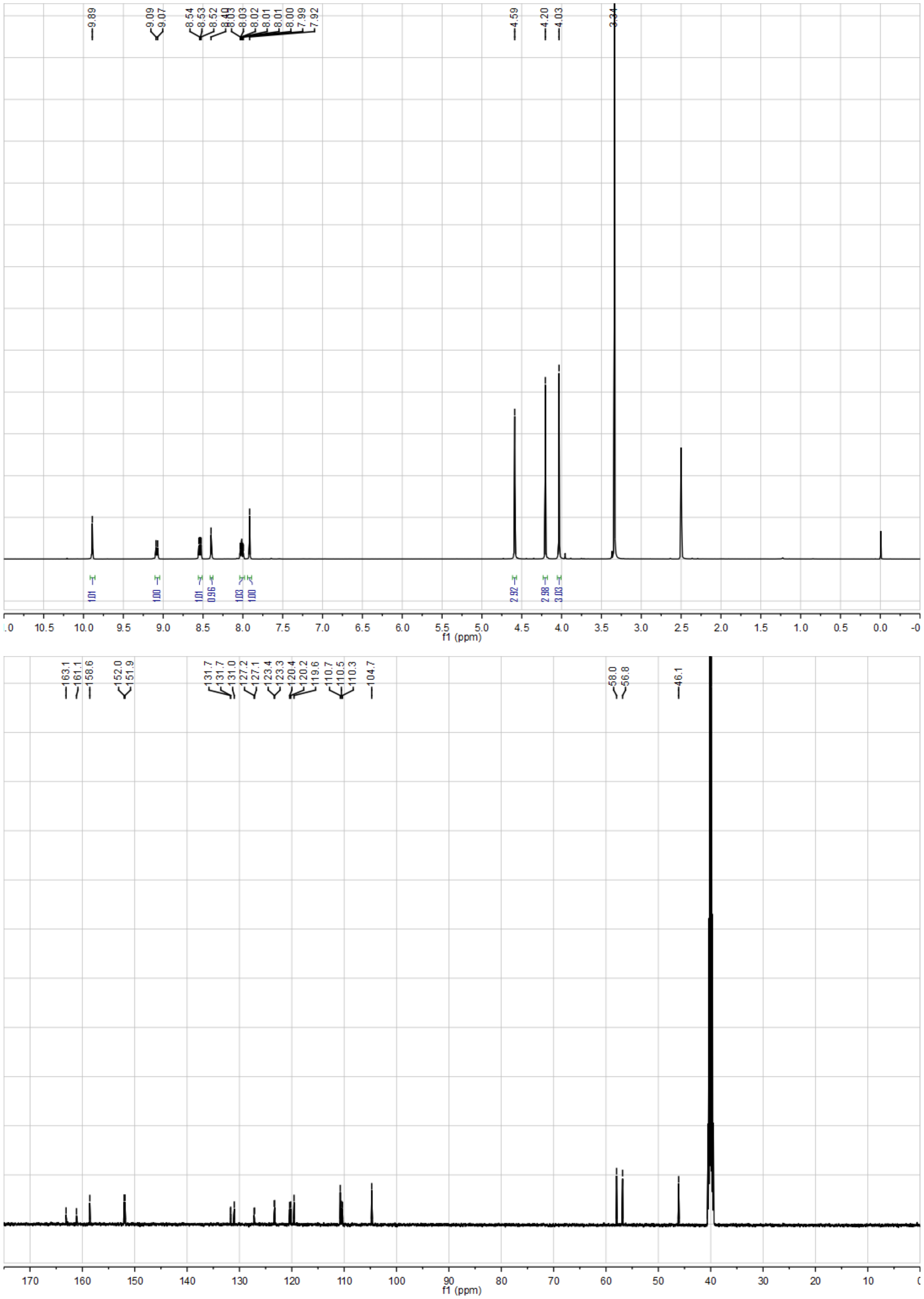

Fig. S64 NMR of compound 16b

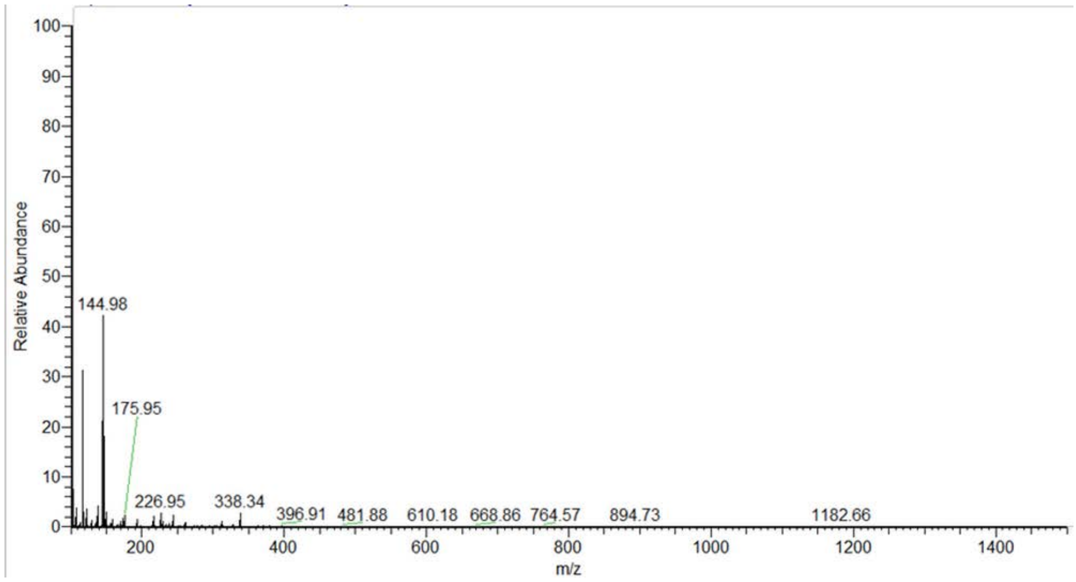

Fig. S65 ESI-MS of compound 5a

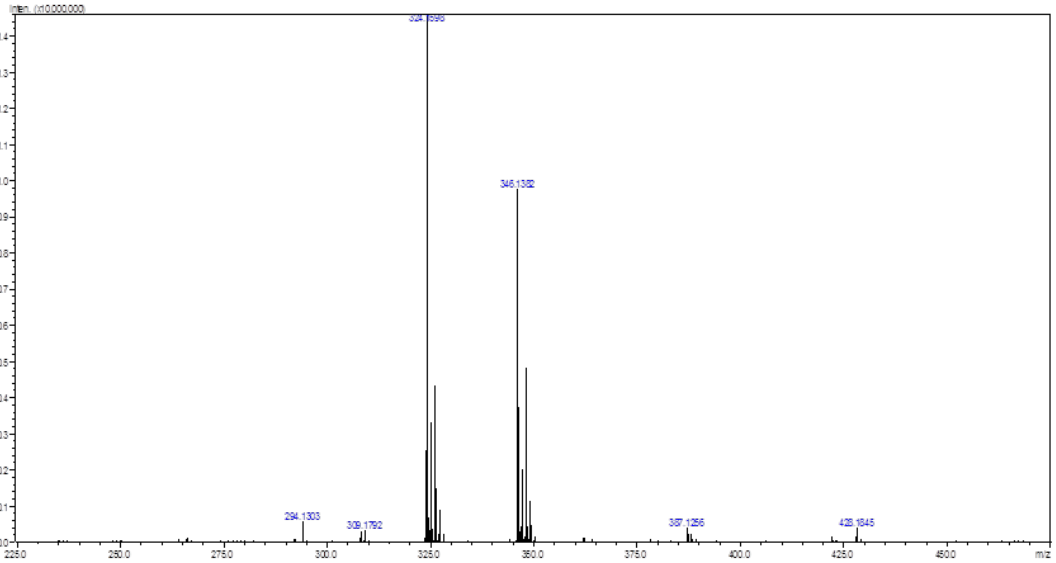

Fig. S66 ESI-MS of compound 5d

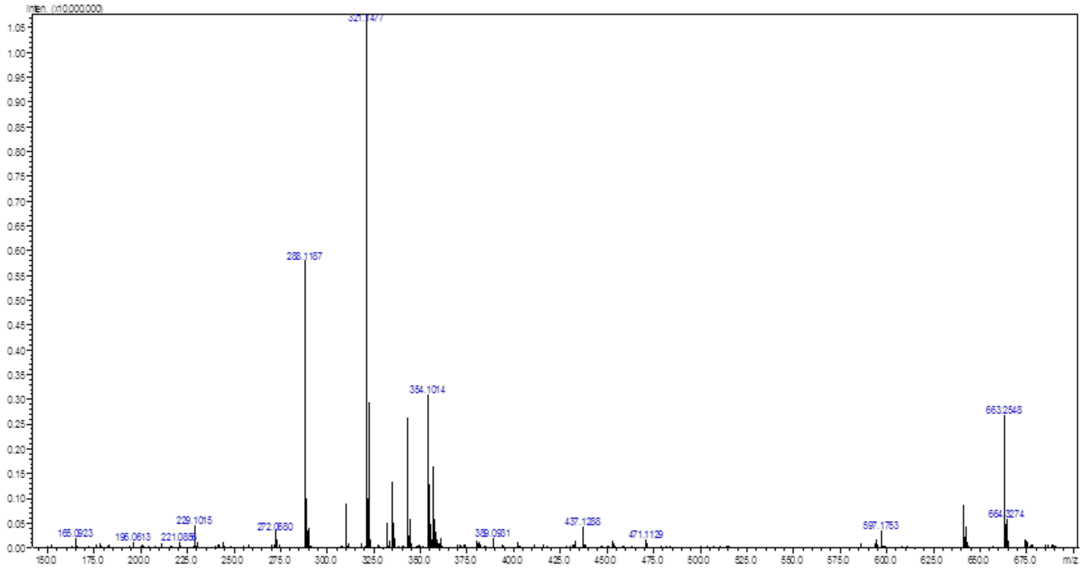

Fig. S67 ESI-MS of compound 5g

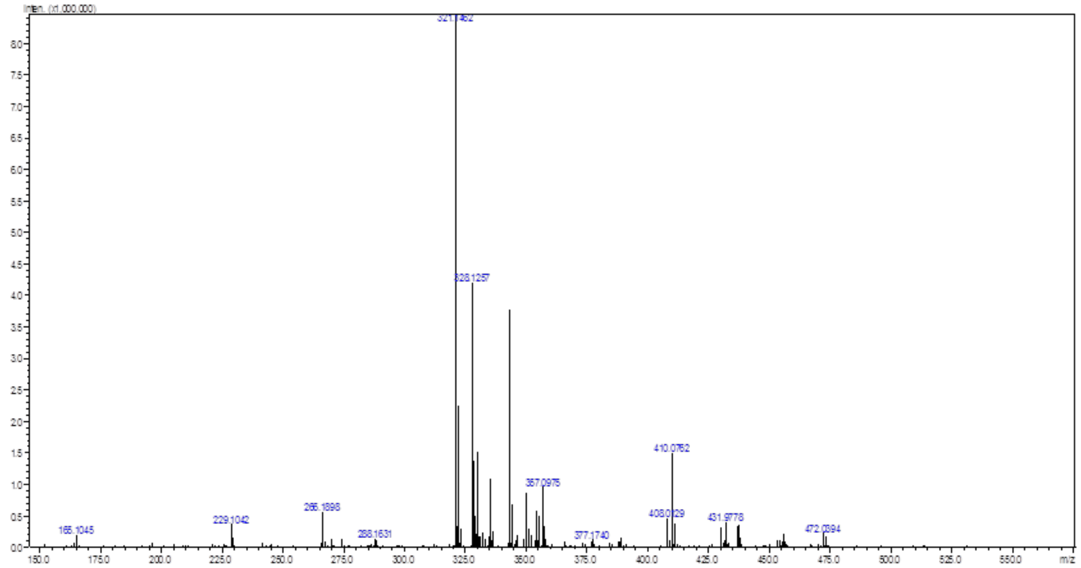

Fig. S68 ESI-MS of compound 5j

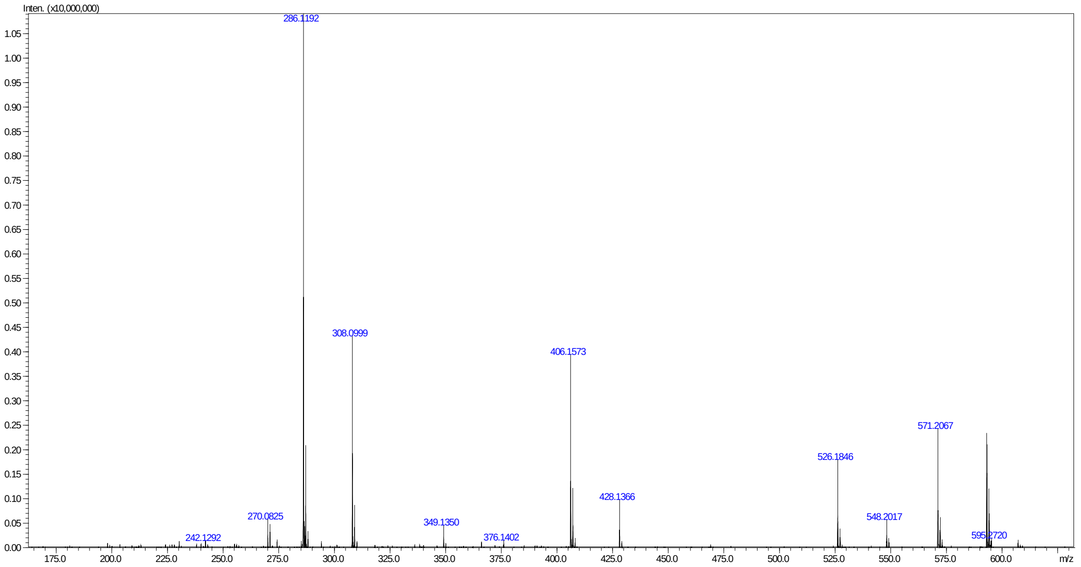

Fig. S69 ESI-MS of compound 6a

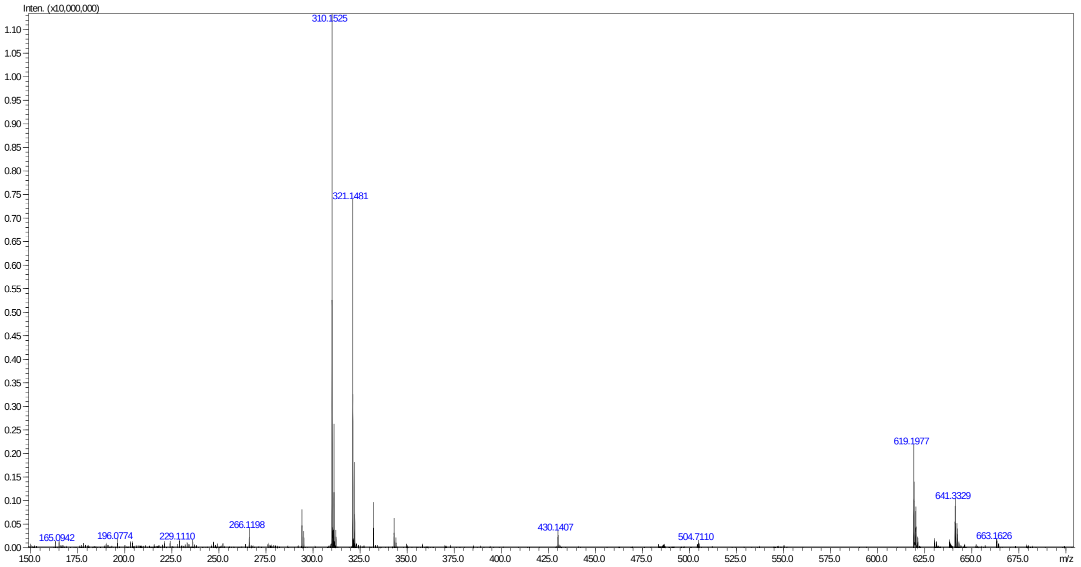

Fig. S70 ESI-MS of compound 6b

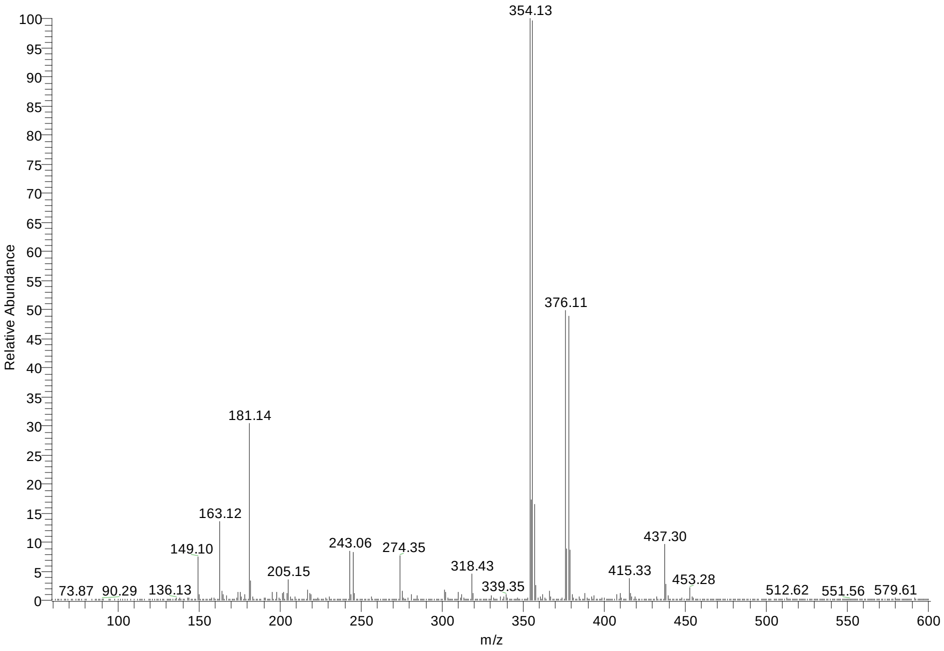

Fig. S71 ESI-MS of compound 6c

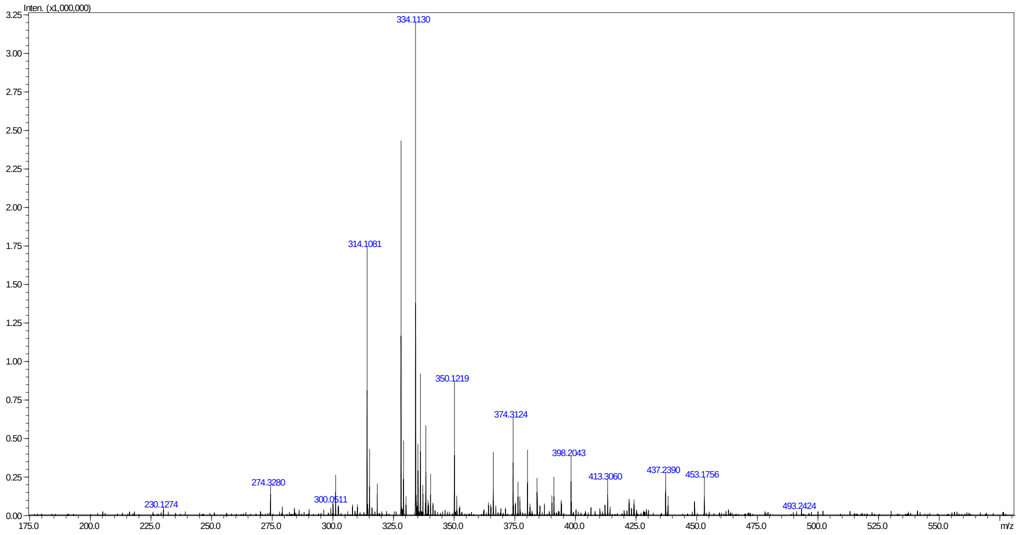

Fig. S72 ESI-MS of compound 6d

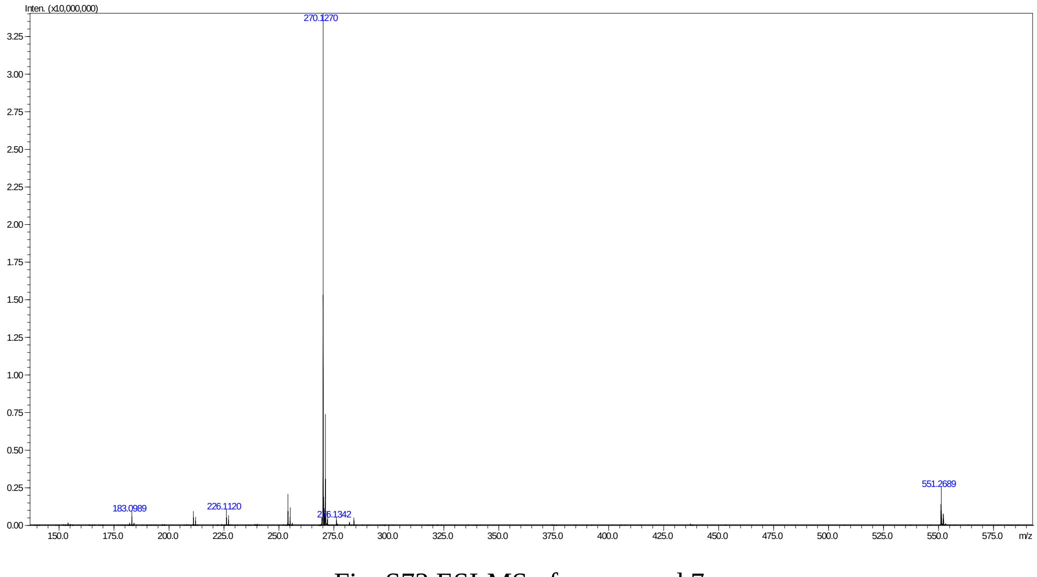

Fig. S73 ESI-MS of compound 7a

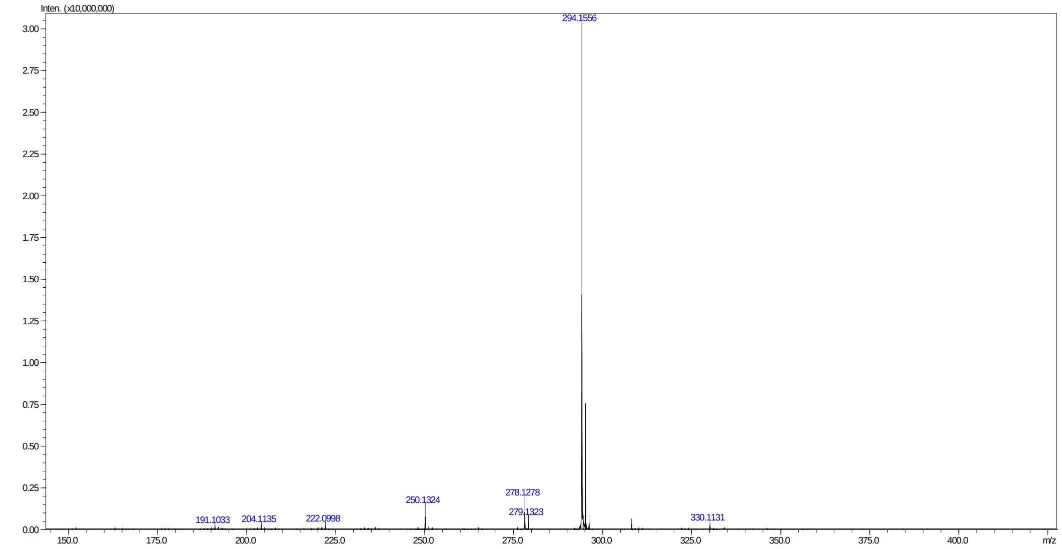

Fig. S74 ESI-MS of compound 7b

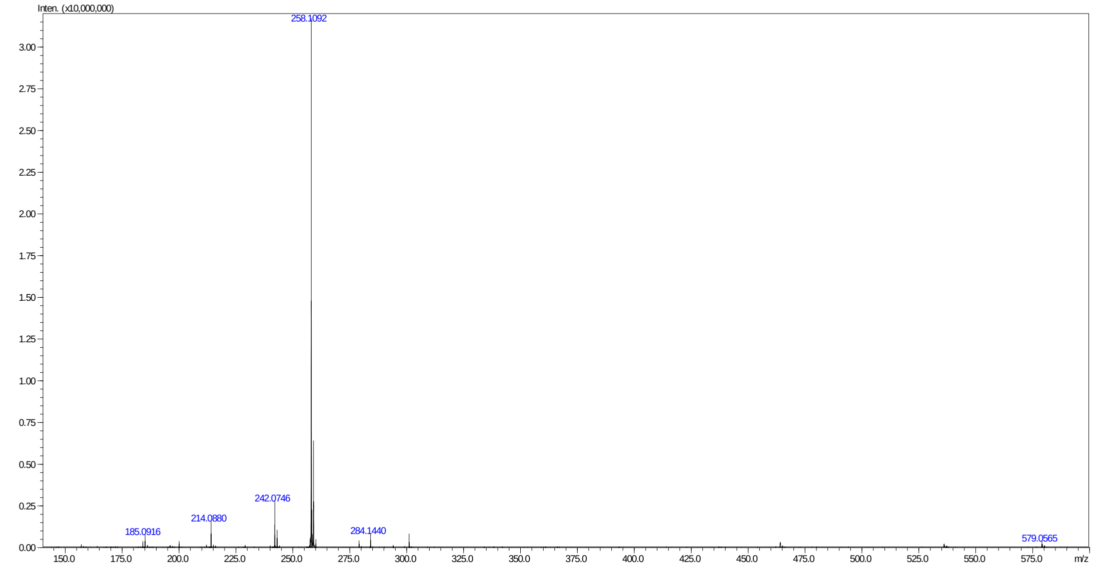

Fig. S75 ESI-MS of compound 7c

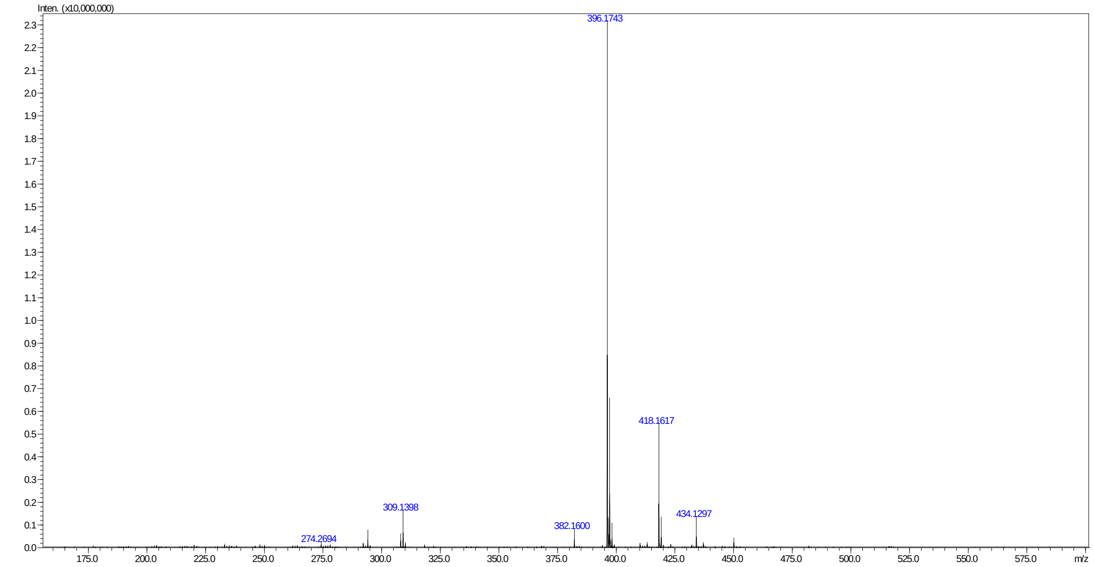

Fig. S76 ESI-MS of compound 8a

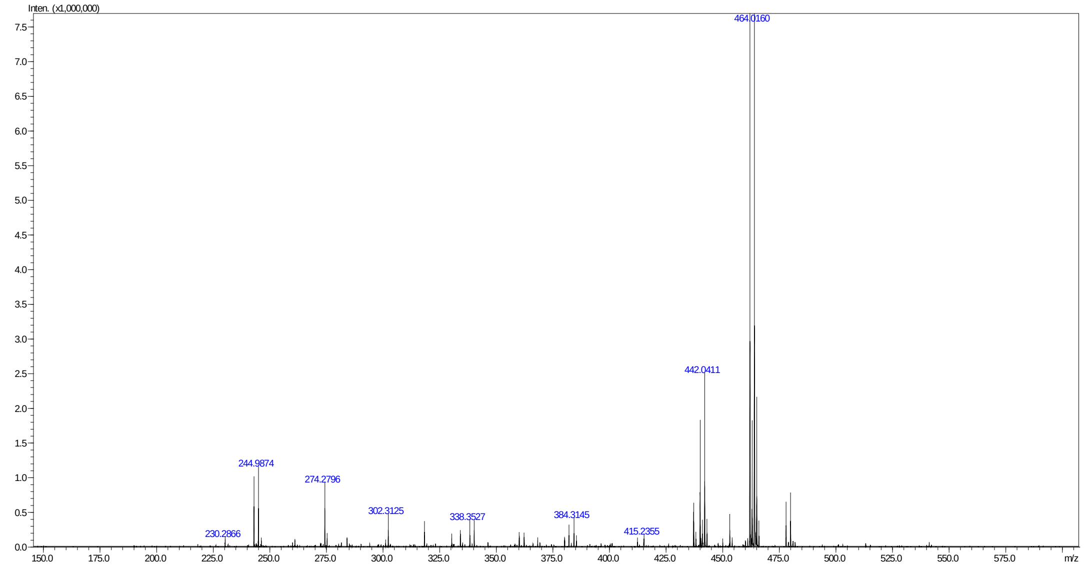

Fig. S77 ESI-MS of compound 8b

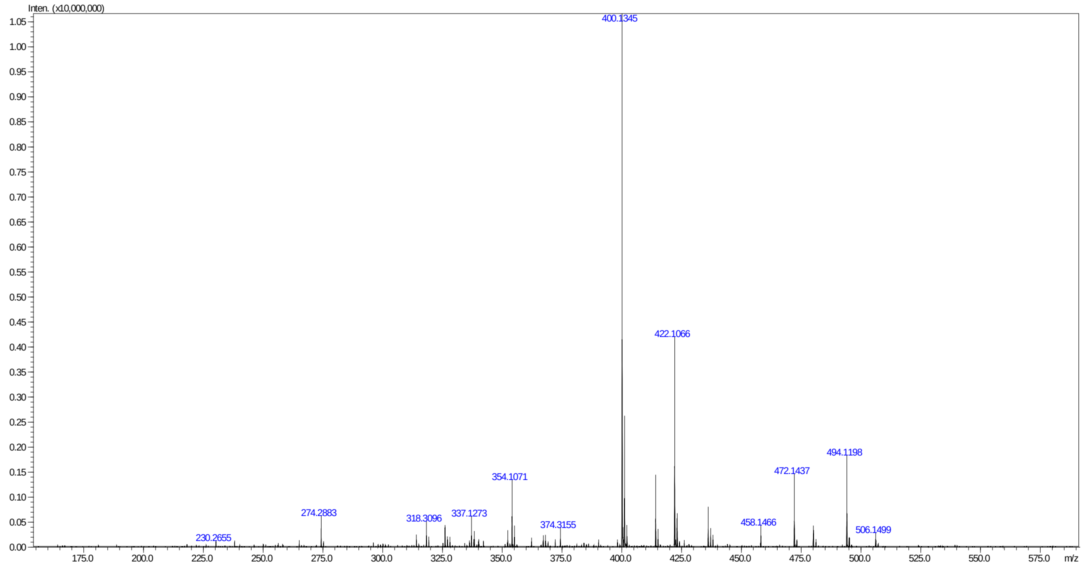

Fig. S78 ESI-MS of compound 8c

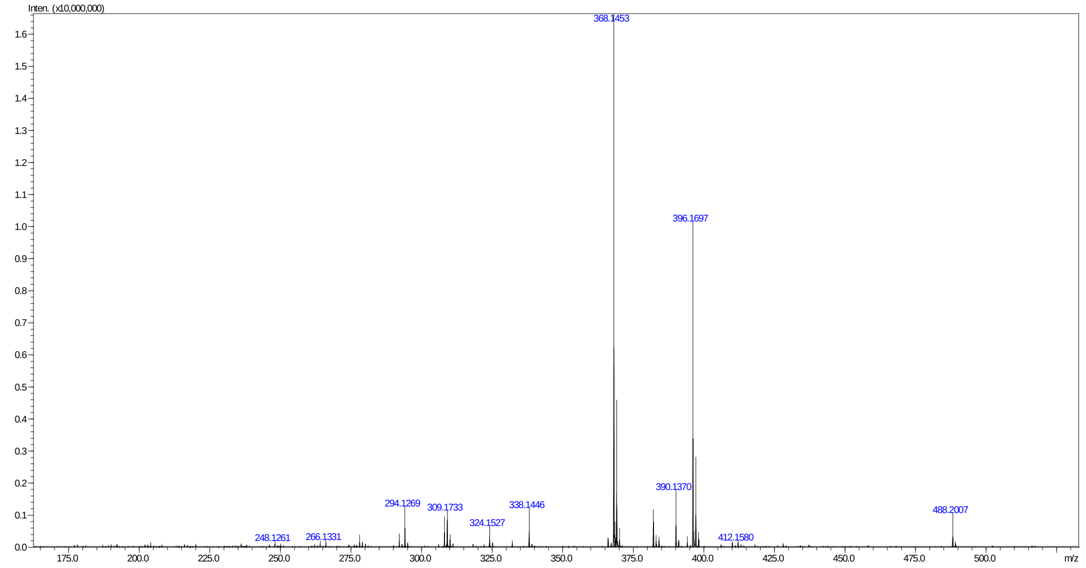

Fig. S79 ESI-MS of compound 9a

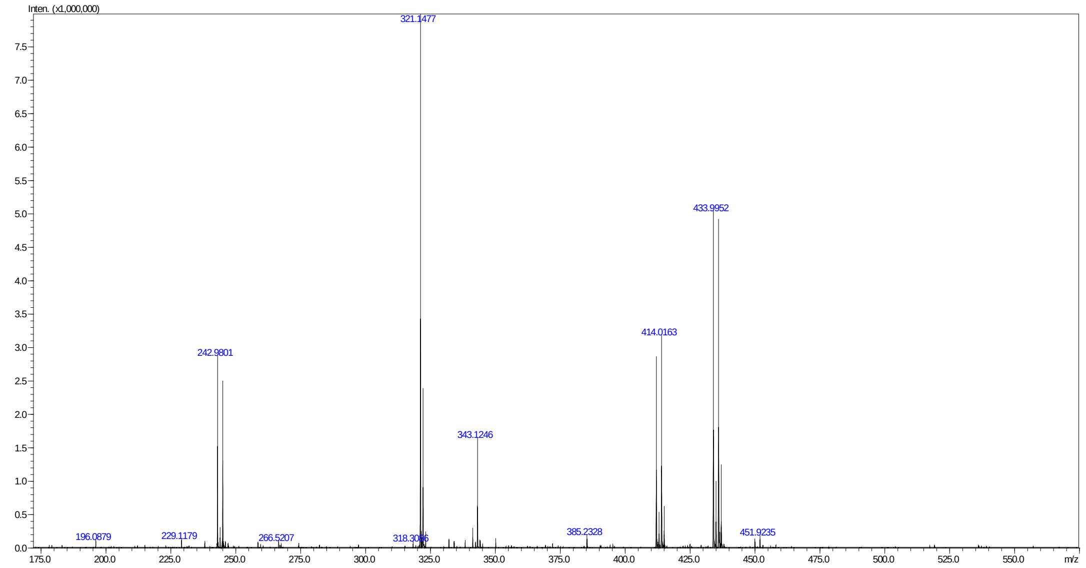

Fig. S80 ESI-MS of compound 9b

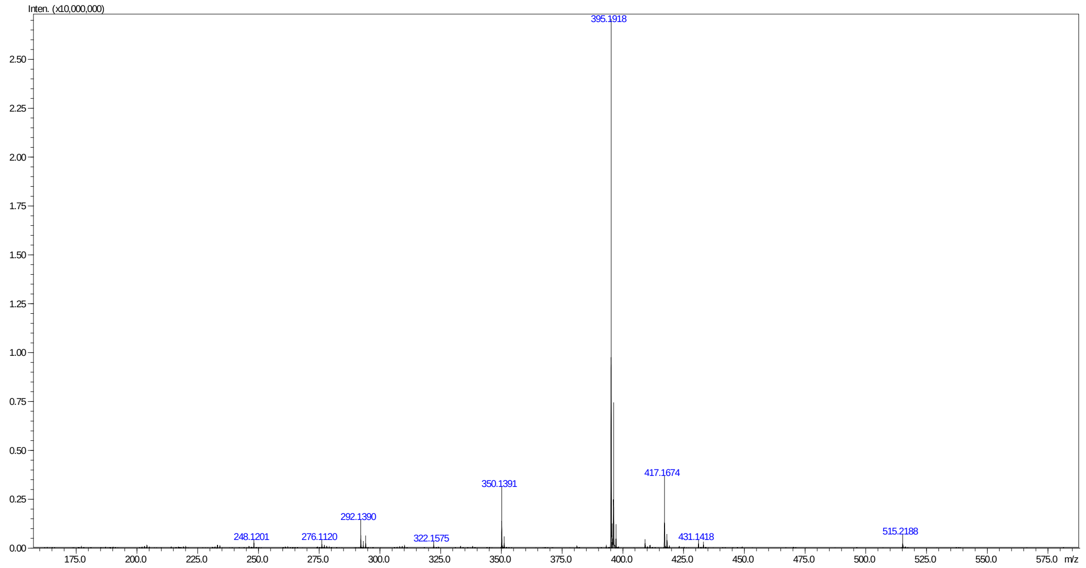

Fig. S81 ESI-MS of compound 10a

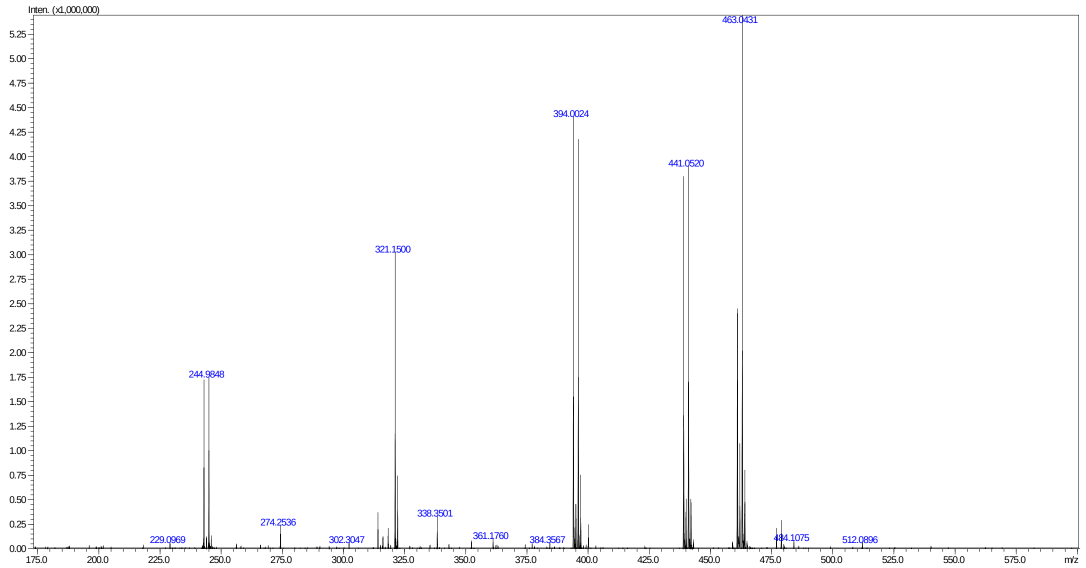

Fig. S82 ESI-MS of compound 10b

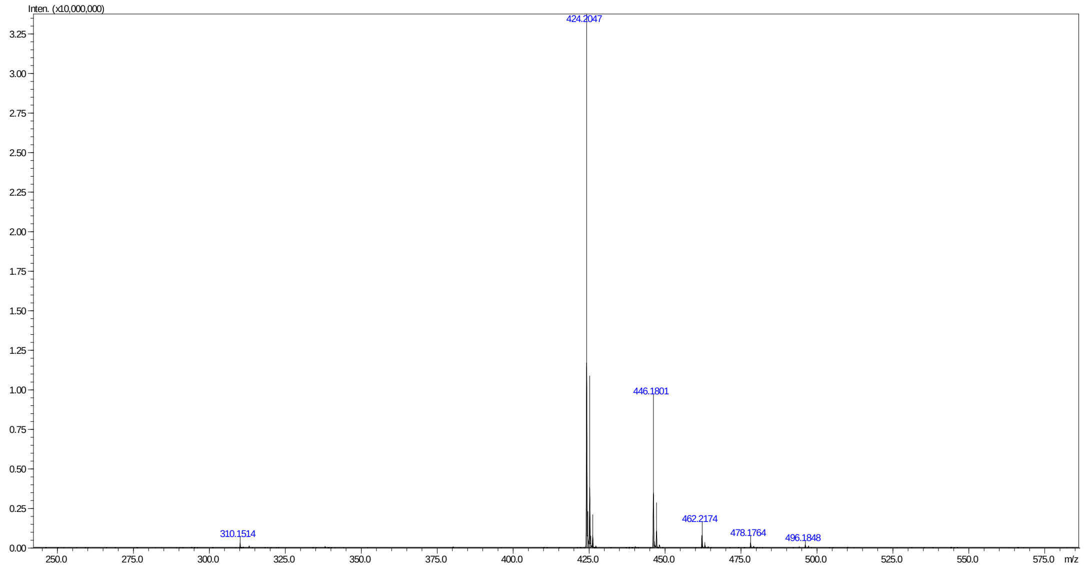

Fig. S83 ESI-MS of compound 11a

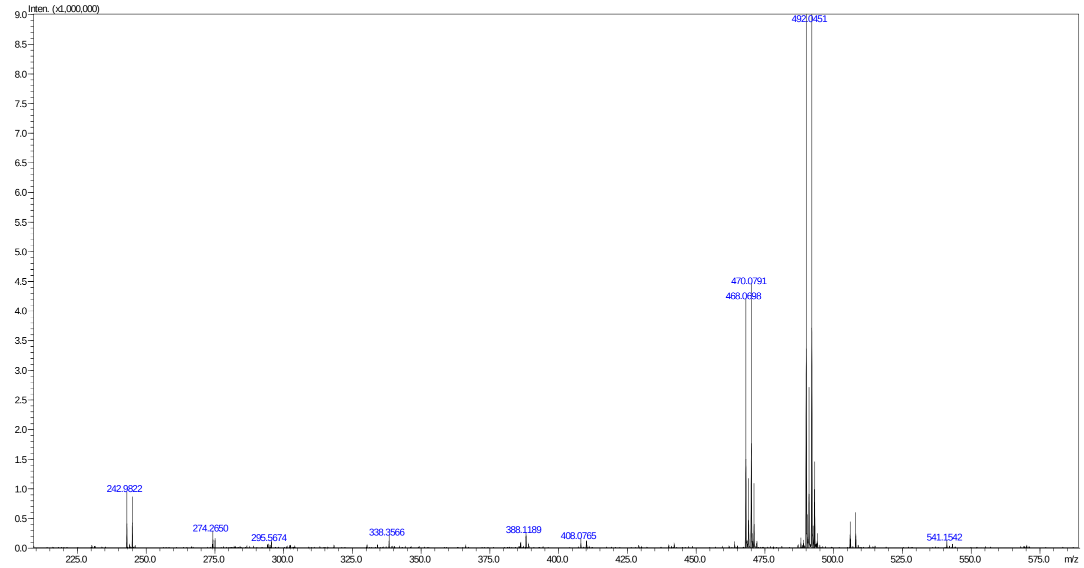

Fig. S84 ESI-MS of compound 11b

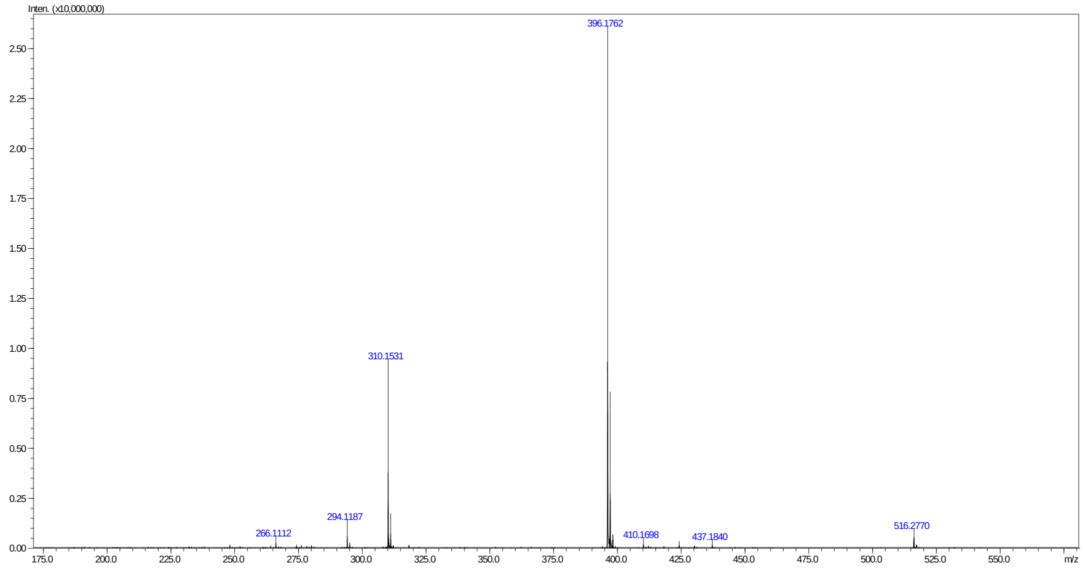

Fig. S85 ESI-MS of compound 12a

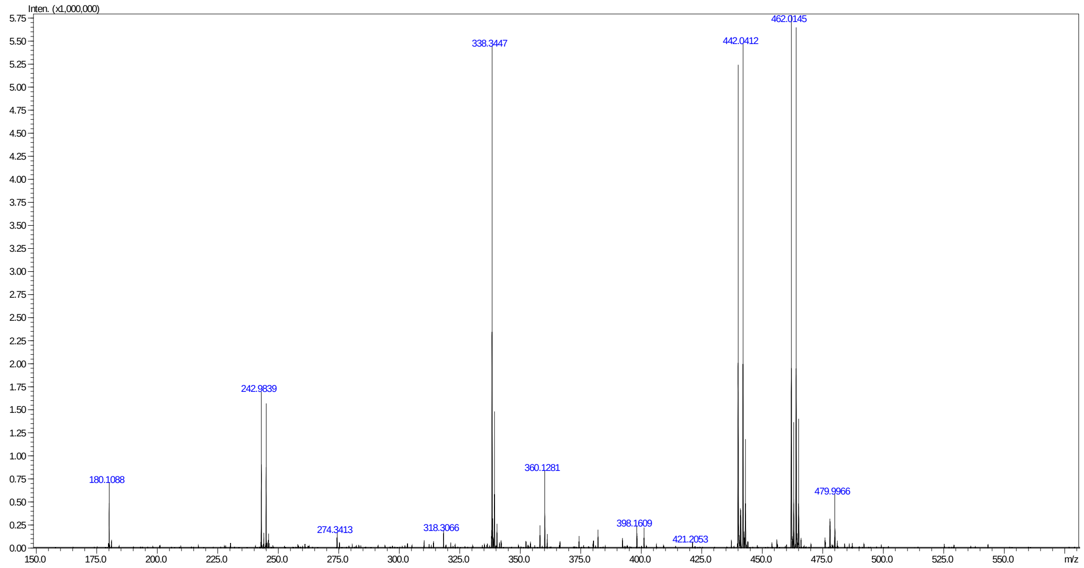

Fig. S86 ESI-MS of compound 12b

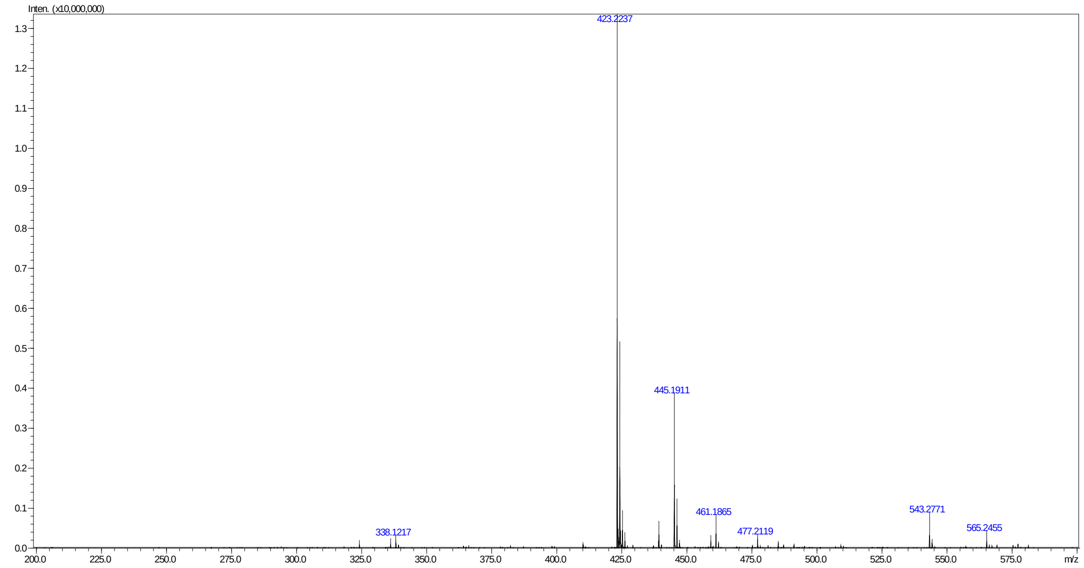

Fig. S87 ESI-MS of compound 13a

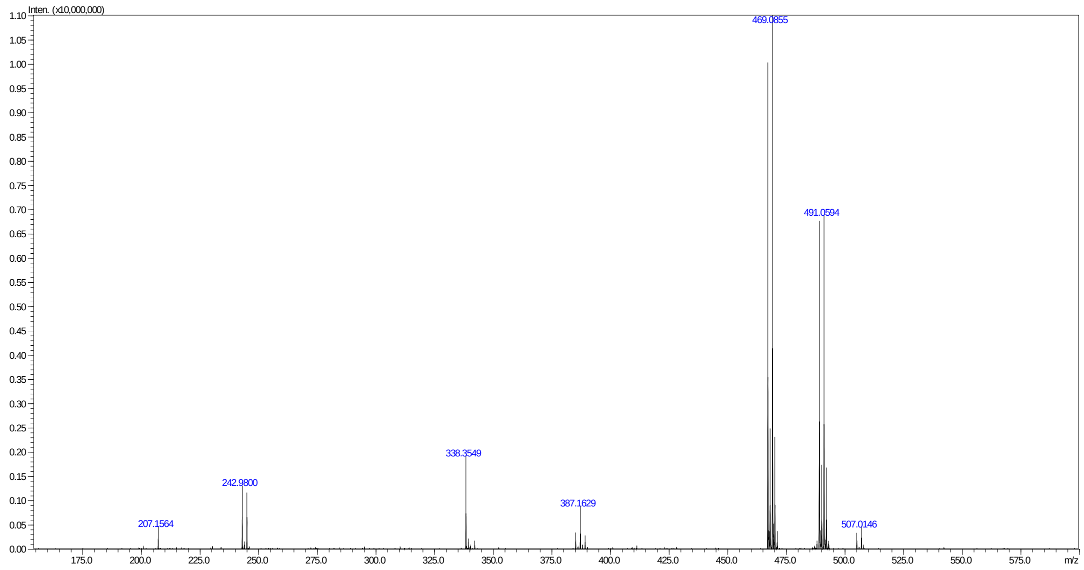

Fig. S88 ESI-MS of compound 13b

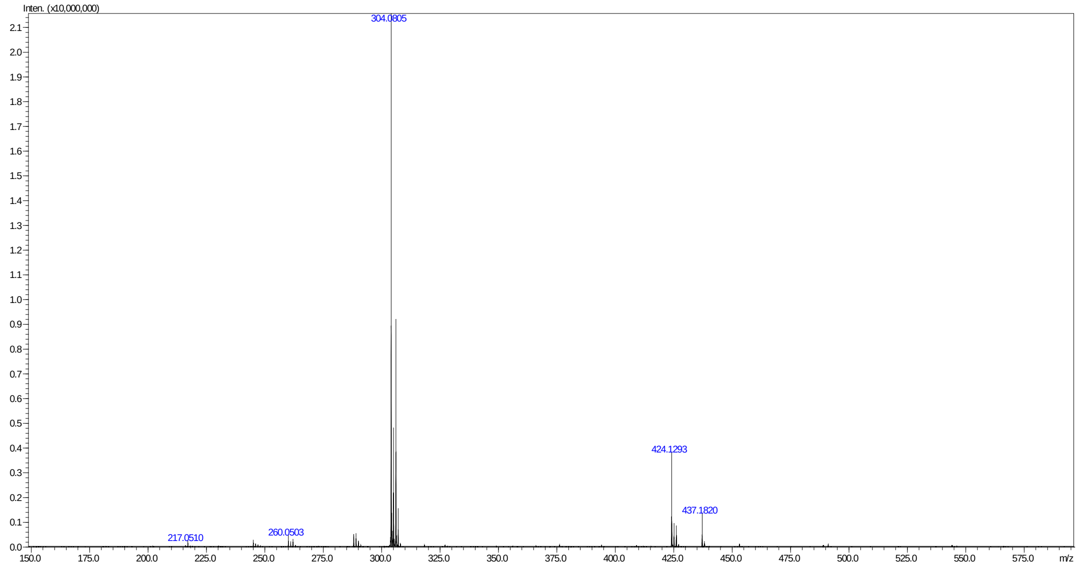

Fig. S89 ESI-MS of compound 14a

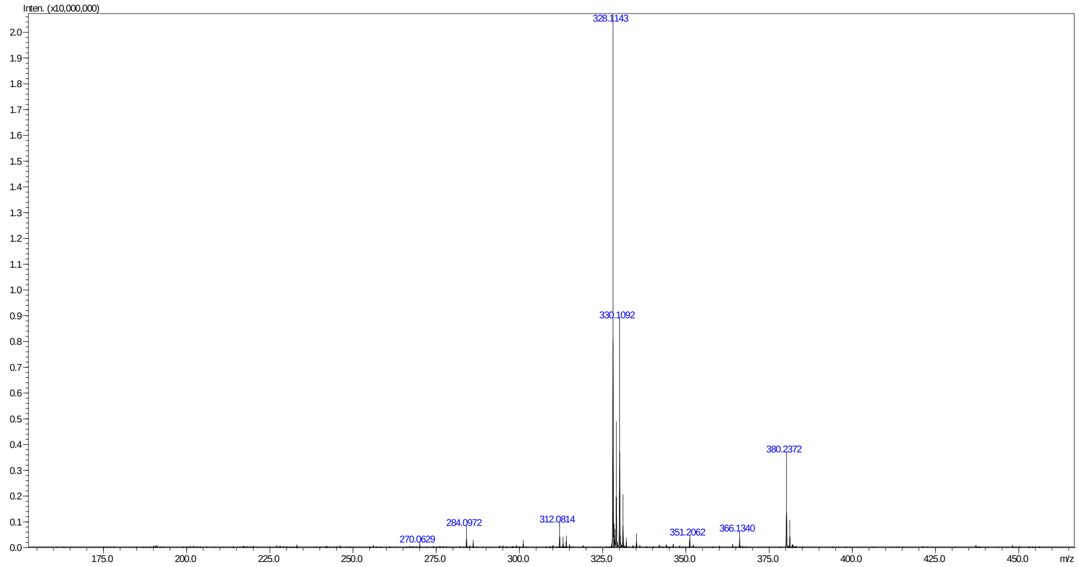

Fig. S90 ESI-MS of compound 14b

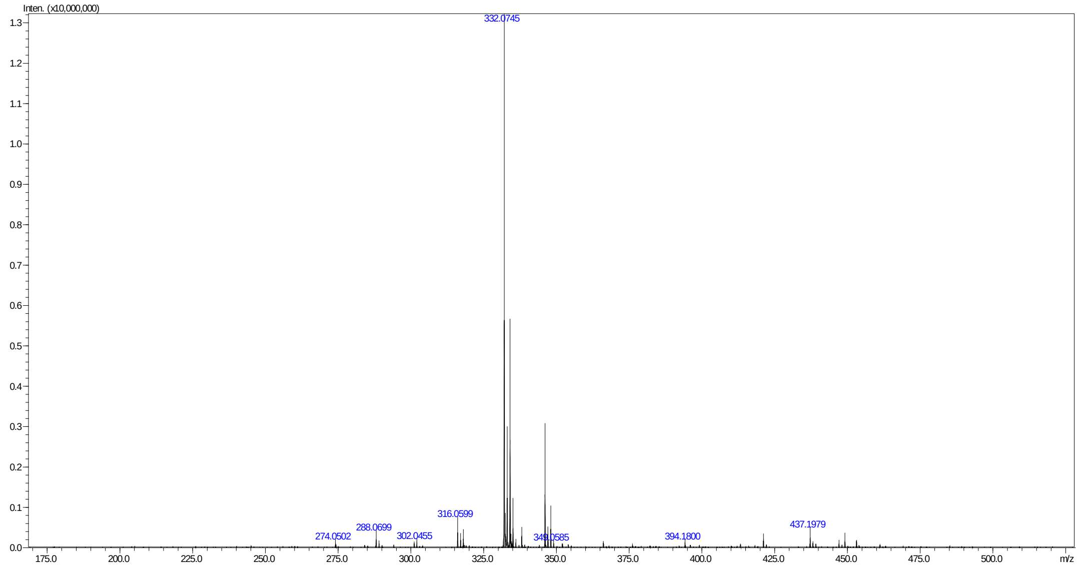

Fig. S91 ESI-MS of compound 14c

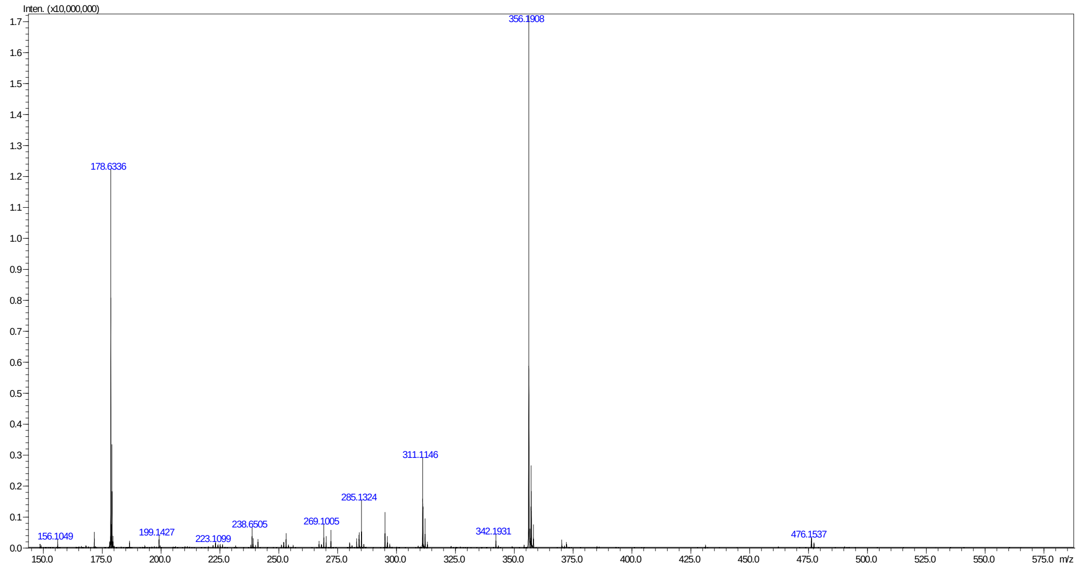

Fig. S92 ESI-MS of compound 15a

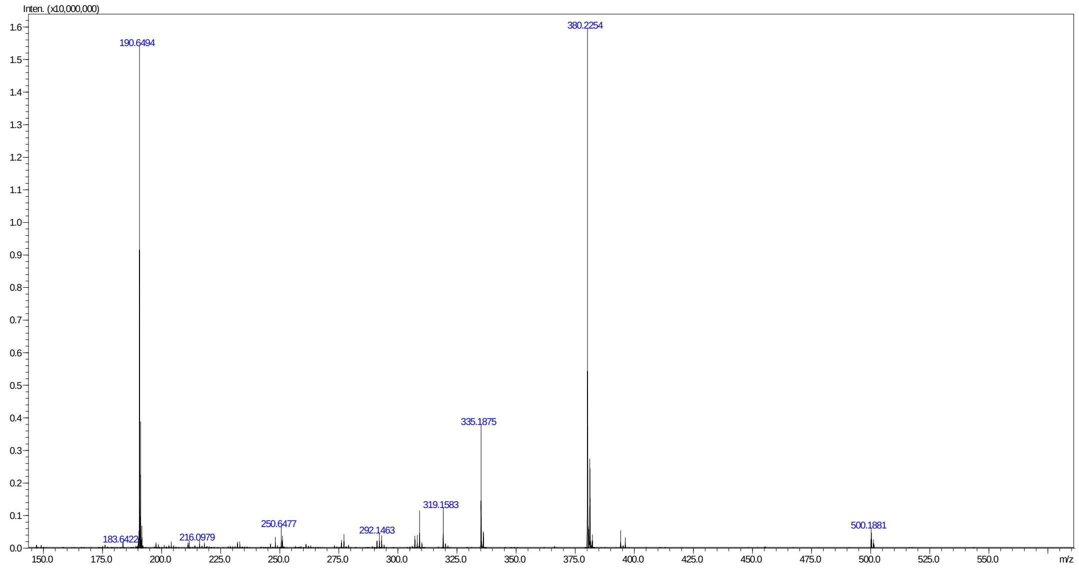

Fig. S93 ESI-MS of compound 15b

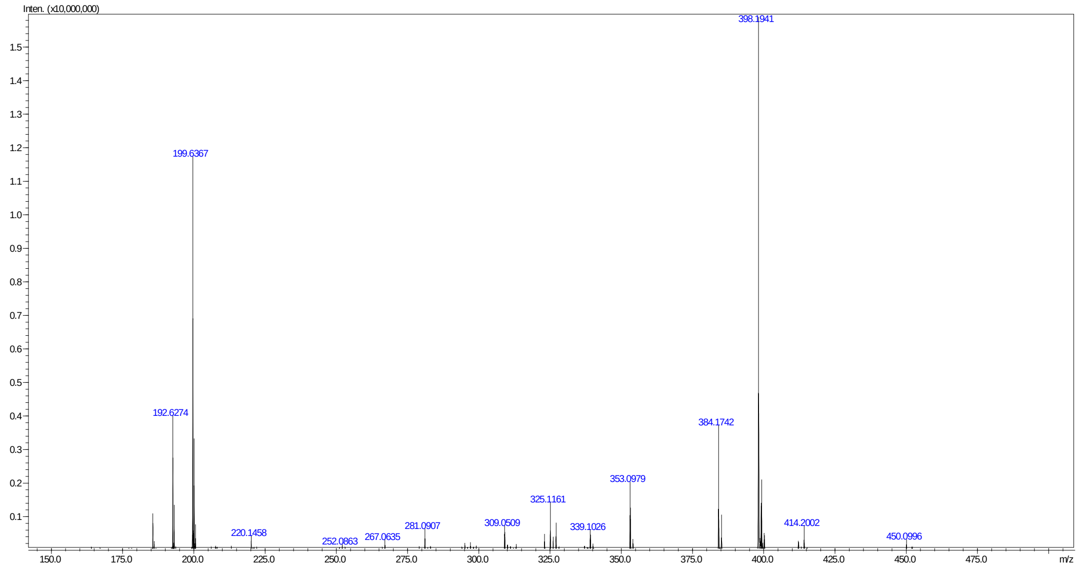

Fig. S94 ESI-MS of compound 15c

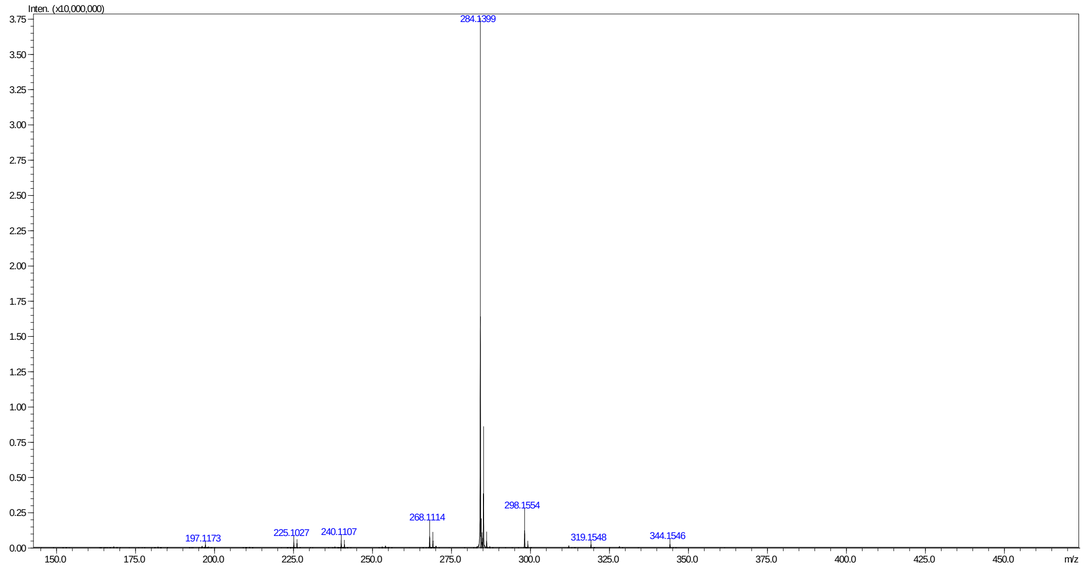

Fig. S95 ESI-MS of compound 16a

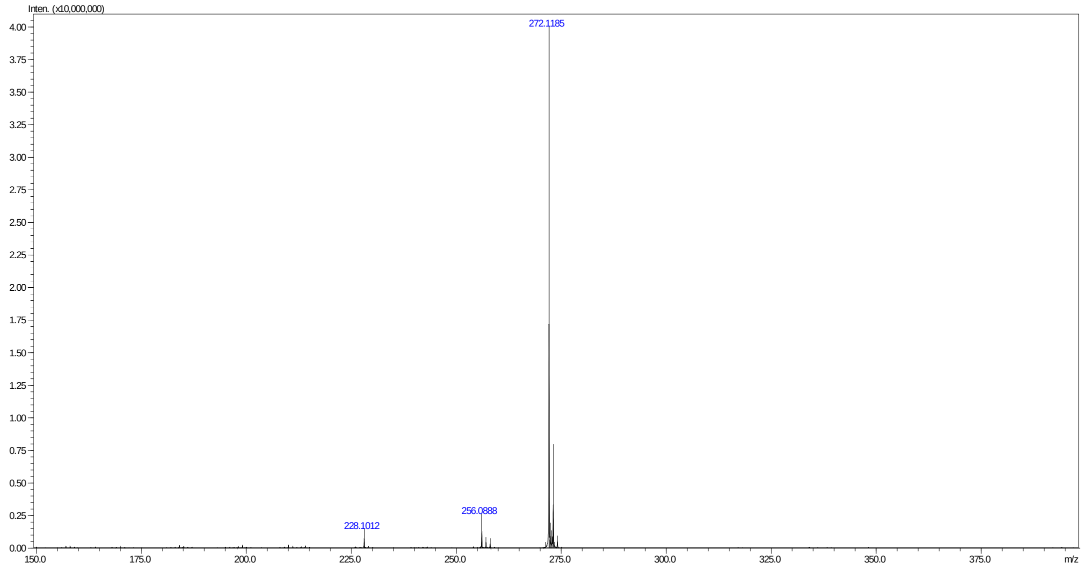

Fig. S96 ESI-MS of compound 16b
